# pH-mediated manipulation of the histidine brace in LPMOs and generation of a tri-anionic variant, investigated by EPR, ENDOR, ESEEM and HYSCORE spectroscopy†

**DOI:** 10.1039/d4sc04794j

**Published:** 2024-11-26

**Authors:** Julia Haak, Ole Golten, Morten Sørlie, Vincent G. H. Eijsink, George E. Cutsail

**Affiliations:** a Max Planck Institute for Chemical Energy Conversion Stiftstrasse 34–36 D-45470 Mülheim an der Ruhr Germany george.cutsail@cec.mpg.de; b Institute of Inorganic Chemistry, University of Duisburg-Essen Universitätsstrasse 5–7 D-45141 Essen Germany; c Faculty of Chemistry, Biotechnology and Food Science, NMBU - Norwegian University of Life Sciences N-1432 Ås Norway

## Abstract

Lytic Polysaccharide Monooxygenases (LPMOs) catalyze the oxidative depolymerization of polysaccharides at a monocopper active site, that is coordinated by the so-called histidine brace. In the past, this motif has sparked considerable interest, mostly due to its ability to generate and stabilize highly oxidizing intermediates during catalysis. We used a variety of advanced EPR techniques, including Electron Nuclear Double Resonance (ENDOR), Electron Spin Echo Envelope Modulation (ESEEM) and Hyperfine Sublevel Correlation (HYSCORE) spectroscopy in combination with isotopic labelling (^15^N, ^2^H) to characterize the active site of the bacterial LPMO *Sm*AA10A over a wide pH range (pH 4.0–pH 12.5). At elevated pH values, several ligand modifications are observed, including changes in the H_*x*_O ligand coordination, but also regarding the protonation state of the histidine brace. At pH > 11.5, the deprotonation of the two remote nitrogen nuclei of the imidazole moieties and of the terminal amine is observed. These deprotonations are associated with major electronic changes, including increased σ-donor capabilities of the imidazolates and an overall reduced interaction of the deprotonated amine function. This observation highlights a potentially more significant role of the imidazole ligands, particularly for the stabilization of potent oxidants during turnover. The presented study demonstrates the application of advanced EPR techniques for a thorough characterization of the active site in LPMOs, which ultimately sets a foundation for and affords an outlook on future applications characterizing reaction intermediates.

## Abstract 2

A lytic polysaccharide monooxygenase's ligands and their electronic influences are revealed by advanced electron paramagnetic resonance spectroscopies.

## Graphical abstract

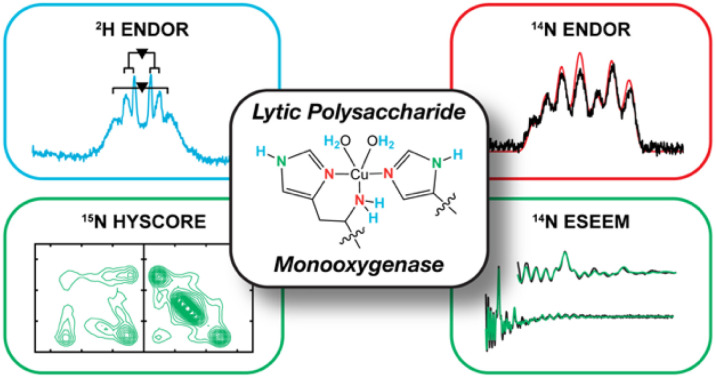

## Introduction

**Scheme 1:**
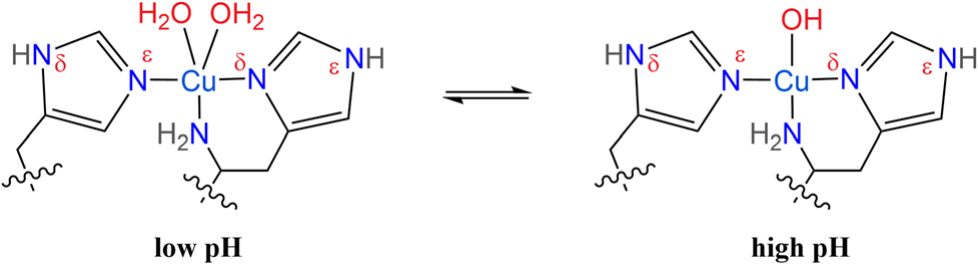
Structure of the histidine brace in AA10 LPMOs, showing the pH dependent coordination of water and hydroxo ions.

Lytic polysaccharide monooxygenases (LPMOs) are abundant enzymes that catalyze the oxygenation and subsequent cleavage of glycosidic bonds in recalcitrant polysaccharides like cellulose or chitin, at a common monocopper active site.^1–3^ This active site is commonly referred to as the “histidine brace”,^2^ comprised of a copper ion coordinated by two histidine residues (Scheme 1). One of these is the N-terminal histidine, which coordinates copper in a bidentate fashion through its terminal (primary) amine and the Nδ of its imidazole moiety, while the other histidine coordinates through the Nε of its imidazole ring. In LPMOs, the histidine brace is strictly conserved with almost orthogonal copper–nitrogen bonds [N–Cu(ii)–N angles between 87° and 106°; average value of 94°], and with Cu(ii)–N distances of approximately 2.0 Å and 2.1 to 2.2 Å, for the sp^2^-hybridized imidazole nitrogens and the sp^3^-hybrisized amine nitrogen, respectively.^4,5^

Similar 3N histidine brace motifs can be found in several other copper-binding proteins like CopC,^6–8^ X325,^9^ and most prominently, the Cu_B_ site of particulate methane monooxygenase (pMMO).^10^ The resemblance between the LPMO copper site and the Cu_B_ site of pMMO initially caught lots of attention as the two enzymes also show similarities in their reactivity. pMMO is able to activate C–H bonds in methane (105 kcal mol^−1^),^11^ in which the C–H bond is even stronger than those targeted by LPMOs (≳ 95 kcal mol^−1^).^12–14^ However, more recent research does not support the Cu_B_ site in the soluble domain as the site of catalysis, but presumes the active site to be located in one of the transmembrane subunits of pMMO, where no histidine-brace is formed.^15^ Likewise, studies have shown that CopC and X325 are not redox active.^16^ This lack of activity coincides with the presence of additional amino acids (*e.g.* His in pMMO) in the equatorial positions yielding a complete coordination sphere and no open sites for co-substrates to bind and react with the typical d_*x*^2^ – *y*^2^_ Frontier molecular orbital. These recent observations underpin the unique nature of the LPMO active site, and make these enzymes well suited to understand how the histidine brace tunes the copper site for reactivity, including the protein's rare use of a N-terminal amine.

In the resting state, the copper center of LPMOs exhibits an oxidation state of +2, which has to be reduced to Cu(i) prior to its reaction with an oxygen co-substrate. The question if molecular oxygen or hydrogen peroxide is the natural co-substrate of LPMOs is a topic of debate,^17^ but either case results in the generation of highly oxidizing intermediates, that help to overcome the high activation barrier for C–H activation in the polysaccharide substrate.^12,18,19^

The crucial importance of C–H bond activations in biology and industrial applications has inspired numerous studies of synthetic small molecule copper catalysts^20–25^ as well as theoretical investigations of C–H bond activation mechanisms.^12,26–28^ The synthetic community has long aimed to mimic the structural features and oxidation chemistry of LPMOs, with mixed success.^20,29–33^ Many of the monocopper biomimetic complexes characterized so far are only able to activate C–H bonds of moderate strength and do not approach the strong bond energies involved with C–H bond activation in the glycosidic linkages of cellulose or chitin.^12–14^ The only exceptions thus far are several Cu(iii)–OH complexes reported by Tolman and co-workers, which readily perform hydrogen atom abstraction (HAA) of organic substrates like cyclohexane with C–H bond energies of up to 99 kcal mol^−1^.^20–22^ The ability to do so was ascribed to both the basic nature of the Cu(iii)–OH core and the electron donating di-anionic carboxamide ligands that lower the redox potential of the Cu(iii)/Cu(ii) couple (and thereby stabilize the Cu(iii) state). Of note, many studies conclude that C–H bond activation by LPMOs involves either such a copper hydroxide, [Cu(iii)–OH]^2+^, or a copper oxyl, [Cu(ii)–O˙]^+^, species.

In this context, the potential deprotonation of the amine function of the N-terminal histidine in LPMOs has been previously discussed as it would mimic the anionic character of the biomimetic complexes by Tolman and co-workers.^2,5,21,34^ Computational studies suggest that the deprotonation of the amine could lower the Cu(ii)/Cu(iii) redox potential of intermediates by as much as ∼2 V.^12^ The p*K*_a_ values of Cu(ii)-amines have yet to be experimentally determined and previous DFT calculations of the amine p*K*_a_ value in LPMOs proved to have a strong solvent (dielectric constant) dependence, limiting the reliability of calculated values.^12^ However, it has been demonstrated that the p*K*_a_ values of primary amines coordinated to Cu(iii) can be as low as p*K*_a_ ∼8.8,^35^ making such deprotonation a conceivable possibility in the context of an enzyme under physiological conditions.

In 2016, Frandsen *et al.* showed that binding of an oligosaccharide substrate to *Ls*AA9A creates two distinct chemical environments for the two amine protons of the histidine brace, with one of them partaking in a hydrogen bond network.^36^ This would not only enhance the basicity of this proton but also create a pathway for proton transfer, that could help to deprotonate the amine function. In 2017, the X-ray and neutron structures of *Jd*AA10A revealed a mixture of ND_2_ and ND^−^ states of its primary amine at pH 7.0, which seemingly confirmed the feasibility of the deprotonation at physiological pH values.^37^ However, data interpretation in this study has been questioned^38^ and later neutron structures of another LPMO, *Nc*AA9D, which was first reduced and then reacted with molecular oxygen, showed a mixture of superoxo and hydroperoxo species with a fully deuterated amine function.^39^

Despite the intense focus on the primary amine, the possible influence of the two coordinating imidazole moieties has been largely undiscussed and their remote nitrogen nuclei are typically thought to be protonated.^38,39^ However, their deprotonation would – similarly to a deprotonated amine function – yield a more anionic ligand environment, potentially aiding to stabilize reactive intermediates.^29^ Indeed, it was computationally shown that the deprotonation of copper-imidazoles could lie possibly within physiological range,^40^ encouraging further consideration of this state in LPMOs. Clearly, the degree of anionic character of the copper environment and, in particular, the protonation state of the primary amine and the imidazole moieties, are important for understanding the functionality of LPMOs and, therefore, have direct repercussions on the chemistry of LPMO-inspired synthetic complexes. However, due to the so far negligible experimental insight into p*K*_a_ values in copper-amines and copper-imidazoles, questions regarding the protonation state of the individual functional groups in the histidine brace remain open.

Such information may be extracted through the characterization of LPMOs in dependence of the pH. Previously, the AA10 LPMOs *Bl*AA10 (ref. 41) and *Pl*AA10 (ref. 42) showed stability under basic conditions and exhibited pH dependent EPR spectroscopic trends that were associated with changes in the primary coordination sphere, more specifically with water/hydroxo ligand exchanges around pH 8–10 (Scheme 1).^42^ These assignments were supported computationally, but in general, EPR spectroscopy lacks the resolution to study the weaker proton hyperfine interactions of water and hydroxo ligands, precluding their direct observation. At an even further elevated pH, an EPR spectrum associated with the formation of an additional species was observed for *Bl*AA10.^41^ The authors hypothesized that this could be related to the deprotonation of the primary amine of the histidine brace to form an azanido ligand (R–NH–; Scheme 1).^41^ However, again the presented EPR spectrum itself did not offer sufficient resolution to allow for the determination of protonation states from proton or nitrogen hyperfine couplings.

The electron-nuclear hyperfine couplings between the copper centered unpaired electron and the various NMR-active nuclei in its vicinity, offer information regarding metal–ligand covalencies, distances and geometries. Their wealth of information makes the hyperfine interactions of ligand nuclei an attractive target for investigation. Although EPR spectroscopy may lack the resolution to detect such often weaker interactions, we have employed a range of advanced EPR techniques, including ENDOR, ESEEM and HYSCORE spectroscopies to accurately determine hyperfine interactions of the Histidine brace. Such experiments are operated at a fixed magnetic field position, where a subset of molecular orientations is excited and their nuclear transitions detected. Maximum information is obtained when spectra are collected at multiple magnetic field positions, producing a 2D magnetic field-nuclear frequency pattern, which allows to determine full hyperfine (and nuclear quadrupole) tensors. For LPMOs this could help to discriminate between waters and hydroxos, determine protonation states and differentiate between the three nitrogen nuclei of the histidine brace, as well as assessing their individual Cu–N covalencies.

Despite all that, for LPMOs, advanced EPR techniques have had only limited applications or were applied with narrow focus, meaning that not even in the resting state the coordinating ^14^N hyperfine interactions of the individual ligands, and therefore a measure for the copper–nitrogen covalencies, have been determined by such high-resolution techniques. Ultimately, retrieving this information by defining the hyperfine (and quadrupole interactions) of the nuclei constituting the histidine brace is especially critical when trying to discriminate between the spectroscopic signature of the resting state and possible intermediates in future studies, as differentiation by EPR alone can be challenging or, in many cases, unfeasible.

To close the addressed knowledge gaps, we have used advanced EPR techniques, such as ENDOR, ESEEM and HYSCORE spectroscopies, to characterize the well-studied^43–47^ bacterial AA10 LPMO *Sm*AA10A from *Serratia marcescens* (also known as CBP21), over a wide pH range (pH 4.0–12.5). This revealed the deprotonation of not just the amine function, but also the imidazole moieties of the two coordinating histidine residues at elevated pH, accompanied by major electronic changes. The ability to manipulate an LPMO over such a wide pH range offered us the opportunity to investigate the structural and electronic tuning of the copper active site dependent on the protonation state of the primary amine and imidazole groups. Thus, this study tests the chemical flexibility of the histidine brace, exploring its variability and therefore its stability at extreme conditions outside of the physiological range. It also examines what its spectroscopic footprint looks like before and after deprotonation and how the bonding situation within the histidine brace changes upon the deprotonation events, and discusses how the three coordinating moieties may influence potential intermediates during turnover. Unravelling these essential structural and electronic properties of LPMO active sites provides deeper insight into the copper site's electronic tuning, and creates a foundation for future advanced EPR spectroscopic studies of LPMOs.

## Experimental

### Protein expression

^14^N *Sm*AA10A (CBP21) was expressed as previously described.^44^ In brief, 1L LB medium supplemented with 50 μg mL^−1^ ampicillin in a 1.8 L shaker flask was inoculated with a glycerol stab containing One Shot™ BL21 Star™ (DE3) cells (Invitrogen, Waltham MA, USA) harboring the *cbp*21-containing pRSETB vector. After inoculation, the cell culture was grown for 16 hours at 37 °C with 200 rpm agitation, during which, due to the leaky nature of the promoter in the pRSETB vector, expression was constitutive. ^15^N *Sm*AA10A was expressed in a similar manner, using M9 minimal medium containing ^15^NH_4_Cl (Sigma-Aldrich, Saint-Louis, MO, USA) as the nitrogen source and with a longer growth period of 20 hours.

### Protein purification

The same purification method was used for both ^14^N and ^15^N *Sm*AA10A. The periplasmic fraction containing *Sm*AA10A was isolated using a cold osmotic shock method^48^ and filtered through a 0.22 μm filter before adjusting the solution to the binding buffer (50 mM Tris–HCl pH 8.0, 1 M NH_4_SO_4_). The purification was performed using chitin bead affinity chromatography (NEB, Ipswich, MA, USA) by equilibrating a 15 mL self-packed column with binding buffer prior to loading 30 mL of the adjusted periplasmatic extract. Unbound protein was washed out with 5 column volumes of binding buffer before *Sm*AA10A was eluted using 20 mM acetic acid. Protein purity was assessed by SDS-PAGE and fractions containing pure protein were pooled before buffer exchanging into 50 mM Tris–HCl, pH 8.0, using a 10 kDa Amicon® Ultra-15 centrifugal filter unit (Merck, Darmstadt, Germany).

### Copper saturation

Copper saturation of the LPMO was performed by incubating pure enzyme with three-fold molar excess of CuSO_4_ at 4 °C for 30 minutes, followed by removal of excess free copper by several rounds of concentration and dilution into a multicomponent buffer [5 mM MES (2-(*N*-morpholino)-ethane sulphonic acid; Carl Roth, Karlsruhe, Germany), 5 mM HEPES (*N*-2-hydroxyethylpiperazine-*N*′-2-ethane sulphonic acid; Carl Roth, Karlsruhe, Germany), 5 mM CHES (2-(cyclohexylamino)ethanesulfonic acid; Sigma-Aldrich, Saint-Louis, MO, USA), 5 mM CAPS (cyclohexylamino propanesulphonic acid; Carl Roth, Karlsruhe, Germany)] at selected pH values using a 10 kDa Amicon® Ultra-15 centrifugal filter unit (Merck, Darmstadt, Germany), achieving a minimum dilution factor of 10^6^. ^15^N-enriched *Sm*AA10A was additionally ^63^Cu-enriched to help simplify the EPR spectra and reduce broadening (natural abundances ^63^Cu: 69.2%, ^65^Cu: 30.8%; both *I* = 3/2). It was prepared using ^63^CuSO_4_, which was obtained through dissolving 0.057 g ^63^Cu-enriched copper foil (99.9% isotopic enrichment, Campro Scientific GmbH, Berlin) in mixture of 0.3 mL conc. H_2_SO_4_ and 1.0 mL 30% H_2_O_2_, followed by stirring at room temperature overnight. Precipitated copper sulphate was filtered off, washed with ethanol and dried.

pH values were adjusted either with an InLab Versatile Pro or an InLab Micro Pro-ISM pH electrode (Mettler-Toledo, Columbus, United States), calibrated by four-point calibration in the range of pH 4 to 13. pH values in deuterated solvent were measured with the same pH electrode without a conversion factor, using the “cancel-out approach” reported elsewhere.^49,50^ The validity of this approach was confirmed by EPR spectroscopy, showing the same responses for deuterated and non-deuterated samples (Fig. S1†).

### Chitin degradation by *Sm*AA10A

Chitin degradation experiments were performed in 2.0 mL microtubes incubated at 40 °C in an Eppendorf ThermoMixer C (Eppendorf, Hamburg, Germany) set to 850 rpm agitation. Reactions were carried out by incubating 1 μM *Sm*AA10A with 10 g L^−1^ squid pen β-chitin milled to a particle size of 75–200 μm (France Chitin, Orange, France; Batch 20140101), in 50 mM Tris–HCl, pH 7.0, and initiated by addition of ascorbate to a final concentration of 1 mM. *Sm*AA10A stock solutions of 100 μM were stored in the multi-component buffer (50 mM MES, HEPES, CHES and CAPS) at pH 6.5, 11.5 or 12.5 for either 16 hours or 2 minutes prior to diluting to the final concentration of 1 μM in the reaction.

The reactions were terminated by filtering sample aliquots through a 0.45 μm MultiScreen™ 96-well filter plate (Merck, Darmstadt, Germany) before transferring the filtrate to microtubes. Product analysis was simplified by incubating the product mixture with 1 μM of chitobiase (*Sm*CHB) for 16 hours at 37 °C, to degrade oxidized soluble products to the oxidized dimer (GlcNAcGlcNAc1A) and the native monomer (GlcNAc), as described previously.^51^

### Quantification of oxidized chitin oligosaccharides

The oxidized dimer (GlcNAcGlcNAc1A) was quantified by injecting 8 μL samples on an Ultimate 3000 RSLC (Dionex, Sunnyvale, CA, USA) equipped with a 100 × 7.8 mm Resex RFQ – Fast Acid H^+^ (8%) column (Phenomenex, Torrance, CA, USA) employing an isocratic gradient of 5 mM sulfuric acid at a flow rate of 1 mL min^−1^. In-house standards of the oxidized dimers were created by incubating *N*-acetyl chitobiose (Megazyme, Bray, Ireland; 95% purity) with a chitooligosaccharide oxidase from *Fusarium graminearium* as previously described.^51,52^

### Melting point analysis

The melting temperature of *Sm*AA10A at pH 6.5, 11.5 and 12.5 was determined by applying a temperature gradient from 25–98 °C at a rate of 1.5 °C min^−1^ in a StepOnePlus real time PCR (ThermoFisher Scientific, Waltham, MA, USA). Reactions were carried out by mixing *Sm*AA10A to a final concentration of 50 μM in 50 mM multi-component buffer with 1x SYPRO® orange dye (Thermo Fisher Scientific, Waltham, MA, USA). During the unfolding of *Sm*AA10A the SYPRO® orange dye will interact with the hydrophobic core, which will quench fluorescence of the dye. The first derivative of the fluorescence trace allows determination of the melting temperature.

### Sample preparations for EPR measurements

Samples for EPR studies were prepared in custom made 4 mm O.D. (X-band EPR) or 2.8 mm O.D. (Q-band EPR; Q-band ENDOR; X-band ESEEM) Quartz EPR tubes. Subsequently, they were flash frozen in liquid nitrogen and stored at 77 K.

### X-band EPR

Continuous-wave X-band (∼9.46 GHz) EPR spectra were measured on a Bruker MS5000 spectrometer equipped with a liquid nitrogen cryostat. All spectra were collected at 100 K with the following parameters: sweep time = 300 s, modulation frequency = 100 kHz, modulation amplitude = 4 G, effective time constant = 0.05 s, effective number of points = 4000, number of scans = 1.

### Q-band EPR

Two-pulse (Hahn) echo detected Q-band EPR spectra were collected with a π/2–*τ*–π–*τ*–echo pulse sequence on a Bruker Elexsys-580 using a home-built up/down Q-band pulse conversion accessory^53^ and home-built TE_011_ microwave resonator.^54^ A temperature of 12 K was maintained with an Oxford C-935 liquid helium cryostat. The spectra were collected with the following parameters: π = 20 ns, *τ* = 400 ns, repetition rate = 5 to 10 ms (depending on the sample); shots per point = 10; number of points = 4096; number of scans = 1–10.

### Q-band ENDOR

Q-band ENDOR spectra were obtained with the same set up as described for the Q-band EPR experiments. Davies ENDOR^55^ was collected with the following microwave pulse sequence, π–*T*_RF_–*t*_wait_–π/2–*τ*–π–*τ*–echo, where the radio frequency (RF) pulse is applied during time *T*_RF_. The spectra were collected at 8 K and the following parameters were used: π = 80 ns; *τ* = 400 ns; *T*_RF_ = 30 μs; *t*_wait_ = 2 μs; repetition rate = 7–20 ms (depending on the sample); shots per point = 1; number of points = 1200 (^1^H), 1600 (^14,15^N); number of scans = ∼15–1000 (depending on sample, magnetic field position, and isotope). Mims ENDOR^56^ spectra of deuterated samples were obtained with the Mims sequence π/2–*τ*–π/2–*T*_RF_–*t*_wait_–π/2–*τ*–echo at 8 K and the following parameters: π/2 = 16 ns; *τ* = 220 ns; *T*_RF_ = 30 μs; *t*_wait_ = 2 μs; repetition rate = 10 ms; shots per point = 1; number of points = 512; number of scans = ∼20–300 (depending on sample and field position). In all Davies and Mims ENDOR experiments, the RF was randomly hopped^57^ without phase cycling.

The ENDOR spectrum for nuclei with a nuclear spin of *I* = 1/2 (^1^H, ^15^N) exhibits signal pairs at the frequencies

1 *ν*_±_ = |*ν*_n_ ± *A*/2|

where *A* is the effective, orientation-selective hyperfine coupling and *ν*_n_ is the Larmor frequency of the respective nucleus at the given magnetic field position. Therefore, signals of weakly coupled nuclei (|*A*| < 2|*ν*_n_|) appear centered at the nuclear Larmor frequency split by their effective hyperfine couplings, whereas signals of strongly coupled nuclei (|*A*| > 2|*ν*_n_|) are detected at half of their effective hyperfine coupling and split by twice their Larmor frequency.

Nuclei of higher nuclear spin possess a non-spherical nucleus which results in an additional nuclear quadrupole interaction (nqi) and therefore an additional splitting. Nuclei with *I* = 1 (^2^H, ^14^N) exhibit signals at the frequencies

2 *ν*_±_ = |*A*/2 ± *ν*_n_ ± 3*P*/2|

with *P* being the effective, orientation-selective nuclear quadrupole coupling. The full nqi is be described by the nuclear quadrupole matrix ***P***, which is traceless and symmetric with principal values *P*_*ii*_3
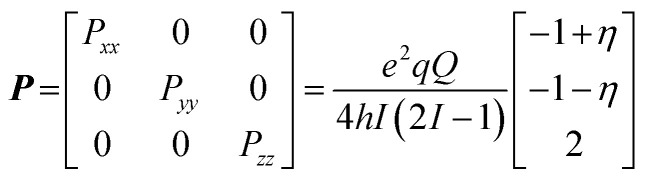


Alternatively, the nqi is expressed by the nuclear quadrupole coupling constant *e*^2^*Qq*/*h* and the asymmetry parameter 
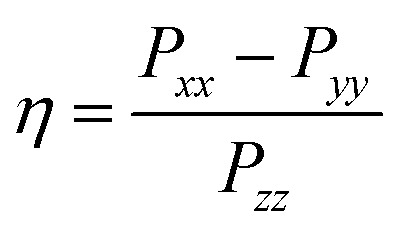
 (|*P*_*xx*_| ≤ |*P*_*yy*_ ≤ |*P*_*zz*_|), which describe the magnitude and the rhombicity of the nuclear quadrupole interaction.^58^

The hyperfine couplings and the Larmor frequencies of isotopes are scaled by their gyromagnetic ratio *γ*, as shown below for ^1^H and ^2^H:4
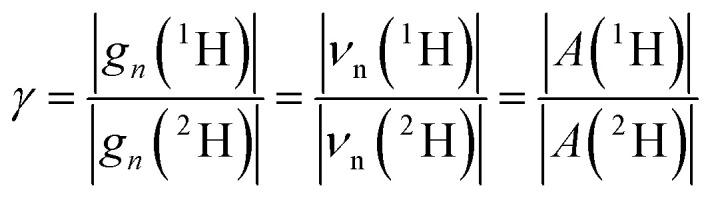


### X-band ESEEM and HYSCORE

ESEEM experiments^59^ were obtained on a Bruker Elexsys-580 and Oxford C-935 liquid helium cryostat with a Bruker ER 4118X-MS3 split ring resonator. Spectra were obtained with a three-pulse ESEEM pulse sequence, π/2 – *τ* – π/2 – Δ*T* – π – Δ*T* – π/2 – *τ* – echo, at 6.5 K and frequencies of 9.48 GHz (pH 6.5), 9.47 GHz (pH 11.5) and 9.54 GHz (pH 12.5), and with the following parameters: π/2 = 8 ns; Δ*T* = 16 ns (pH 6.5, pH 11.5) and 32 ns (pH 12.5); number of points = 1024; repetition rate = 3–8 ms (depending on the sample); shots per point = 5–25 (depending on the sample); number of scans = ∼1–30 (depending on sample and field position). Generally, *τ* values were chosen to suppress ^1^H resonances. They are reported in the captions of the respective figures. HYSCORE^60^ (π/2–*τ*_1_–π/2–Δ*T*_1_–π–Δ*T*_2_–π/2–*τ*_2_–echo) was performed by independently stepping the Δ*T*_1_ and Δ*T*_2_ in 16 ns increments (256 × 256 points), with *τ*_1_ = *τ*_2_ = 360 ns (pH 6.5), 348 ns (pH 11.5) and 350 ns (pH 12.5) and a π/2 = 16 ns microwave pulse length. Spectra were recorded at 9.50 GHz (pH 6.5), 9.48 GHz (pH 11.5) or 9.49 GHz (pH 12.5), respectively, at a temperature of 6.5 K with 5 shots per point and repetition times of 5 ms (pH 6.5 and 12.5) and 7.5 ms (pH 11.5), respectively. A four-step phase cycling was employed to rid the signal of unwanted echoes. Both the ESEEM and HYSCORE data were processed and visualized employing a routine of background subtraction, windowing, zero-filling and fast Fourier transformations. The fitting procedure of the ESEEM is described in the ESI.†

All EPR, ENDOR and ESEEM spectra were processed in Matlab and simulated with the EasySpin (v6.0.0-dev.53 or v6.0.5) package.^61^ Unless otherwise noted, all simulations employed consistent EPR spin Hamiltonian parameters across the various experiments (*i.e.*, ***g***-tensor and Cu hyperfine).

### UV-vis spectroscopy

UV-vis spectra were recorded with an Agilent Cary 60 UV-vis spectrometer in disposable Eppendorf UVettes® (PCR clean purity grade) with 1 cm pathlength between 200 and 1000 nm. *Sm*AA10A concentrations were determined through the measurement of its absorption at 280 nm. The theoretical molar extinction coefficient (*ε* = 35 200 M^−1^ cm^−1^ at this wavelength) was obtained using the Expasy ProtParam tool (https://web.expasy.org/protparam), which was subsequently used to calculate the protein concentration, as described previously.^62^

### DFT calculations

All calculations were performed with the Orca quantum chemistry software (v. 5.0),^63,64^ taking dispersion effects into account by utilizing Grimme's D3 correction with Becke–John damping.^65,66^ Solvation effects (water) were included with the conductor-like polarizable continuum model (CPCM)^67^ and relativistic effects were treated with a zeroth order regular approximation (ZORA).^68–70^ Geometry optimizations and the calculation of EPR parameters were carried out using the B3LYP functional^71,72^ with the all electron def2-TZVP basis set^73,74^ and automatically generated auxiliary basis sets (AutoAux).^75^ Optimized geometry coordinates (*xyz*) are provided in the ESI.†

## Results and discussion

### Absorption spectroscopy and stability studies

**Fig. 1:**
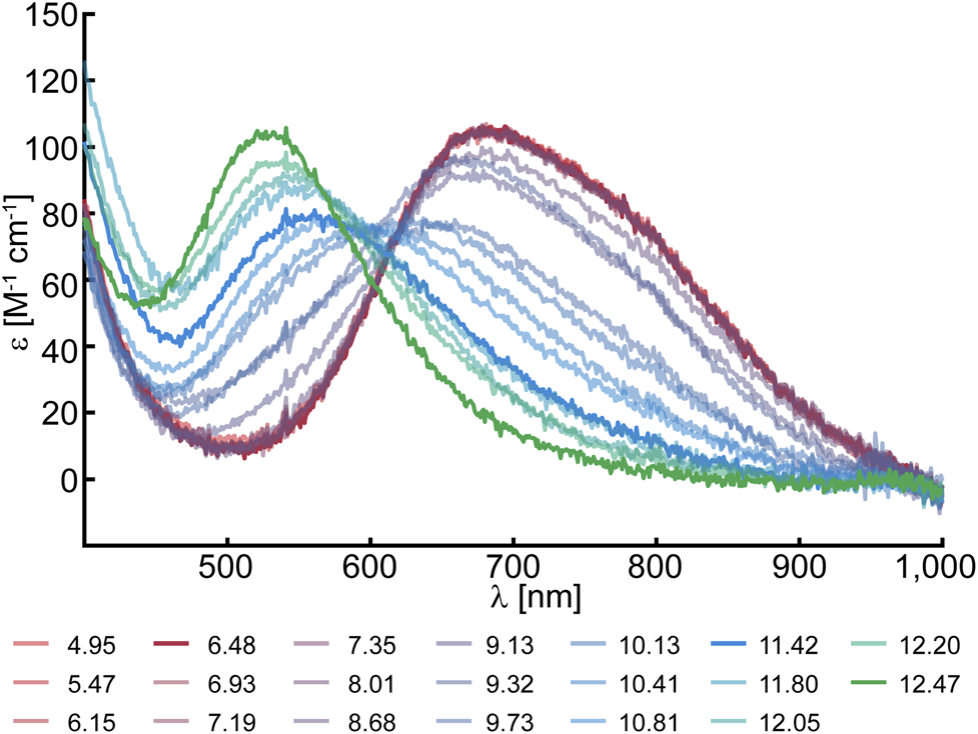
UV-vis spectra of 0.5 mM *Sm*AA10A at several pH values.

Solutions of *Sm*AA10A were characterized between pH 4.0 and pH 12.5, which revealed a prominent pH dependence of the color. At low to neutral pH, protein solutions are blue in color, correlating with a distinct spectral feature in their UV-vis spectra at 678 nm with a molar extinction of 105 M^−1^ cm^−1^ (Fig. 1). This is in good agreement with the spectrum obtained by Munzone *et al.* (675 nm, 120 M^−1^ cm^−1^, pH 6.5).^76^ At higher pH (∼pH 11.5), the blue converts into a pink color, similar to what has been observed for *Bl*AA10.^41^ Accordingly, the d–d band shifts towards higher energy and is found at 555 nm (78 M^−1^ cm^−1^) for *Sm*AA10A at pH 11.5. At even more alkaline conditions (pH 12.5) the transition shifts further to 525 nm (104 M^−1^ cm^−1^).

This blue shift with increasing pH is consistent with the loss of (an) axial water ligand(s) and a change to a distorted square planar geometry,^77–79^ indicative of at least one chemical transition. To assess the number of species developing during the spectrophotometric titration, a singular value decomposition (SVD) was performed. The SVD yielded five significant singular values (Fig. S2 and S3†) corresponding to five distinct (abstract) spectral components. To estimate their respective p*K*_a_ values, the experimental data was reconstructed employing a multi-step acid–base equilibrium (see ESI†). This analysis returned p*K*_a_ values of p*K*_a1_ = 9.65, p*K*_a2_ = 11.97, p*K*_a3_ = 12.02 and p*K*_a4_ = 12.30.

The UV-vis data suggest that *Sm*AA10A is stable under strongly alkaline conditions, which is rather surprising and raised the question whether the functionality of the protein is affected by exposure to extreme pH. It is known that LPMOs are stable and functional in a rather wide pH range,^80^ as shown for *Tt*AA9G (pH 3.0–10.0)^81^ and *Na*AA10A (pH 6.0–10.0).^82^ The lack of a buffer system with sufficient buffer capacity at extremely high pH values precludes activity measurements. To test the potential effects of the alkaline conditions on protein structure and function, *Sm*AA10A was incubated at pH 6.5, 11.5 or 12.5 for 2 minutes or 16 hours and then returned to pH 7.0 for activity testing using β-chitin as a substrate. Thus, we tested for both immediate and long-term enzyme inactivation. The activity data did not show signs of significant enzyme inactivation (Fig. S4†), showing that the active site is not irreversibly damaged at pH 12.5. These findings emphasize the robustness of LPMOs in general, and the histidine brace more specifically, both withstanding pH values far outside of what one typically considers the physiological range, without notable damage.

The stability of the LPMO at extreme pH was also assessed by measuring thermal unfolding of the protein at pH 6.5, 11.5 and 12.5, yielding apparent melting temperatures of 69.9 °C, 51.1 °C and 48.6 °C, respectively (Fig. S5†). In all cases typical unfolding curves were obtained, which, together with apparent melting temperatures well above the temperatures used in this study, show that *Sm*AA10A maintains structural integrity even at pH 12.5.

### EPR spectroscopy

**Fig. 2:**
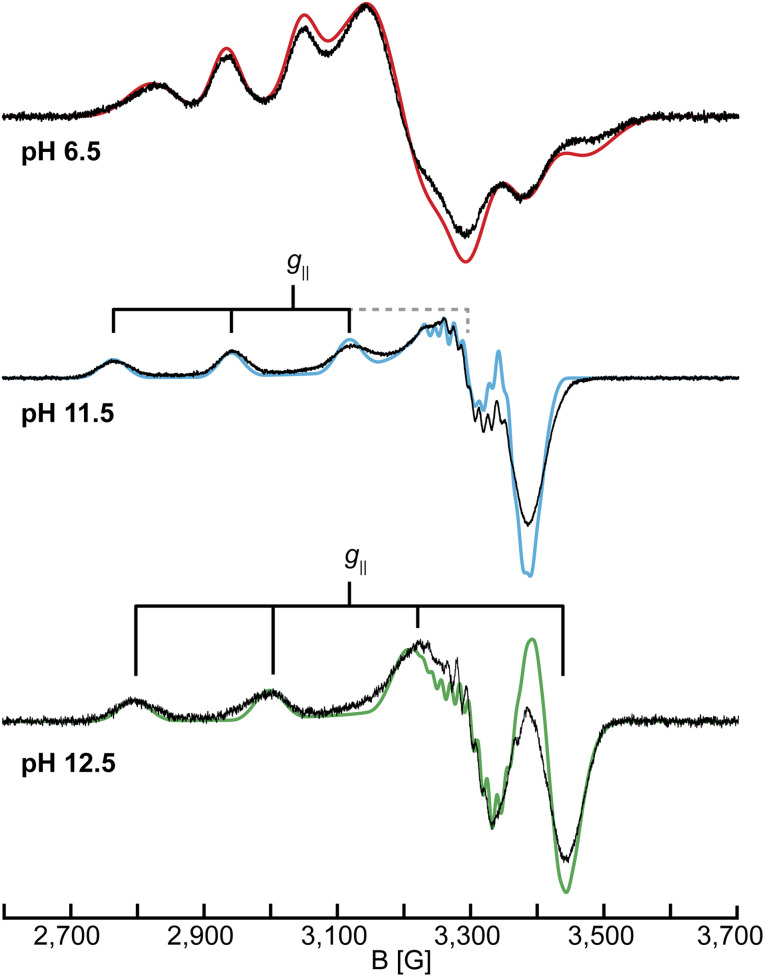
CW X-band (9.46 GHz) EPR spectra of *Sm*AA10A at pH 6.5, 11.5 and 12.5 in black with EPR simulations in color. The simulation for pH 12.5 uses the ^14^N couplings of case A (see section on ^14,15^N ENDOR). EPR parameters are listed in Tables 1 and S1† and experimental conditions are reported in the Experimental section.

To further probe the changes of the Cu(ii) center as a function of pH, continuous-wave (CW) X-band (9.46 GHz) EPR spectra of samples between pH 4.0 and pH 12.5 were collected (Fig. 2 and S6†). For simplicity, we will refer to samples at a given pH as LPMO-*pH* (for example *Sm*AA10A at pH 6.5 is LPMO-6.5).

The spectra in the investigated pH range show a total of four species, as obtained by simulation. At low to neutral pH (pH ≤ 6.5), a single copper species is observed by EPR and reproduced by simulation with a rhombic ***g***-tensor and large line broadening (Table 1). The EPR parameters applied (***g*** = [2.258, 2.096, 2.023], ***A*** = [350, 100, 240] MHz) are in excellent agreement with the ones determined by Munzone *et al.* (***g*** = [2.258, 2.114, 2.024], ***A*** = [375, 57, 268] MHz) at pH 6.5.^76^ At more alkaline conditions (pH 11.5) an axial ***g***-tensor and resolved nitrogen superhyperfine splitting are observed and the spectrum is also well reproduced by simulation of single copper species with three strongly coupled nitrogen nuclei. This is in excellent agreement with observations made for other AA10 LPMOs (*Pl*AA10 (ref. 42) and *Bl*AA10 (ref. 41)). DFT computation of the ***g***-tensor and simulations of the ^14^N superhyperfine splitting pattern for *Pl*AA10 suggest that the change in the ***g***-tensor is the result of a change in the coordination sphere.^42^ At lower pH, two waters coordinate the copper, creating a bipyramidal (3N2O) coordination environment and a rhombic EPR spectrum, while at higher pH, the coordination of a single hydroxyl ion and an intact histidine brace coordination with distorted square-planar symmetry (3N1O) yields the axial EPR spectrum.^42^ A more detailed description of the quantification of each individual species at intermediate pH values of our titration (between pH ∼9 and pH ∼12) and the chemical processes associated with these is given in the ESI (Fig. S6, Tables S1 and S2†).

**Table 1:** Summary of the X-band EPR parameters for the three species investigated in this study. More detailed simulation parameters are provided in Table S1, whereas Table S2 shows the composition of species at a multitude of pH values between pH 4.0 and pH 12.5

	LPMO-6.5	LPMO-11.5	LPMO-12.5
***g*** = [*g*_1_, *g*_2_, *g*_3_]	[2.258, 2.096, 2.023]	[2.230, 2.051, 2.042]	[2.179, 2.047, 2.030]
***A*** (^63^Cu) = [*A*_1_, *A*_2_, *A*_3_] (MHz)	[350, 100, 240]	[538, 25, 99]	[605, 85, 70]

Above pH 11.5, additional species exhibiting axial EPR spectra are observed (see also Q-band EPR in the ESI, Fig. S7 and Table S3†), yielding a total of five species between pH 4.0 and pH 12.5, in agreement with the results from the UV-vis SVD analysis. The species show a general decrease of *g*_‖_ and an increase in *A*_‖_ (Table 1 and S3†) to ultimately reach *g*_‖_ = 2.179 and *A*_‖_ = 605 MHz for the dominant species in LPMO-12.5. This trend is tentatively attributed to a reduction of the overall net charge of the copper coordination site, potentially resulting from the deprotonation of the coordinating ligands at increased pH values.^83^ In the pH titration of *Bl*AA10, an additional species with EPR parameters of *g*_‖_ = 2.180 and *A*_‖_ = 614 MHz was observed, similar to the parameters obtained for the dominant species in LPMO-12.5.^41^ As mentioned above, the authors hypothesized that this species could be connected to a deprotonation of the primary amine, however, no further characterization of this species was reported. The spectrum of LPMO-12.5 is well reproduced by simulation with the inclusion of three strongly coupled nitrogen nuclei, confirming that the histidine brace is intact and coordinated to the copper center. However, the assignment of the nitrogen hyperfine couplings to the individual ligands is not feasible from the EPR alone. Therefore, we employed ^14,15^N ENDOR spectroscopy (*vide infra*), which helped to fully resolve the nitrogen hyperfine tensors for LPMO-6.5 and LPMO-11.5. For LPMO-12.5 two distinct cases (case A and case B) yield satisfactory EPR and ENDOR simulations, which will be described and discussed in detail in the ^14,15^N ENDOR section.

### ^1,2^H ENDOR

To obtain more information on the nature of the deprotonation of the *Sm*AA10A active site, specifically the character of the exchangeable protons, the protein was further characterized by ^1^H and ^2^H ENDOR spectroscopies. For that purpose, samples in H_2_O and D_2_O at pH 6.5, 11.5 and 12.5 were prepared.

The ^1^H Davies spectra of all three samples in H_2_O show a variety of signal pairs centered at the nuclear Larmor frequency (*ν*_n_ ∼51 MHz at 12 kG) split by their effective hyperfine couplings (*A*), as expected for weakly coupled nuclei (eqn (1); Fig. S8†). These broad and rather complex spectra with splittings corresponding to hyperfine couplings of up to 13 MHz are the result of the multiple protons in the histidine brace, all with varying hyperfine couplings and orientations.

**Fig. 3:**
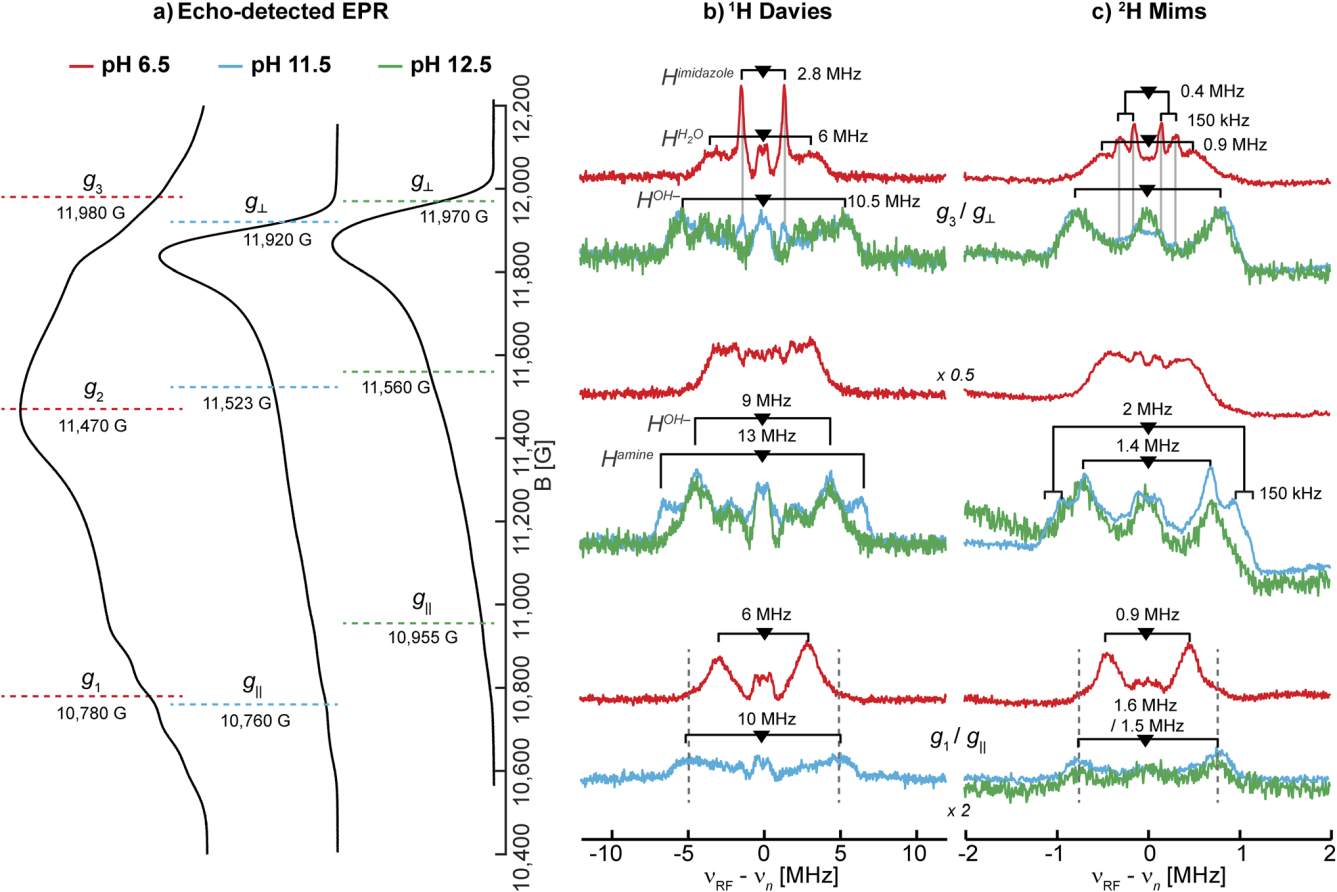
Larmor centered ^1^H and ^2^H ENDOR spectra depicting the exchangeable protons in *Sm*AA10A. (a) Q-band echo detected EPR spectra with selected magnetic field position marked (colored dashed lines) corresponding to magnetic field positions of the ^1^H and ^2^H ENDOR experiments in (b) and (c), respectively. (b) ^1^H Davies difference spectra (obtained through subtraction of spectra collected for samples in H_2_O and D_2_O) and (c) ^2^H Mims spectra at three field positions at pH 6.5 (red), 11.5 (blue) and 12.5 (green). The larger couplings associated with the amine protons, apparent in LPMO-11.5, are marked with dashed lines to emphasize the similarities to LPMO-6.5. The imidazole protons in LPMO-6.5 and LPMO-11.5 are traced with solid, light grey lines. The underlying spectra are shown in Fig. S8.† The low field position of the ^1^H Davies difference spectrum recorded at pH 12.5 exhibits low S/N and is for clarity shown in Fig. S9.† Experimental conditions are reported in the Experimental section.

^1^H ENDOR difference spectra of samples prepared in H_2_O and D_2_O reveal the ^1^H ENDOR signals arising only from the exchangeable protons of the aquo/hydroxo ligands, the primary amine, and the remote nitrogen nuclei of the histidine imidazole groups (Fig. 3 and S8†). For LPMO-6.5 one broad set of signals with a hyperfine coupling of 6 MHz along *g*_1_ and *g*_3_, as well as a sharp signal pair along *g*_3_ with a splitting of 2.8 MHz are observed (Table 2). The 6 MHz doublet can tentatively be assigned to the protons of coordinated water(s). This is within the typical range of axial water couplings as observed for Cu(ii) amyloid-β peptide^84^ and the prion protein^85^ (each 3–4 MHz) on one side and [Cu(H_2_O)_6_]^2+^ in Mg(NH_4_)_2_(SO_4_)_2_·6H_2_O (8 MHz) on the other side.^86^ While there are other ENDOR doublets observed in the ^1^H difference ENDOR spectrum of LPMO-6.5, for instance the 2.8 MHz coupling at *g*_1_, assignments of these features become clear when comparing samples of different pHs.

**Table 2:** Summary of the observed ^1^H hyperfine couplings (*A*) for the exchangeable protons in *Sm*AA10A at pH 6.5, 11.5 and 12.5 shown in Fig. 3. Values are reported in MHz

	pH 6.5	pH 11.5	pH 12.5
H_*x*_O	*A*_3_: 6	*A*_⊥_: 10.5	*A*_⊥_: 10.5
	*A*_1_: 6	*A*_mid_: 9^a^	*A*_mid_: 9^a^
		*A*_‖_: 10	*A*_‖_: 10
Amine-H	*A*_1_: 10 (broad)	*A*_⊥_: 10.5	—
		*A*_mid_: 13^a^	
		*A*_‖_: 10	
Imidazole-H	*A*_3_: 2.8	*A*_⊥_: 2.8	—

a Measured approximately midway between the *g*_⊥_ and *g*_‖_, as shown in Fig. 3.

The ^1^H ENDOR of the exchangeable protons of LPMO-11.5 shows a broad set of ^1^H ENDOR doublets with a significantly larger hyperfine coupling compared to LPMO-6.5. At both edges of the field-frequency pattern of LPMO-11.5, couplings of approximately 10 MHz are observed. However, at the intermediate field position, two doublets are resolved with couplings of 9 MHz and 13 MHz. For LPMO-12.5 the ^1^H difference ENDOR pattern at the edges is similar to LPMO-11.5 but the intermediate field position exhibits only the 9 MHz doublet and not the larger coupling of 13 MHz seen for LPMO-11.5 (Fig. 3b).

Based on the EPR and ENDOR spectroscopy of *Sm*AA10A above, it is expected that two water molecules or one hydroxo ligand complete the coordination sphere of the copper center under neutral or alkaline conditions, respectively. At higher pH conditions between LPMO-11.5 and LPMO-12.5, we assume that the hyperfine character of the hydroxo's exchangeable proton remains relatively constant, allowing to draw some conclusions about the ^1^H difference ENDOR presented in Fig. 3b. For LPMO-11.5 and LPMO-12.5, a common exchangeable ^1^H signal of *A* ∼9 MHz at the intermediate field position is observed, and assigned to the coordinated hydroxo ligand. The spectral difference between the two samples with an *A* ∼13 MHz coupling is then attributed to changes (deprotonations) of the histidine brace. This value is in the expected range of Cu-coordinating amine protons, as reported for [Cu(NH_3_)_4_]^2+^ (*A*_‖_: −12 MHz, *A*_⊥_: −12 MHz)^15^ and the Cu_B_ site of pMMO (*A*_‖_: −13 MHz, *A*_⊥_: −12 MHz)^15^ or the coordinating amine function of an N-terminal aspartic acid in the amyloid-β peptide (***A*** = [15.0, −10.5, −10.5] MHz).^84^ The absence of this coupling in LPMO-12.5 indicates the deprotonation (loss of an exchangeable proton) of the amine function in *Sm*AA10A at pH values above pH 11.5.

In Cu-bis-(l)-histidine two sets of exchangeable amine protons with differing couplings along *g*_2_ were identified (*A*(^1^H^amine-1^): [6, 7, 14] MHz, *A*(^1^H^amine-2^): [6, 10, 14] MHz),^87^ which supports the potentially inequivalent character of the two amine protons in *Sm*AA10A. A smaller hyperfine coupling for the second amine proton would lead to an overlap with the hydroxo protons, explaining why it is not readily identified and why the difference spectra at the edges of the ENDOR spectrum appear to originate from a single exchangeable proton. Despite equivalent Cu–(N)–H distances, the two protons of the amine may demonstrate different hyperfine couplings originating from a significant variance in their isotropic hyperfine coupling, which is a function of the N(imidazole)–Cu–N(amine)–H dihedral angle. This effect was demonstrated by Peisach and co-workers for several copper amino acid complexes^88^ having a hyperconjugation-like relationship.^89^ Ultimately, the loss of the one resolved exchangeable proton at pH 12.5, observed best at the intermediate field, signifies the deprotonation of the N-terminal amine.

**Scheme 2:**
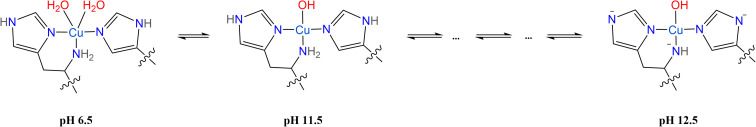
Proposed species of *Sm*AA10A at pH 6.5, 11.5 and 12.5 characterized by ^1,2^H ENDOR spectroscopy.

Upon closer inspection of the ^1^H difference spectra of LPMO-11.5 along *g*_⊥_, an additional sharp ENDOR doublet signal equivalent to a fairly weak coupling of ∼2.8 MHz is visible, similar to what is seen for LPMO-6.5 (Fig. 3b and S8†). While for LPMO-6.5 these couplings may be partially the result of an axially coordinated water, potentially contributing to the overall intensity of this feature, this axial water is likely absent LPMO-11.5. Instead, we attribute these features to the protons of the remote nitrogen nuclei of the imidazole rings, similar to what has been observed for the equivalent protons in Cu-bis-(l)-histidine.^87^ Strikingly, for LPMO-12.5 these couplings are absent, hinting at a deprotonation not just at the amine function (to the azanido), but also occurring at the remote nitrogen nuclei of the two imidazole moieties (to imidazolates; Scheme 2).

Naturally, at neutral pH, the amine should also present an exchangeable proton signal. On closer examination, the spectra of LPMO-6.5 exhibit wings along *g*_1_ with couplings of approximately 10 to 12 MHz (Fig. 3b) that can tentatively be assigned to a similar exchangeable proton of the amine function as we have now shown for LPMO-11.5. Due to the larger number of exchangeable water protons, these signals still dominate the spectrum. The larger linewidth observed in the ^1^H ENDOR spectra of LPMO-6.5 compared to LPMO-11.5 and LPMO-12.5 is likely due to the larger distribution of orientations and coupling strengths caused by the disorder of the coordinated waters.

To further validate the ^1^H ENDOR difference spectra, the resonances of the ^2^H nuclei were directly measured in ^2^H Mims experiments (Fig. 3c). The spectra are in very good agreement with the ^1^H Davies, exhibiting hyperfine couplings that are equivalent to the determined ^1^H couplings when scaled by the gyromagnetic ratios of the two nuclei (*γ*(^1^H)/*γ*(^2^H) ≈ 6.5; Fig. S10†). For deuterium (*I*(^2^H) = 1) an additional quadrupole interaction occurs, splitting the hyperfine doublet further (eqn (2)). The small ^2^H quadrupole splittings are challenging to observe due to the broader ENDOR linewidth, hence the rare examples of resolved ^2^H quadrupole splittings in metalloproteins.^90,91^ Surprisingly, we do observe clear additional quadrupole splittings in LPMO-6.5 along *g*_3_ and in LPMO-11.5 at intermediate field positions (Fig. 3c). The splittings of approximately 3*P* = 150 kHz (*e*^2^*qQ*/*h* = 100 kHz) are in a typical range for nuclear quadrupole interactions of deuterium, similar to what has been found for the deuterium nuclei in Cu(ii)-bis(glycinato) (*e*^2^*qQ*/*h* = 188 kHz), where the maximum quadrupole splitting is oriented along the ^2^H–N bond.^91^ For LPMO-6.5 and LPMO-11.5, we have tentatively assigned the weaker ^1^H couplings of 2.8 MHz along *g*_⊥_ (*A*(^2^H) ∼0.4 MHz) to the exchangeable protons of the imidazoles' remote nitrogen (Fig. 3b). While the quadrupole splitting in LPMO-6.5 is very pronounced, it is less apparent, but still observable in LPMO-11.5 (Fig. 3c). This similarity confirms that the signals arise from a similar origin. Furthermore, it suggests that the imidazole rings lie in the plane of *g*_⊥_ for both samples. The maximum quadrupole splitting for the amine deuteron is observed at an intermediate field of the field-frequency pattern between *g*_⊥_ and *g*_‖_. This observation relates to the orientation of the ^2^H–N bond which is angled between the *g*_⊥_ plane (*x*,*y*) and the *g*_‖_ (*z*) directions.

### ^14^N ESEEM and ^15^N HYSCORE

**Fig. 4:**
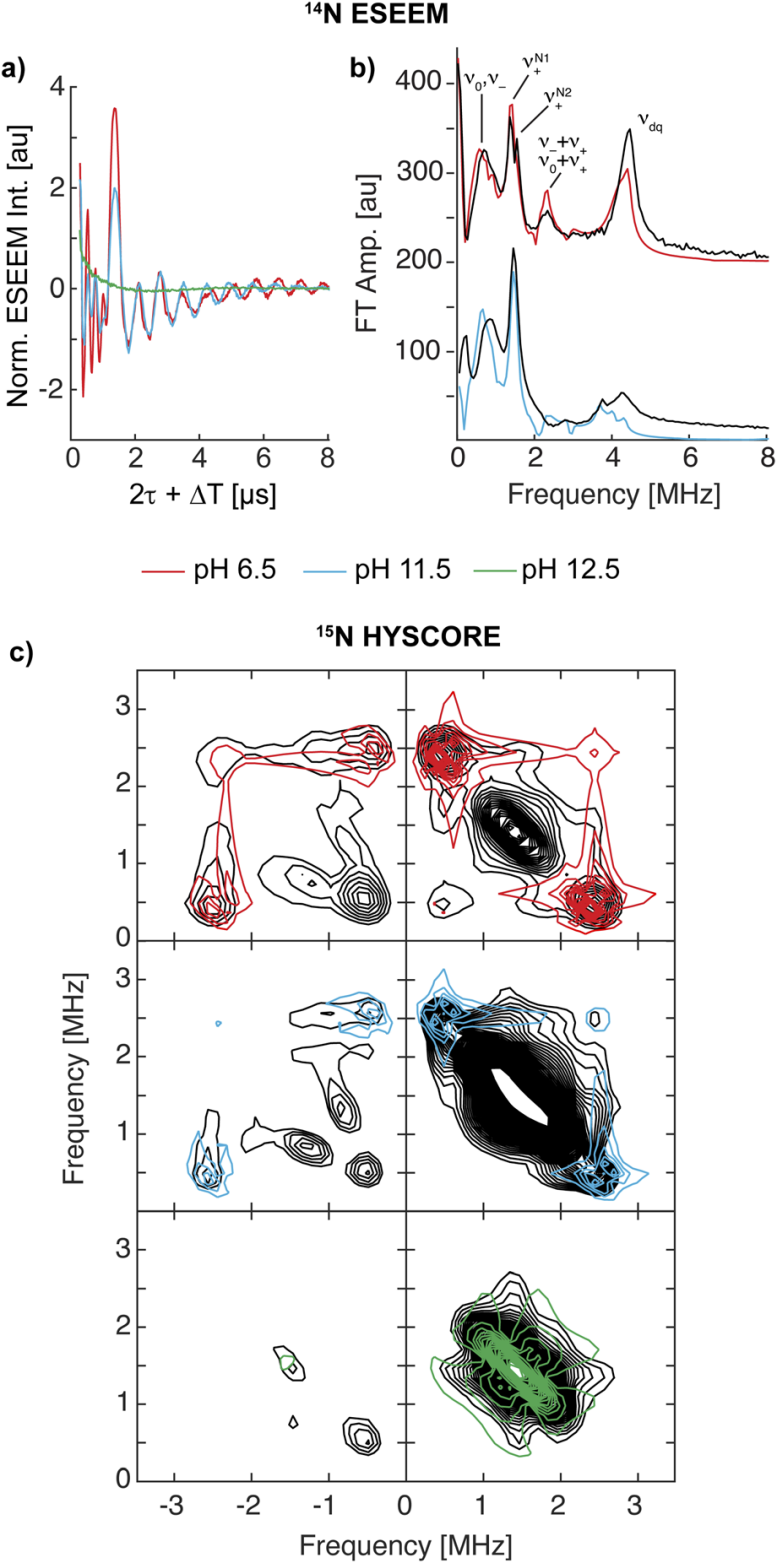
(a) X-band three-pulse ESEEM spectra, normalized by their echo intensity recorded for *Sm*AA10A at pH 6.5 (red, 3249 G, 144 ns *τ*), pH 11.5 (blue, 3340 G, 140 ns *τ*) and pH 12.5 (green, 3337 G, 140 ns *τ*). (b) The FT spectra and best fits of LPMO-6.5 (*τ* = 136 ns, 3431 G) and LPMO-11.5 (*τ* = 140 ns, 3340 G) where the prominent quadrupole transitions (*ν*_+/0/−_) and the double quantum (Δ*m*_*I*_ = 2, *ν*_dq_) are labeled. The *τ* values were chosen to suppress the ^1^H response as best as possible. Additional magnetic field positions were collected and time-domain fits were performed by a multi-field global simulation routine (Fig. S13 and S16†). (c) X-band ^15^N HYSCORE spectra of ^15^N-*Sm*AA10A (black) and respective simulations in color at pH 6.5 (red, 3.226 G), pH 11.5 (blue, 3.327 G) and pH 12.5 (green, 3.300 G). ^15^N simulation parameters for LPMO-6.5 and LPMO-11.5 employed hyperfine values from best ^14^N ESEEM fits, scaled by the gyromagnetic ratio of the two isotopes (*γ*(^15^N/^14^N) ∼1.4). LPMO-12.5 is simulated with a weak coupling of ***A***(^15^N) = [−0.3, −0.3, 0.9] MHz.

The EPR and ^1,2^H ENDOR experiments thus far indicate significant changes in the histidine brace as the pH is increased, potentially due to deprotonation of the amine function and the remote nitrogen nuclei of the coordinated imidazole moieties. To further study the two imidazole rings, specifically the properties of the remote nitrogen nuclei, three-pulse X-band ESEEM experiments were conducted, which aid to resolve not just the remote nitrogen's hyperfine, but also their nuclear quadrupole interaction (nqi), as well demonstrated for various copper-imidazole active-sites.^92–96^ The time-domain spectra of LPMO-6.5 and LPMO-11.5 show comparable modulation patterns and intensities (Fig. 4a), which indicates very similar hyperfine and quadrupole interactions for both of the remote nitrogen nuclei. This result also confirms that the number of equivalent nitrogen nuclei (two) remains constant between LPMO-6.5 and LPMO-11.5, in accord with the results and considerations described above.

Fitting of the ESEEM spectra of LPMO-6.5 and LPMO-11.5 (Fig. 4b, S13–S17, Tables S4 and S5†) reveals nuclear quadrupole and hyperfine couplings which fall in the range of other remote nitrogen nuclei in imidazole rings.^92–96^ The individual quadrupole tensor of each imidazole's remote nitrogen nucleus is well estimated, where slight differences in the quadrupole couplings of the two histidine ligands (*e*^2^*qQ*/*h* = 1.359 and 1.498 MHz; *Δ* = 0.139 MHz) are observed for LPMO-6.5, however, these ligands appear more similar in LPMO-11.5 (*e*^2^*qQ*/*h* 1.374 and 1.438 MHz; *Δ* = 0.064 MHz). A more detailed discussion of the fitting models and parameters, with comparison to those recently obtained from a HYSCORE spectrum reported by Munzone *et al.*,^76^ is given in the ESI.†

For LPMO-12.5, a complete loss of ^14^N modulation in the three-pulse ESEEM experiment is observed (Fig. 4a). This could initially lead to the interpretation of lacking imidazole coordination, contradicting both the EPR and ^1,2^H ENDOR analysis above and our ^14,15^N ENDOR studies (*vide infra*). To understand the lack of ^14^N ESEEM signal, we collected X-band HYSCORE spectra of globally ^15^N-enriched samples (Fig. 4c). The ^15^N HYSCORE spectra of ^15^N-LPMO-6.5 and ^15^N-LPMO-11.5 show a set of cross coupling peaks at (−0.5, +2.5) and (−2.5, +0.5) in the ‘strong coupling’ (−, +) quadrant, and a corresponding set of signals in the ‘weak coupling’ (+, +) quadrant ((+0.5, +2.5) and (+2.5, +0.5)), indicating that the underlying transition is near the cancellation regime (|*a*_iso_| ∼2|*ν*_N_|). Employing the hyperfine tensors obtained from the ESSEM fitting, scaled by the gyromagnetic ratio of both nuclei (γ(^15^N/^14^N) ∼1.4), we obtained simulations that reproduce the observed HYSCORE pattern of the remote nitrogens well (Fig. 4c and S18†). For LPMO-12.5, a dramatically different HYSCORE response is observed, where no strongly coupled signals are detected in the (−, +) quadrant. This indicates, that the *a*_iso_ values of the imidazoles' remote nitrogen nuclei are significantly reduced upon deprotonation. This is in agreement with the lack of modulation observed in the ESEEM spectra.

For all three samples an additional broad signal on the off diagonals of the ^15^N Larmor frequency in the (+, +) quadrant is observed, which has been reported for *Sm*AA10A before and has been attributed to other distant amino acids.^76^ The feature is well reproduced by a generally dipolar ^15^N hyperfine tensor of ***A*** = [−0.3, −0.3, 0.9] MHz (Fig. 4c).

The substantially decreased hyperfine coupling of the imidazoles' remote nitrogen nuclei upon deprotonation is associated with a decreased nitrogen s orbital contribution to the SOMO, most likely concomitant with increased local p orbital contribution *via* additional π interaction at the nitrogen. We further conducted DFT hyperfine calculations of [Cu(imidH)_4_]^2+^ and [Cu(imid)_4_]^2−^, which predict isotropic hyperfine couplings of 2.57 and 0.06 MHz for their remote nitrogen nuclei, respectively. The Mulliken spin populations, however, increase from 1.6 × 10^−3^ to 7.4 × 10^−3^. This shows that the *a*_iso_ values of remote nitrogen nuclei in imidazoles are drastically reduced upon deprotonation, despite an overall increase in spin density, in agreement with our observations made in the ESEEM and HYSCORE experiments (See ESI† for more discussion).

### ^14,15^N ENDOR

The ^1,2^H ENDOR, ^14^N ESEEM and ^15^N HYSCORE experiments suggest that the remote nitrogen nuclei of the imidazole rings and the amine function are deprotonated at pH > 11.5. Such deprotonations would lead to significant changes of the electronic and magnetic properties of the coordinating nitrogen nuclei and can be monitored *via* their hyperfine and nuclear quadrupole interactions. For LPMO-6.5, the large EPR linewidth in the CW X-band EPR spectrum does not allow us to accurately extract any information on the coordinating nitrogen nuclei (Fig. 2), whereas, the nitrogen superhyperfine splitting in the X-band EPR spectra of *Sm*AA10A at pH 11.5 and 12.5 can be partly resolved. However, the multitude of nitrogen nuclei and the axial nature of the ***g***-tensor prevent conclusive analyses and definitive assignments of the individual hyperfine and quadrupole couplings to specific ligands from the EPR spectrum alone. Hence, we employed nitrogen ENDOR spectroscopy for a more detailed survey of the three coordinating nitrogen nuclei of the histidine brace.

**Fig. 5:**
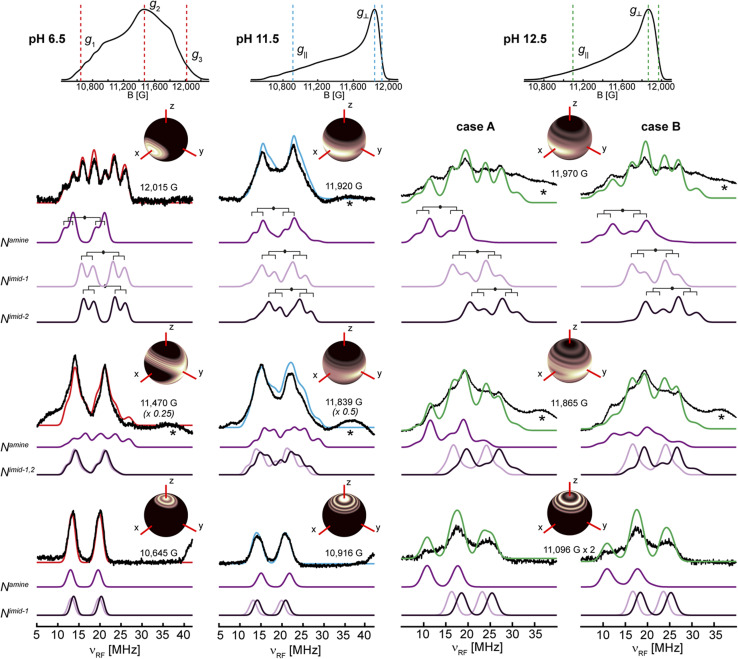
^14^N Davies ENDOR spectra of *Sm*AA10A at pH 6.5, 11.5 and 12.5 in black with simulations of the three individual nitrogen nuclei in shades of purple and the total (summed) simulation in red (pH 6.5), blue (pH 11.5) and green (pH 12.5). The spectra are shown at three field positions, which are indicated in the echo-detected EPR spectra at the top. For pH 12.5, simulations of the two discussed cases are presented (case A: ***A***(N^amine^) = [27.5, 37.0, 27.0] MHz and case B: ***A***(N^amine^) = [27.0, 45.0, 28.0] MHz). Insets in the figures show the orientations that are excited at the respective field positions (black to white gradient: no orientation selection to maximum selection), stressing the higher orientation selection for LPMO-6.5 compared to LPMO-11.5 and LPMO-12.5; these insets were created with *orisel* in EasySpin (plotting without ^14^N hyperfine interaction). Simulation parameters are listed in Table 1 (***g***-tensors and copper hyperfine) and Table 3 (nitrogen hyperfine and quadrupole). The full ENDOR pattern and the spectra of ^15^N-enriched samples are depicted in Fig. S19.† Spectrometer conditions are reported in the Experimental section. Asterisks (*) indicate background resonances, likely originating from copper hyperfine (see ESI†).

To refine the hyperfine assignments and analysis, we collected ENDOR spectra on samples of natural abundance (Fig. 5) and on the globally ^15^N-enriched samples (Fig. S19†). As anticipated for strongly coupled nuclei (eqn (1)), the ENDOR doublets are centered at half of the hyperfine coupling and split by twice the Larmor frequency (*ν*_n_ ∼ 5 MHz (^15^N) and ∼4 MHz (^14^N) at 12 kG). For samples of natural abundance (^14^N: 99.6%, ^15^N: 0.4%) the additional nqi of the ^14^N (*I* = 1) isotope results in a further splitting (eqn (2)) of signals, resulting in the generation of doublets of doublets. The accurate determination of the nqi yields information about its relative orientation to the ***g***-tensor, providing excellent insight into the bonding structure. The ^15^N (*I* = 1/2) enriched samples lack nqi, and further refined and reinforced the hyperfine estimations for the ^14^N ENDOR analysis. Spectra for samples of natural abundance and ^15^N labelled samples were simulated with common hyperfine tensors (scaled by the gyromagnetic ratio *γ*(^15^N)/*γ*(^14^N) ≈ 1.4) (Fig. S19†).

For all three samples, the ENDOR spectra at low magnetic field (*g*_1_/*g*_‖_) are especially interesting, as they probe a “single crystal-like” position of the field-frequency pattern, therefore approximately only one direction (*z*) of the molecular frame.^97,98^ The orientation selection plots (Fig. 5 insets) mark the excited orientations at each magnetic field position. These spheres depict the distinct excitation of the molecular orientations at the three principal *g*-values for LPMO-6.5, where single molecular orientations are probed at *g*_1_ (*z*) and *g*_3_ (*x*). In all cases, copper hyperfine contributes significantly to the observed orientation selection, broadening the observed selection bands in the spheres. The single ENDOR doublet (*ν*_±_) detected for LPMO-6.5 and ^15^N-LPMO-6.5 at this field position (Fig. 5 and S19†) indicates that all three directly coordinating nitrogen nuclei have similar hyperfine couplings along *g*_1_ with no/minimal quadrupole splitting. At higher field position this doublet evolves into two sets of signals with pronounced quadrupole splitting for the ^14^N nucleus. The full field-frequency pattern of LPMO-6.5 (Fig. 5 and S19†) and ^15^N-LPMO-6.5 (Fig. S19†) can be simulated under consideration of three strongly coupled nitrogen nuclei with mostly isotropic hyperfine couplings, comparable to one another in size and anisotropy (Table 3). Most importantly, the largest principal values of the hyperfine tensors, *A*_max_, vary in their direction, with two tensors orienting *A*_max_‖*g*_3_ and the third tensor orienting *A*_max_‖*g*_2_. Following the assumption that *A*_max_ is aligned with the Cu–N bond direction, we assign the three tensors to the two *trans*-positioned imidazole nitrogen nuclei (N^imid^-1 and N^imid^-2) and the amine nitrogen (N^amine^), respectively. This assignment is further supported by the quadrupole interactions of the ^14^N nuclei, with the largest value, *P*_max_, again oriented along the respective assigned Cu–N bonds. The smallest components are oriented along *g*_1_, approximately normal to the imidazole plane, as previously reported for other copper coordinated imidazole rings.^99,100^

**Table 3:** Hyperfine and nuclear quadrupole parameters for LPMO-6.5, LPMO-11.5 and LPMO-12.5 (case A and case B) obtained through simulation of the spectra depicted in Fig. 5 and S19 with three strongly coupled nitrogen nuclei N^amine^, N^imid^-1 and N^imid^-2. All parameters are reported for the ^14^N isotope

	***A*** = [*A*_1_, *A*_2_, *A*_3_] [MHz]	*a*_iso_ a [MHz]	*ρ*_total_ b [%]	***P*** = [*P*_1_, *P*_2_, *P*_3_] [MHz]	*e* ^2^ *Qq*/*h*^c^ [MHz]	*η*
**LPMO-6.5**						
N^amine^	[32.0, 44.9, 32.2]	36.4	25.3	[0.3, −1.1, 0.8]	−2.2	0.45
N^imid^-1	[32.8, 32.5, 42.6]	36.0		[0.3, 0.7, −1.0]	−2.0	0.4
N^imid^-2	[33.9, 33.0, 43.2]	36.7		[0.2, 0.7, −0.9]	−1.8	0.56

**LPMO-11.5**						
N^amine^	[35.5, 48.0, 35.5]	39.7	26.1	[0.3, −1.1, 0.8]	−2.2	0.45
N^imid^-1	[32.0, 32.0, 42.0]	35.3		[0.3, 0.7, −1.0]	−2.0	0.4
N^imid^-2	[34.0, 34.0, 45.0]	37.7		[0.2, 0.7, −0.9]	−1.8	0.56

**LPMO-12.5 (case A)**						
N^amine^	[27.5, 37.0, 27.0]	30.5	21.7	[0.4, −1.7, 1.3]	−3.4	0.52
N^imid^-1	[39.0, 39.0, 44.5]	40.8		[0.3, 1.1, −1.4]	−2.8	0.57
N^imid^-2	[43.0, 43.0, 53.0]	46.3		[0.3, 0.9, −1.2]	−2.4	0.5

**LPMO-12.5 (case B)**						
N^amine^	[27.0, 45.0, 28.0]	33.3	25.4	[0.3, −1.5, 1.2]	−3.0	0.6
N^imid^-1	[40.0, 39.0, 44.0]	41.0		[0.3, 1.0, −1.3]	−2.6	0.53
N^imid^-2	[43.0, 42.0, 52.0]	45.6		[0.2, 1.3, −1.5]	−3.0	0.73

a The isotropic hyperfine contribution (*a*_iso_) is calculated as follows: *a*_iso_ = (*A*_1_ + *A*_2_ + *A*_3_)/3.

b The total spin population *ρ*_total_ of the histidine brace is calculated by estimating the s and p orbital spin populations from the isotropic (*a*_*iso*_) and anisotropic (*t*) contribution of the hyperfine coupling (see ESI) for each individual nitrogen nucleus. Both contributions are then added and summed for all three nuclei. The individual contributions are listed in Table S6.

c *e* ^*2*^ *qQ*/*h* estimated from eqn (3), using only *P*_max_.

The field-frequency patterns of LPMO-11.5 and ^15^N-LPMO-11.5 (Fig. 5 and S19†) exhibit significantly different patterns compared to the sample at pH 6.5. The most dramatic difference is at high field where the individual features are less well-separated for LPMO-11.5, whose nearly axial EPR spectrum causes a mixture of *g*_2_ and *g*_3_ to be detected. This is best seen in its orientation selection plots (Fig. 5 insets), exhibiting significantly less selectivity compared to LPMO-6.5. However, despite the very small rhombic splitting of the ***g***-tensor and the Cu hyperfine contribution, some orientation preferences at the two highest field positions are retained, selecting either more *y* at the maximum of the EPR spectrum or more *x* at the very high edge of the EPR spectrum.

The ENDOR pattern of LPMO-11.5 is well reproduced by using the same nuclear quadrupole interactions and a comparable trio of hyperfine tensors as used for the simulation of LPMO-6.5 (Table 3). The fairly equivalent hyperfine and quadrupole parameters of LPMO-6.5 and LPMO-11.5 underpin the negligible differences in the histidine brace as the pH is increased from pH 6.5 to pH 11.5, a conclusion that was also drawn from our studies of the remote imidazole nitrogen nuclei, described above. Perhaps this is somewhat unsurprising as the exchange of coordinating aquo ligands for a hydroxide, proposed for this transition, is not expected to significantly perturb the nature of the histidine brace. This first application of orientation-selective ENDOR spectroscopy to LPMOs has now experimentally differentiated the hyperfine (and quadrupole) tensors of the three coordinating nitrogens, allowing for unambiguous assignment of distinct ligand interactions, something that is not available directly from the EPR spectrum.

**Fig. 6:**
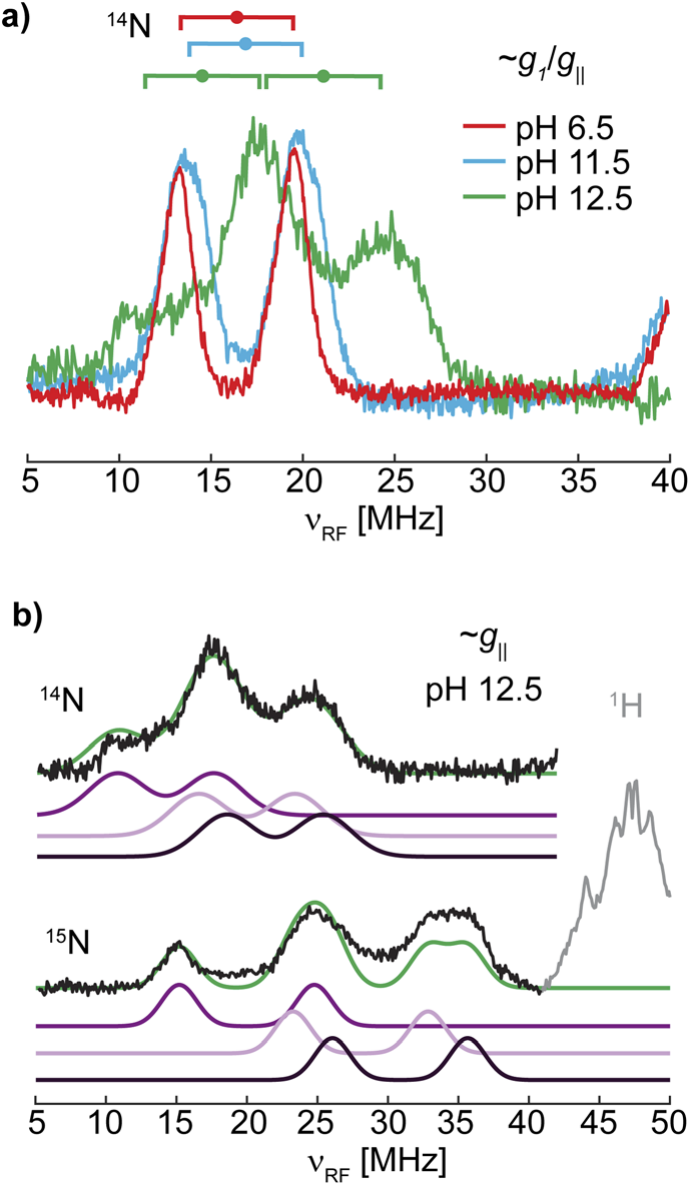
(a) Overlay of the ^14^N ENDOR spectra of LPMO-6.5, LPMO-11.5, and LPMO-12.5 collected along *g*_1_/*g*_‖_ (10.900, 10.916, 11.096 G, respectively; also shown in Fig. 5 and S19†) with goalposts centered at *A*/2 and split by 2*ν*_n_. (b) Gaussian doublet fits of the ^14^N and ^15^N ENDOR with individual nitrogen doublet pairs and their sum (green) for (^15^N)-LPMO-12.5.

The ^14^N ENDOR spectrum of LPMO-12.5 collected along *g*_‖_ is distinctly different from the spectra of LPMO-6.5 and LPMO-11.5 (Fig. 5 and 6). Instead of a single doublet, three features are observed that are best modelled as the sum of three equally intense Gaussian doublets (centered at *A*/2, split by 2*ν*_n_) (Fig. 6b). A single doublet with a smaller hyperfine coupling *A*(^14^N) of ∼28.5 MHz is assigned to N^amine^, and the two larger and more similar couplings of 40 and 44 MHz are assigned to N^imid^-1 and N^imid^-2. Still, no significant quadrupole splitting is detected. The ^15^N ENDOR response can be modelled analogously (Fig. 6b), resolving the two *ν*_+_ features of N^imid^-1 and N^imid^-2, seen at approximately ∼34 MHz. Overall, the low field ENDOR spectrum of LPMO-12.5 indicates a significant change in the covalency of the three coordinating nitrogen nuclei, which is likely associated with the changes in the protonation state of the histidine brace. Likewise, at higher magnetic field positions, the ENDOR response spans a wider magnetic field range compared to the two samples prepared at lower pH (Fig. 5) with more defined features compared to LPMO-11.5. The full pattern can again be simulated under consideration of three strongly coupled nitrogen nuclei and using the molecular frame established for LPMO-6.5 and LPMO-11.5. The simulation of LPMO-12.5 has two ^14^N hyperfine tensors with much larger isotropic components compared to any nitrogen in the lower pH samples. These two approximately equivalent nitrogens with large isotropic couplings are assigned to N^imid^-1 and N^imid^-2 (Table 3). The consideration of a third strongly coupled nitrogen nucleus is required to complete the simulation. However, the nearly axial nature of the ***g***-tensor complicates the determination of the full hyperfine tensor. While the principal hyperfine values along *g*_1_ and *g*_3_ can be estimated from the pattern, the assignment of *A*_2_ is rather challenging and two distinct cases (case A and case B) yield similar and satisfactory ENDOR simulations (Fig. 5). Similarly, application of both parameter sets achieves almost identical nitrogen superhyperfine patterns in the X-band EPR spectra (Fig. S20†), precluding to favor one of the two cases from ENDOR and EPR.

The full parameters of case A and case B are reported in Table 3 and show a generally smaller *A*_2_ value of 37 MHz in case A, while case B employs a large value of 45 MHz, forming a more anisotropic tensor. In both cases the isotropic component of the hyperfine coupling *a*_iso_ decreases compared to the lower pH samples. The dipolar component, *t*, on the other hand, shows a slight decrease in case A and a large increase for case B relative to the lower pH samples. A further discussion of these two cases, including the impact of *t* on the nature of the Cu–N bond is provided in the ESI.† Notably, while the same nuclear quadrupole parameters were employed for LPMO-6.5 and LPMO-11.5, the nuclear quadrupole interactions in LPMO-12.5 (in both presented cases) are generally larger and more rhombic (Table 3). Together with the change in hyperfine interaction, this indicates significant electronic changes as the pH exceeds 11.5, associated with the threefold deprotonation of the histidine brace.

### A deprotonated copper amine ligand

UV-vis, EPR and the various hyperfine spectroscopies have revealed the deprotonation of the histidine brace at the two imidazole rings and the primary amine at pH 12.5. This deprotonation is accompanied by significant changes in the spectroscopic footprint of the tri-anionic histidine-brace, which can be correlated to electronic changes of the active site. As the amine's exact role in catalysis remains unclear, including the potential of this group to act as hydrogen atom donor for the protonation of copper–oxygen intermediates, the spectroscopic features of LPMO-12.5 offer some of the first insight into the electronic structure of such a deprotonated histidine brace.

Based on biomimetic studies,^20–23,25^ it was previously proposed that the anionic azanido ligand, Cu–(^−^NH-R), that is formed upon deprotonation of the amine in the histidine brace, would have increased donation strength compared to its protonated counterpart.^34^ This further led to the suggestion of a more covalent Cu–N–σ interaction, which should increase the nitrogen's isotropic hyperfine coupling. Contrary to this suggestion, for both presented cases for LPMO-12.5 the determined tensors exhibit smaller isotropic hyperfine interactions (*a*_iso_) compared to LPMO-6.5 and LPMO-11.5. The decreased Fermi contact term and, therefore, decreased s orbital spin population for the deprotonated N-terminal amine suggests a decreased Cu–N–σ-interaction and, potentially, a lengthening of the Cu–N^amine^ bond.

To the best of our knowledge, there are no reports on other copper(ii)-azanido complexes. However, Neubecker *et al.* previously reported on the deprotonation of an analogous Cu(iii) complex.^101^ They showed that deprotonation of the amine function in their Cu(iii)–(HNR_2_) complex leads to the formation of a strong Cu(iii)–azanido bond. Interestingly, they were not successful in deprotonating the corresponding Cu(ii) complexes, which was rationalized by the electronic structure differences in the Cu(iii) and potential Cu(ii) complexes. Generally, the deprotonation of the amine function would result in an additional lone pair on the formally sp^3^ amine, which is likely an unfavorable configuration, thus probably leading to rehybridization to a sp^2^ configuration and forming a lone pair in a N 2p_*z*_ orbital. Neubecker *et al.* proposed that the high oxidation state of the Cu(iii) center and the negative charge at the nitrogen would bring the energies of the N 2p_*z*_ and the Cu 4p_*z*_ orbitals closer, enabling π-bonding for the d^8^ Cu(iii) complex, which is a stabilizing interaction for Cu(iii). For the Cu(ii) complexes such a mechanism is unlikely, since π bonding to the higher energy Cu(ii) 4p_*z*_ is not favorable. Furthermore, we cannot experimentally confirm a sp^2^ configuration at the azanido as this should have an increased s orbital spin population relative to the p spin population (see Table S6†). This trend is contrary to the observed spin population estimates as the s orbital spin population (*a*_iso_) is decreased for both case A and case B. Therefore, the hyperfine analysis does not support the rehybridization of the nitrogen atom, but we cannot firmly rule out this possibility.

More globally, despite the inability to definitively assign the hyperfine tensor of LPMO-12.5, the hyperfine analysis allows us to form some general conclusions about the entire histidine brace and total covalency. For instance, the sum of the s and p spin populations of all nitrogen donors provides an estimate of the total histidine brace spin population. This approach yields a 3N spin population of 0.25 and 0.26 for LPMO-6.5 and LPMO-11.5, respectively, while for LPMO-12.5 spin populations of 0.22 (case A) and 0.25 (case B) are obtained, showing either a slight decrease or no change. The lack of a significant change in the spin population of the histidine brace as a whole indicates that the active-site works to maintain some sort-of charge neutrality, or better yet, constant spin delocalization. This may perhaps reflect the fundamental soft acid character of the metal and the hard base character of the ligands and their resistance to increase their covalent interaction. We also note here that cooperative effects regarding the σ-donating properties of the three coordinating nitrogen nuclei cannot be excluded. It is conceivable that the increased σ-donation of the two imidazolate ligands and the weakening of the copper-azanido bond are not independent phenomena but possibly opposing trends to retain a constant spin delocalization within the histidine brace.

Upon deprotonation of LPMO-11.5 to form LPMO-12.5, we also observe a decrease of the *g*_‖_ value, an increase in the *A*_‖_ and a blue shifted d–d band. The copper metal hyperfine has various contributions and is often used to interpret and/or estimate metal–ligand covalency. As noted above, there is no significant increase in the histidine brace's total spin population at pH 12.5 relative to the samples at lower pH. This suggests that there is not a significant change in the overall M–L covalency for the tri-anionic histidine brace. A large change in covalency would also influence the copper hyperfine due to spin delocalization, with a decreased *A*_‖_ value for increased M–L interaction. Rather, the observed increase of the copper's *A*_‖_ value is consistent with the ligand field influence on the metal hyperfine splitting, where the decreased *g*_‖_ value results in a smaller orbital dipolar contribution increasing the observed *A*_‖_.^102^ This draws a cohesive picture with the expected increased ligand charge upon deprotonation, that is well established to correlate with increased metal hyperfine.^83^

Altogether, the deprotonation of the amine function in LPMO-12.5 leads (in each analyzed case) to a less covalent interaction between the copper center and the primary amine, contrary to what has been proposed in the past. A potential reason for this contrast might be the concurrent deprotonation of the imidazole moieties.

### Protonation states of the histidine brace

Up to pH 11.5, the histidine brace itself stays remarkably unchanged, as revealed by the mostly unaltered hyperfine and nuclear quadrupole parameters for the three coordinated and the two weakly coupled nitrogen atoms. The major difference between LPMO-6.5 and LPMO-11.5 is attributed to the difference in coordinated H_2_O/OH^−^ ligands (and the concomitant geometry change), changing from two water molecules to one hydroxyl ion. These observations highlight the robustness and highly conserved nature of the histidine brace.

This rigidity in the histidine brace becomes even more apparent at pH > 11.5, where despite the strongly alkaline conditions, the histidine brace stays intact and no significant copper leakage is detected. As shown above, the histidine brace undergoes a threefold deprotonation, which includes the amine function and the remote nitrogen nuclei of the coordinating imidazole rings. While the order in which the deprotonation steps occur is not determined, the respective estimated p*K*_a_ values lie all clearly outside of what is typically considered the physiological range (p*K*_a2_ = 11.97, p*K*_a3_ = 12.02, p*K*_a4_ = 12.30). Of note, possible cooperative effects of the various functional groups are not considered here, since these cannot be determined with the experiments described above. Cooperativity has been shown previously in macrocyclic Cu(ii) complexes, as deprotonation of certain donors can increase the electron donation to the copper center, thereby making the copper more acidic, and decreasing the p*K*_a_ of other protonated donors.^103^

Deprotonation of the amine function has been proposed several times in various contexts,^2,5,21,34,37^ supported by the rather low p*K*_a_ values that were found for several synthetic Cu(ii) amides (p*K*_a_ ∼8–9)^103^ and Cu(iii) amines (p*K*_a_ ∼8–10).^35^ Our results indicate a higher p*K*_a_ value for *Sm*AA10A in its Cu(ii) state of approximately 12. Potential additional interactions caused by protein dynamics or protein–substrate interactions during turnover, may decrease the local, effective p*K*_a_ of the copper coordinated amine, so that the deprotonation of the amine function during catalysis cannot be excluded. Furthermore, while this p*K*_a_ appears high, the formation of a one-electron oxidized intermediate (*e.g.* [CuO]^+^) should further decrease the p*K*_a_, making the amine H-atom tautomerization feasible (where the H-atom from the amine transfers to the oxygen to form a [CuOH]^2+^ intermediate). Additionally, our results demonstrate that the determined deprotonation events emerge within a small pH window, necessitating the consideration of all three coordinating nitrogen ligands, when the protonation state of the histidine brace is discussed. In fact, imidazole coordination to Cu(iii) complexes compared to harder carboxylate ligands has been shown to decrease the p*K*_a_ of the amine proton of coordinated primary amines, directly demonstrating the influence of histidines to other groups in the histidine brace.^35^ Until now, the character and protonation states of the imidazole ring(s) has been widely neglected. Previous computational studies showed that the p*K*_a_ of free imidazole (∼14) can drop by 2–7 units when coordinated to copper^40^ but this has yet to be directly studied. Our experiments agree with a lowered p*K*_a_ of the imidazoles for *Sm*AA10A. Knowing second sphere residues play essential roles in tuning the reactivity of the active sites in LPMOs,^104–106^ potential H-bonding and the vicinity of charged amino acid side chains can further perturb the imidazole p*K*_a_ to additionally tune the reactivity of the active site.

### Stabilization of intermediates

When envisioning possible catalytic intermediates, we are often inspired by functional biomimetic chemistry. Numerous monocopper complexes are able to activate relatively strong C–H bonds, up to ∼80 kcal mol^−1^, *via* the formation of copper-superoxo intermediates.^107^ However, the majority of synthetic copper complexes do not have the oxidation strength to break stronger C–H bonds as found in, for example, crystalline cellulose. To overcome the high activation barrier necessary to break glycosidic bonds through C–H bond activation, highly reactive copper oxygen species have been invoked, such as a copper(ii)-oxyl, [CuO]^+^, or its conjugate acid, a copper(iii)-hydroxo, [CuOH]^2+^, mostly based on computational studies.^12^ However, such intermediates have only been identified in gas phase experiments for small molecule systems and their observation remains entirely elusive in biological samples.^27,28^ Despite the lack of direct observation of such reactive intermediates in biological catalysis so far, they continue to inspire further investigations and synthetic modeling.

**Scheme 3:**
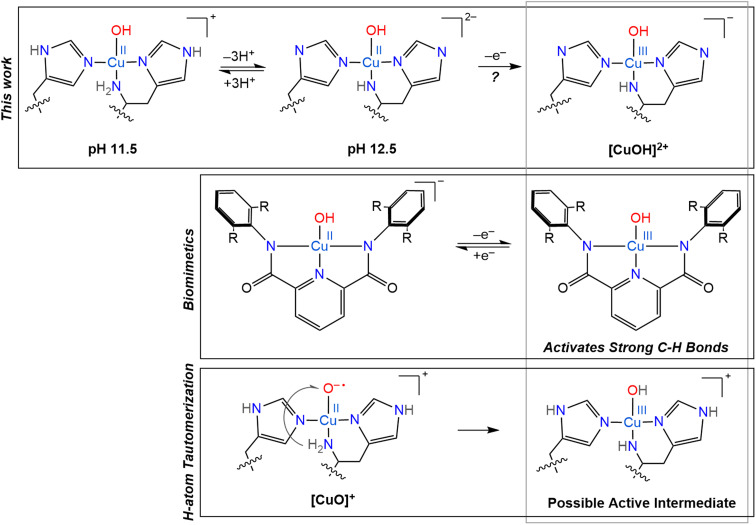
Overview of several copper oxygen complexes, including the LPMO active sites generated in this work and its potential Cu(iii) counterpart (top), proposed H-atom tautomerization at the LPMO active site (bottom) and the biomimetic copper complexes synthesized by Tolman and coworkers (middle).^20–23^

One standout series of biomimetic copper complexes is the above-mentioned set of Cu(iii)–OH complexes from Tolman and coworkers with di-anionic carboxamide ligands (Scheme 3). These have demonstrated fast hydrogen atom abstraction (HAA) and strong C–H bond activation (up to 99 kcal mol^−1^), similar to the chemistry of LPMOs and pMMOs. It is the electron-rich, di-anionic ligand employed by Tolman and co-workers that is able to effectively stabilize the [CuOH]^2+^ core.^21–23^ The high basicity and excellent σ-donor properties of these ligands reduce the Cu(ii)/Cu(iii) redox potential (which stabilizes the Cu(iii) state), but also increase the basicity of the hydroxide unit and therefore drive the rapid HAA, as shown by ligand variation studies.^22^

Based on their small molecule chemistry, Tolman and coworkers proposed that in LPMOs intermediates with a [CuOH]^2+^ core would be viable intermediates, potentially forming *via* protonation of a copper(ii)-oxyl [CuO]^+^ species. Such a proton could either originate from an external source or from tautomerism with the amine function to generate a copper-azanido species (Scheme 3).^21^ Ryde and coworkers showed that the deprotonation of the N-terminal amine function can significantly reduce the reduction potential of reaction intermediates, making such an intermediate more likely.^12^ However, these previous calculations did not consider a possible deprotonation of the remote nitrogen nuclei of the coordinating imidazole groups, which, as we show here, have similar p*K*_a_ values as the amine.

Another reason to consider imidazole deprotonations is that, previously, a histidine residue nearby the active site of *Nc*AA9D was observed to undergo deprotonation upon reaction of the copper with oxygen, and therefore proposed to act as proton donor to help convert the superoxo to a peroxo state.^39^ Although this additional nearby histidine residue is lacking in *Sm*AA10A, similar proton transfer around the active site in *Sm*AA10A is imaginable, even *via* the coordinating histidine residues, potentially supported by H-bonding of a nearby glutamate residue, to further stabilize the copper core through increased σ-donation, as shown by our nitrogen ENDOR experiments. The amine function, on the other hand, seemingly becomes a worse σ-donor upon deprotonation, at least in the triply deprotonated histidine brace, in contrast to previous suggestions.^34^

The increased donor strength of the imidazolates may in fact be the key to stabilization of a potent [CuOH]^2+^ oxidant, rather than only the amine group. In some LPMOs, the N-terminal histidine may be methylated in a post translational modification. This methylation does not knowingly affect the catalytic function of the protein and has a minimal effect on the redox potential.^108^ However, it was observed that the methylated active-site withstood excess H_2_O_2_ treatment better than its non-methylated counterpart, showing that methylation plays a role in protection of the protein from oxidative damage. Our findings raise an interesting question if the methylation of the N-terminal histidine can have a catalytic effect, either on- or off-pathway, that so far has remained undiscussed.

The similar characteristics observed between the LPMO-12.5 copper site and that of the anionic ligand stabilizing the [CuOH]^+^ and [CuOH]^2+^ cores of biomimetic complexes are curious and inspire the further characterization of the LPMO resting state at high pH. The high pH tolerance of *Sm*AA10A is particularly noteworthy. Previous research^45^ has demonstrated that this LPMO exhibits a compact and rigid structure, which may account for its ability to fully regain activity even after 16 hours of incubation at pH 12.5. The previous spectroscopic analysis indicates that proton transfer occurs as the pH increases, without causing significant alterations in the histidine coordination to the copper center. These observations, taken together with these new findings, suggest that LPMOs possess an inherent robustness to elevated pH conditions, which is a beneficial trait for a bacterial enzyme, potentially due to their structural stability and metal coordination environment.

## Concluding remarks

In conclusion, *Sm*AA10A was characterized over a wide pH range (pH 4.0 to 12.5), in which the protein underwent several reversible chemical transitions.

The first one (p*K*_a1_ = 9.65) was found to be associated with a change of coordinated solvent from two waters to one hydroxo ligand. This is accompanied by a change of the overall geometry of the active site from a trigonal bipyramidal to square planar coordination environment leading to an axial instead of the originally observed rhombic ***g***-tensor. The histidine brace itself stays fairly untouched upon this transition, as shown by the unmodified hyperfine and quadrupole parameters of the strongly and weakly coupled nitrogen nuclei.

Above pH 11.5 three additional transitions were observed within a small pH range (p*K*_a2_ = 11.97, p*K*_a3_ = 12.02, p*K*_a4_ = 12.30) that were, based on ^1,2^H and ^14,15^N ENDOR, ^14^N ESEEM and ^15^N HYSCORE experiments, assigned to a threefold deprotonation of the histidine brace at the amine function and the two remote nitrogen nuclei of the imidazole moieties. Such a rather high p*K*_a_ value for the amine deprotonation disagrees with previous proposals suggesting deprotonation under physiological conditions to potentially stabilize reaction intermediates. However, in our tri-anionic histidine brace the amine function was found to exhibit significantly reduced σ-donating properties compared to its protonated form, rather destabilizing the copper core. Instead, the coordinated nitrogen nuclei of the imidazole moieties display significantly stronger σ-donating properties, adopting such a stabilizing function. The determined close proximity of the three deprotonation events accentuates the necessity to consider not just the protonation state of the amine function, but of the imidazole moieties as well when mechanistic evaluations are made, something that has been widely neglected in the past.

In that sense, we hope that our findings inspire future mechanistic considerations. Additionally, the full determination of the spin Hamiltonian Parameters for the nitrogen nuclei of the histidine brace is the to date most detailed description of the bonding situation at the LPMO active site and sets standards for future projects involving LPMOs under turnover conditions.

## Data availability

The data supporting this article have been included as part of the ESI† and all data presented in the article are available at Edmond (the Open Data Repository of the Max Planck Society) at https://doi.org/10.17617/3.boletg. Additional citations are made within the ESI†.^109–127^

## Author contributions

JH performed all EPR, ENDOR, ESEEM, HYSCORE and UV-vis experiments and analysis under the supervision of GEC. OG expressed and purified the protein and performed the activity assays and the thermal shift analysis under the supervision of VGHE. JH wrote the original draft with contributions of OG (biochemical characterization) and GEC. All authors contributed to the final manuscript. GEC conceptualized the experiments. GEC and VGHE acquired funding.

## Conflicts of interest

There are no conflicts to declare.

## Supplementary Material

SC-016-D4SC04794J-s001
