# Supplementary material for: pH-mediated manipulation of the histidine brace in LPMOs and generation of a tri-anionic variant, investigated by EPR, ENDOR, ESEEM and HYSCORE spectroscopy
Source: Chem Sci. 2024 Nov 26;16(1):233–54. doi: 10.1039/d4sc04794j (PMC11590009; doi:10.1039/d4sc04794j)
Supplement: SC-016-D4SC04794J-s001 [file SC-016-D4SC04794J-s001.pdf]

Supplementary Information (SI) for Chemical Science.
This journal is © The Royal Society of Chemistry 2024

Supporting Information

pH-mediated Manipulation of the Histidine Brace in LPMOs and Generation of a Tri-anionic Variant, Investigated by EPR, ENDOR, ESEEM and HYSCORE Spectroscopy

Julia Haak,[a,b] Ole Golten,[c] Morten Sørlie,[c] Vincent G.H. Eijsink,[c] George E. Cutsail III[a,b,*]

[a] Max Planck Institute for Chemical Energy Conversion, Stiftstrasse 34-36, D-45470 Mülheim an der Ruhr, Germany

[b] Institute of Inorganic Chemistry, University of Duisburg-Essen, Universitätsstrasse 5-7, D-45141, Essen, Germany

[c] Faculty of Chemistry, Biotechnology and Food Science, NMBU - Norwegian University of Life Sciences, N-1432 Ås, Norway

email: george.cutsail@cec.mpg.de

Table of Contents

Figure S1. EPR of Deuterated Samples. 3
Singular Value Decomposition of the UV-Vis titration of *Sm*AA10A....4
Figure S2. SVD analysis of UV-Vis pH titration spectra for *Sm*AA10A. 4
Scheme S1. Scheme of the proposed stepwise deprotonation of the histidine brace. 5
Figure S3. Fitting of the SVD analysis. 7
Stability and Activity Studies....8
Figure S4. Chitin degradation by *Sm*AA10A after preincubation at various pH....8
Figure S5. Thermal shift analysis of *Sm*AA10A....9
EPR Spectroscopy of *Sm*AA10A....10
Figure S6. CW X-band EPR spectra of *Sm*AA10A. 11
Table S1. Summary of the EPR parameters for the four species detected by X-band EPR. 12
Table S2. Relative amounts of the individual species, estimated by X-band EPR simulations....12
Figure S7. Q-band pulsed EPR spectra of *Sm*AA10A. 14
Table S3. Spin Hamiltonian parameters for the species observed with Q-band EPR....14
^{1}H Davies and ^{2}H Mims ENDOR Spectroscopy 15
Figure S8. ^{1}H Davies ENDOR spectra....15
Figure S9. Q-band ^{1}H Davies ENDOR spectra of *Sm*AA10A at pH 12.5. 15
Figure S10. Comparison of Q-band ^{1}H Davies difference and ^{2}H Mims ENDOR spectra. 16
Three-Pulse ESEEM Spectroscopy 17
Figure S11. Direct comparison of time-domain and FT ESEEM of **LPMO-6.5**, **-11.5** and **-12.5**. 17
Figure S12. Three-pulse ESEEM spectra of **LPMO-6.5** and **LPMO-11.5**. 18
Figure S13. Three-pulse ESEEM fittings for **LPMO-6.5**. 21
Table S4. Nitrogen spin Hamiltonian parameters from fitted three-pulse ESEEM data of **LPMO-6.5**....22
Figure S14. RMSE surface plot for **LPMO-6.5** (e^2qQ/h (N1) vs e^2qQ/h(N2))**.** 24
Figure S15. RMSE surface plot for **LPMO-6.5** (e^2qQ/h vs. η). 25
Figure S16. Three-pulse ESEEM fittings for **LPMO-11.5**. .... **Error! Bookmark not defined.**
Table S5. Nitrogen spin Hamiltonian parameters from fitted three-pulse ESEEM data of **LPMO-11.5**....28
Figure S17. RMSE surface plot for **LPMO-11.5** (e^2qQ/h(N1) vs e^2qQ/h(N2)). 29
^{15}N HYSCORE Spectroscopy....30
Coordinates of Optimized Geometries (DFT calculated)....33
Coordinates of $[Cu(imidH)_4]^{2+}$....33
Coordinates of $[Cu(imid)_4]^{2-}$ 34
^{14,15}N ENDOR Spectroscopy and Differentiation between case A and case B 35
Figure S19. ^{14}N and ^{15}N Davies ENDOR spectra. 39
Table S6. Hyperfine and nuclear quadrupole parameters of the coordinated nitrogen nuclei**.** 40
Figure S20. X-band EPR simulations of **LPMO-12.5 case A** and **case B**. 41
References....42

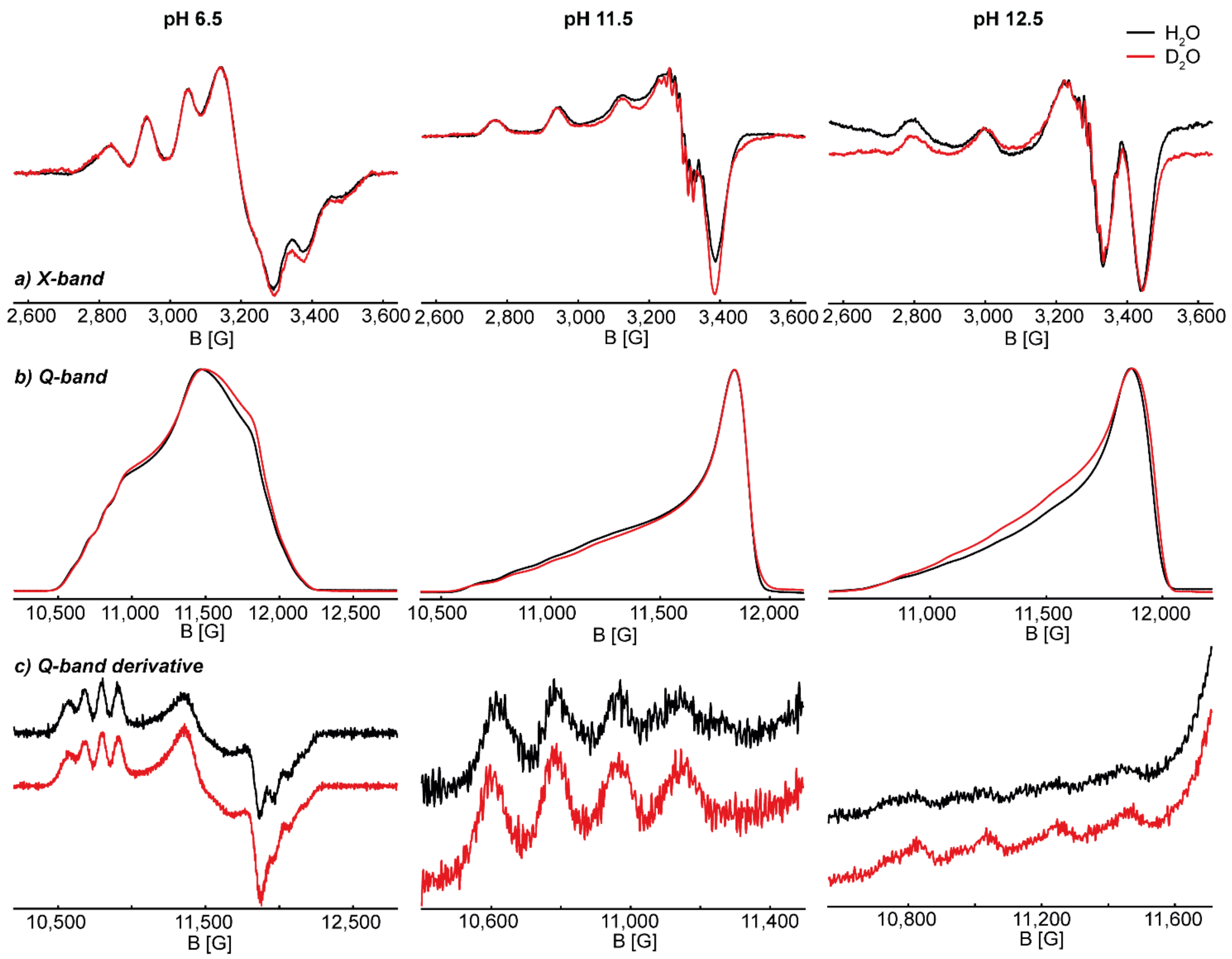

Figure S1. EPR of Deuterated Samples.
Comparison of **a)** the CW X-band (~9.46 GHz) EPR spectra, **b)** the as-collected pulsed Q-band (33.97 – 34.01 GHz) EPR spectra and **c)** (excerpts of) their numerical derivative (smoothed over one to five points depending on the sample) for *Sm*AA10A at pH 6.5 (left), pH 11.5 (middle) and pH 12.5 (right) in H_2O (black) and D_2O (red). The spectra of the two samples are in good agreement confirming the validity of the chosen cancel-out approach (see Experimental Section). Experimental conditions are reported in the Experimental Section.

Singular Value Decomposition of the UV-Vis titration of *Sm*AA10A

To analyze the UV-Vis pH titration spectra, a singular value decomposition (SVD) was performed. SVD is a powerful tool that can help to extract information on the number of species that emerge and disappear in the course of the titration, their ratios at the respective pH values and, consequently, their pK_a values.[1,2] Generally, an SVD describes the mathematical process of finding the so-called basis vectors of a set of initial vectors. Any of these can then be reconstructed through a linear combination of the basis vectors.

To apply this to spectroscopic titrations, the experiments can be summarized in a matrix **A**, where every column represents a spectrum at a certain control variable (e.g. pH). By employing the SVD to the data matrix **A**, it is divided into **U**, **S** and **V**: $\mathbf{A} = \mathbf{USV}^{\mathrm{T}}$. **U** has $m \times n$ elements and consists of the basis vectors, **S** is a $n \times n$, diagonal matrix and comprises the Singular Values in decreasing fashion and **V** is composed of $n \times n$ elements that hold information about how much of the individual basis spectra are required to reconstruct **A**.

For our UV-Vis titration of *Sm*AA10A, (**Figure 1** in the main text) the data matrix **A** has 500 x 20 elements, where a_{ik} is the absorbance of the sample at pH$_k$ measured at λ_i. The SVD of our UV-Vis titration yields five singular values of reasonable size ($s_{5,5} \approx 1$ % of $s_{1,1}$; **Figure S2a**), indicating the presence of five spectroscopically distinguishable species. Accordingly, the basis spectra show a significant decrease in S/N ratio for species six (**Figure S2b**).

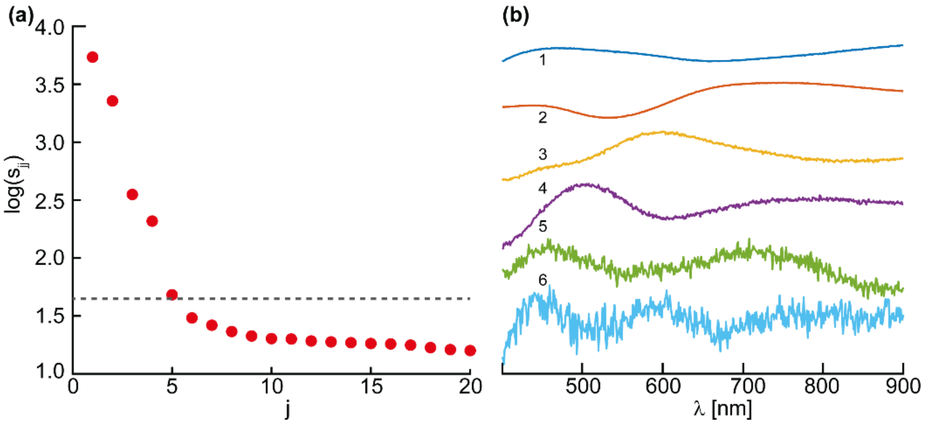

Figure S2. SVD analysis of UV-Vis pH titration spectra for *Sm*AA10A.
a) s_{jj} of the individual species in **A** on a logarithmic scale, showing five major contributors to the SVD and **b**) basis vectors of species j = 1 to 6.

By the means of EPR and Hyperfine spectroscopy a total of five species were established, reinforcing the findings of the UV-Vis SVD analysis (see next section). The number of evolving species can be rationalized by the stepwise deprotonation of the histidine brace, **Scheme S1**. Depending on the order of the deprotonation steps, either a species with singly and/or doubly deprotonated imidazoles, but protonated amine function, or a

deprotonated amine function and partly deprotonated imidazole rings is plausible in the intermediate pH range between 11.5 and 12.5.

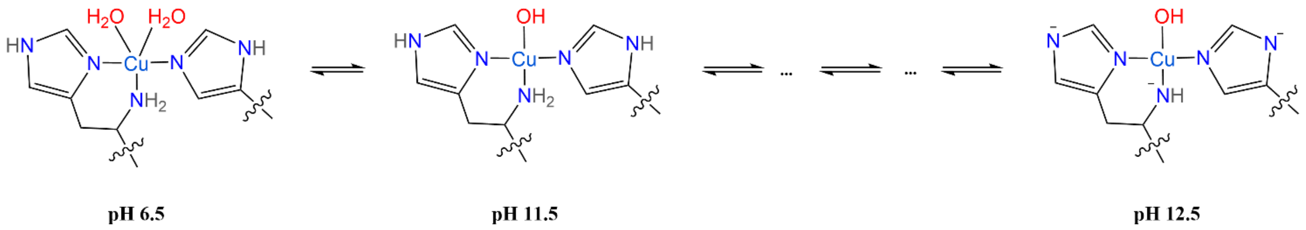

Scheme S1. Scheme of the proposed stepwise deprotonation of the histidine brace.

To further analyze the matrices obtained through SVD, only the first t columns in **U**, **S** and **V** are retained, forming the three new matrices $\bar{\boldsymbol{U}}(\boldsymbol{m}\times\boldsymbol{t})$, $\bar{\boldsymbol{S}}(\boldsymbol{t}\times\boldsymbol{t})$ and $\bar{\boldsymbol{V}}(\boldsymbol{n}\times\boldsymbol{t})$. t is defined as *number of species* +1. In our case, we set $t = 6$, to account for the five possible species discussed above.

The information obtained from the SVD can then be interpreted with an appropriate chemical model. Generally, our data matrix **A**, can be described as $\mathbf{A} = \mathbf{DF}^{T}$, where **D** describes a matrix of difference spectra and **F** comprises transition functions. The transitions functions $\mathbf{F}_{kj} = \alpha_j(\mathrm{pH}_k)$ describe the amount of species j at pH_k and hold therefor information on the $\mathrm{p}K_a$ values of the individual species. A general model for a five-component system is derived as follows:

Based on the here reported data, the active site of *Sm*AA10A is likely to be deprotonated in four positions: As seen by EPR spectroscopy, the first formal deprotonation event involves the change of the coordinated solvent (H_2O to OH^-). The deprotonation of the remote nitrogen atoms on the two coordinated imidazole rings and the amine function follow. The concentration of these five species and their equilibrium constants can be described as follows:

$$AH_4 \rightleftharpoons AH_3^- + H^+ \qquad K_{a1} = \frac{[AH_3^-][H^+]}{[AH_4]}$$

$$AH_3^- \rightleftharpoons AH_2^{2-} + H^+ \qquad K_{a2} = \frac{[AH_2^{2-}][H^+]}{[AH_3^-]}$$

$$AH_2^{2-} \rightleftharpoons AH^{3-} + H^+ \qquad K_{a3} = \frac{[AH^{3-}][H^+]}{[AH_2^{2-}]}$$

$$AH^{3-} \rightleftharpoons A^{4-} + H^+ \qquad K_{a4} = \frac{[A^{4-}][H^+]}{[AH^{3-}]}$$

The relative amount of species j is described by α_j with:

$$\alpha_0 = \frac{[AH_4]}{[A_{tot}]} \qquad \alpha_1 = \frac{[AH_3^-]}{[A_{tot}]} \qquad \alpha_2 = \frac{[AH_2^{2-}]}{[A_{tot}]} \qquad \alpha_3 = \frac{[AH^{3-}]}{[A_{tot}]} \qquad \alpha_4 = \frac{[A^{4-}]}{[A_{tot}]}$$

and

$$[A_{tot}] = [AH_4] + [AH_3^-] + [AH_2^{2-}] + [AH^{3-}] + [A^{4-}]$$

substitution of α_j into the equilibrium constants yields

$$K_{a1} = \frac{[A_{tot}]\alpha_1[H^+]}{[A_{tot}]\alpha_0} = \frac{\alpha_1[H^+]}{\alpha_0}$$

$$K_{a2} = \frac{[A_{tot}]\alpha_2[H^+]}{[A_{tot}]\alpha_1} = \frac{\alpha_2[H^+]}{\alpha_1}$$

$$K_{a3} = \frac{[A_{tot}]\alpha_3[H^+]}{[A_{tot}]\alpha_2} = \frac{\alpha_3[H^+]}{\alpha_2}$$

$$K_{a4} = \frac{[A_{tot}]\alpha_4[H^+]}{[A_{tot}]\alpha_3} = \frac{\alpha_4[H^+]}{\alpha_3}$$

Expressing all species in relation to α_0 leads to:

$$\alpha_1 = \frac{K_{a1}\alpha_0}{[H^+]}$$

$$\alpha_2 = \frac{K_{a2}\alpha_1}{[H^+]} = \frac{K_{a1}K_{a2}\alpha_0}{[H^+]^2}$$

$$\alpha_3 = \frac{K_{a3}\alpha_2}{[H^+]} = \frac{K_{a1}K_{a2}K_{a3}\alpha_0}{[H^+]^3}$$

$$\alpha_4 = \frac{K_{a4}\alpha_3}{[H^+]} = \frac{K_{a1}K_{a2}K_{a3}K_{a4}\alpha_0}{[H^+]^4}$$

The sum of all fractions has to equal 1:

$$\alpha_0 + \alpha_1 + \alpha_2 + \alpha_3 + \alpha_4 = \alpha_0 + \frac{K_{a1}\alpha_0}{[H^+]} + \frac{K_{a1}K_{a2}\alpha_0}{[H^+]^2} + \frac{K_{a1}K_{a2}K_{a3}\alpha_0}{[H^+]^3} + \frac{K_{a1}K_{a2}K_{a3}K_{a4}\alpha_0}{[H^+]^4} = 1$$

This allows to determine the relative amounts of all species:

$$\alpha_0 = \frac{1}{1 + \frac{K_{a1}}{[H^+]} + \frac{K_{a1}K_{a2}}{[H^+]^2} + \frac{K_{a1}K_{a2}K_{a3}}{[H^+]^3} + \frac{K_{a1}K_{a2}K_{a3}K_{a4}}{[H^+]^4}}$$

$$\alpha_1 = \frac{K_{a1}}{[H^+]}\frac{1}{1 + \frac{K_{a1}}{[H^+]} + \frac{K_{a1}K_{a2}}{[H^+]^2} + \frac{K_{a1}K_{a2}K_{a3}}{[H^+]^3} + \frac{K_{a1}K_{a2}K_{a3}K_{a4}}{[H^+]^4}}$$

$$\alpha_2 = \frac{K_{a1}K_{a2}}{[H^+]^2}\frac{1}{1 + \frac{K_{a1}}{[H^+]} + \frac{K_{a1}K_{a2}}{[H^+]^2} + \frac{K_{a1}K_{a2}K_{a3}}{[H^+]^3} + \frac{K_{a1}K_{a2}K_{a3}K_{a4}}{[H^+]^4}}$$

$$\alpha_3 = \frac{K_{a1}K_{a2}K_{a3}}{[H^+]^3} \frac{1}{1 + \frac{K_{a1}}{[H^+]} + \frac{K_{a1}K_{a2}}{[H^+]^2} + \frac{K_{a1}K_{a2}K_{a3}}{[H^+]^3} + \frac{K_{a1}K_{a2}K_{a3}K_{a4}}{[H^+]^4}}$$

$$\alpha_4 = \frac{K_{a1}K_{a2}K_{a3}K_{a4}}{[H^+]^4} \frac{1}{1 + \frac{K_{a1}}{[H^+]} + \frac{K_{a1}K_{a2}}{[H^+]^2} + \frac{K_{a1}K_{a2}K_{a3}}{[H^+]^3} + \frac{K_{a1}K_{a2}K_{a3}K_{a4}}{[H^+]^4}}$$

A linear combination of the so derived transition function can then be used to model matrix $\overline{\boldsymbol{V}}$ for every species *j* at pH$_k$. The elements of the $t \times t$ matrix **H**=h_{kj} are used as coefficients to form the following matrix for our UV-Vis titration:

$$\overline{\boldsymbol{V}} \mathrel{\widehat{=}} \widehat{\boldsymbol{F}} = \begin{bmatrix} h_{6,1} + h_{1,1}\alpha_0(pH_{k=1}) + h_{2,1}\alpha_1(pH_{k=1}) + \cdots + h_{5,1}\alpha_4(pH_{k=1}) & \cdots & h_{6,6} + h_{1,6}\alpha_0(pH_{k=1}) + h_{2,6}\alpha_1(pH_{k=1}) + \cdots + h_{5,6}\alpha_4(pH_{k=1}) \\ \vdots & \ddots & \vdots \\ h_{6,1} + h_{1,1}\alpha_0(pH_{k=20}) + h_{2,1}\alpha_1(pH_{k=20}) + \cdots + h_{5,1}\alpha_4(pH_{k=20}) & \cdots & h_{6,6} + h_{1,6}\alpha_0(pH_{k=20}) + h_{2,6}\alpha_1(pH_{k=20}) + \cdots + h_{5,6}\alpha_4(pH_{k=20}) \end{bmatrix}$$

To determine the pK_a values of *Sm*AA10A, matrix $\widehat{\boldsymbol{F}}$ is fitted to $\overline{\boldsymbol{V}}$, weighted by $\overline{\boldsymbol{S}}^2$, via a least-squares fitting with a generic algorithm and random starting points for the elements of **H**:

$$\phi = |\overline{\boldsymbol{S}}^2\overline{\boldsymbol{V}}^{\boldsymbol{T}} - \overline{\boldsymbol{S}}^2\overline{\boldsymbol{V}}^{\boldsymbol{T}}(\widehat{\boldsymbol{F}}^{\boldsymbol{T}})^+\widehat{\boldsymbol{F}}^{\boldsymbol{T}}|^2 \rightarrow 0$$

The superscript "+" denotes the Moore-Penrose-Pseudoinverse. By setting a constraint of p$K_{a1} \leq$ p$K_{a2} \leq$ p$K_{a3} \leq$ pK_{a4} and appropriate bounds and starting points for the pK_a values, a least-squares minimum with the following parameters is found, **Figure S3a**:

pK_{a1} = 9.65 pK_{a2} = 11.97 pK_{a3} = 12.02 pK_{a4} = 12.30

Next, the difference spectra $\widehat{\boldsymbol{D}} = \mathbf{A}(\widehat{\boldsymbol{F}}^{\boldsymbol{T}})^+$ and thereby $\widehat{\boldsymbol{A}} = \widehat{\boldsymbol{D}}\widehat{\boldsymbol{F}}^{\boldsymbol{T}}$ can be calculated. Comparison of the experimental data **A** and the reconstructed data $\widehat{\boldsymbol{A}}$ shows excellent agreement, **Figure S3b**.

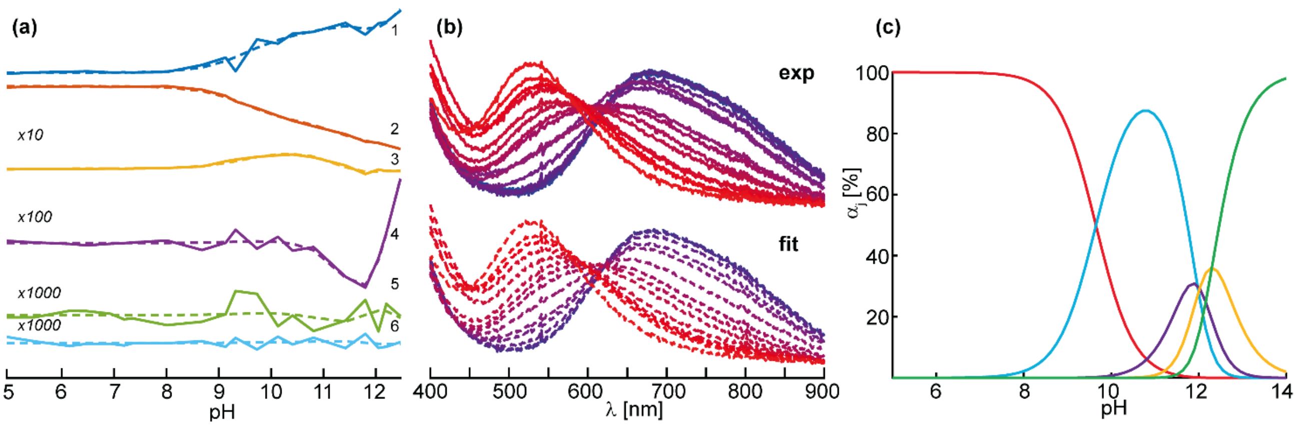

Figure S3. Fitting of the SVD analysis.
(a) Results of the least-squares fitting of the UV-Vis SVD: Experimental values ($\overline{\boldsymbol{S}}^2\overline{\boldsymbol{V}}^{\boldsymbol{T}}$) are depicted in solid, the final fits ($\overline{\boldsymbol{S}}^2\overline{\boldsymbol{V}}^{\boldsymbol{T}}(\widehat{\boldsymbol{F}}^{\boldsymbol{T}})^+\widehat{\boldsymbol{F}}^{\boldsymbol{T}}$) are plotted in dashed lines. **(b)** UV-Vis spectra of *Sm*AA10A at 20 pH values in solid lines compared to the reconstructed data from the SVD analysis in dashed lines. **(c)** Contributions of the individual species over the modelled pH range.

Stability and Activity Studies

Due to a lack of sufficiently strong buffers, assessment of enzymatic activity at pH 11.5 and 12.5 was not possible. The stability of *Sm*AA10A at pH 6.5, 11.5 and 12.5 was investigated by storing the enzyme in 50 mM of the mixed buffer system for either 2 minutes or 16 hours. Following the storage, chitin degradation reactions were performed in 50 mM Tris-HCl, pH 7.0, by diluting the stored enzyme solutions into an appropriate reaction mixture. The resulting product formation curves are presented in **Figure S4**.

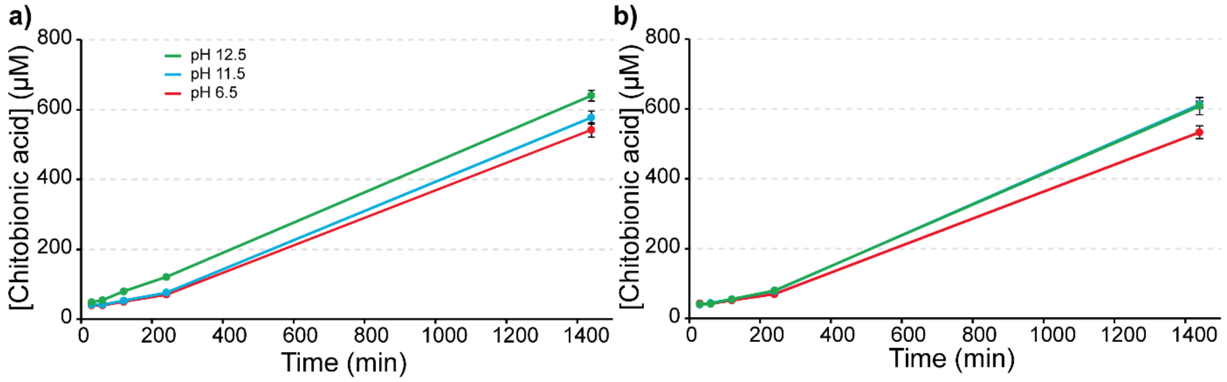

Figure S4. Chitin degradation by *Sm*AA10A after preincubation at various pH.
Enzyme solutions at a concentration of 100 µM in 50 mM MES/HEPES/CHES/CAPS buffer, pH 6.5, 11.5 or 12.5, were incubated for 16 hours at 4 °C (a) or 2 minutes stored on ice (b). Subsequently, these pre-incubated enzyme solutions were diluted 100 times in a reaction solution with final concentrations of 1 µM *Sm*AA10A, 10 $g \cdot L^{-1}$ squid pen β-chitin and 1 mM ascorbate in 50 mM Tris-HCl, pH 7.0, followed by incubation at 40 °C with 850 rpm agitation. For both reaction setups, samples, taken at regular time intervals were filtered, after which soluble oxidized products were enzymatically converted to the oxidized dimer for easy quantification. Data points in both A and B represent triplicate reactions and standard deviations are shown as error bars (n=3).

The activity data shows that storing *Sm*AA10A at pH 6.5, 11.5 or 12.5 does not lead to irreversible inactivation of the enzyme. Minor increases in activity upon storage at elevated pH (best visible for the green curve in panel A) could be due to some degree of inactivation and subsequent copper release. This paradoxal effect is due to the fact that reductant-driven LPMO reactions are limited by the *in situ* generation of H_2O_2. Free-copper promotes oxidation of ascorbate to generate H_2O_2, which leads to increased LPMO catalysis.[3] Importantly, the increase in reaction rate is very modest, which shows that the large majority of LPMOs still has an intact copper-binding site and binds copper. High concentrations of free copper that approach the concentration of enzyme in the reaction would lead to rapid formation of H_2O_2, resulting in enzyme inactivation and ascorbate depletion[3–5], and linear formation of product over 24 hours would not be observed under such conditions.[5,6] Of note, the slow release of oxidized product over 24 hours by *Sm*AA10A under these reaction conditions (ascorbate-driven, neutral pH) concurs with previous observations.[7,8]

Enzyme stability at pH 6.5, 11.5 and 12.5 was further investigated by performing an analysis of temperature dependent unfolding using the SYPRO® orange thermal shift method (**Figure S5**).

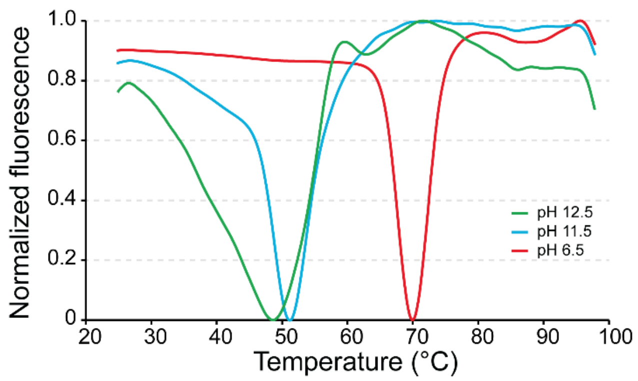

Figure S5. Thermal shift analysis of *Sm*AA10A.
The analysis was performed with a temperature ramp of 1.5 °C min^{-1} from 25-98°C with 50 µM SmAA10A, 1x SYPRO® orange in 50 mM MES/HEPES/CHES/CAPS buffer at pH 6.5 (red), 11.5 (blue) or 12.5 (green). The results are presented as the first derivative of fluorescence intensity normalized using min-max normalization ($f_{norm.} = \frac{f - f_{min}}{f_{max} - f_{min}}$). The shown curves represent one experiment; standard deviations in apparent melting temperatures were measured for n=4.

The apparent melting temperature of *Sm*AA10A at pH 6.5 is 69.9±0.1 °C , which is similar to previously published values[9]. Increasing the pH decreased the apparent melting temperature to 51.1±0.1 and 48.6±0.3 °C, for pH 11.5 and 12.5, respectively. At pH 12.5 the unfolding peak broadens, indicative of changes in the unfolding process that may indicate a larger degree of instability compared to pH 11.5 (**Figure S5**). Of note, at all three pH values, the apparent melting temperature is well above the reaction temperatures used in this study, which were maximally 40 °C, in the activity assay shown in **Figure S4**.

EPR Spectroscopy of *Sm*AA10A

To further probe the property changes of the Cu(II) center as the pH is increased, continuous-wave (CW) X-band (9.46 GHz) EPR spectra of samples between pH 4.0 and pH 12.5 were obtained (**Figure S6**).

At the lowest pH, a rhombic EPR spectrum ($g_1 > g_2 > g_3$) is observed with a broad EPR linewidth and no resolved nitrogen hyperfine splitting. The EPR spectrum is well simulated with a rhombic **g-** and **A**($^{63/65}$Cu)-tensor (**g** = [2.258 2.096 2.023], **A** = [350 100 240] MHz), in excellent agreement to the parameters recently employed by Munzone et al. (**g** = [2.258 2.114 2.024], **A** = [375 57 268] MHz).[10] The signal is assumed to be associated with a single chemical species, **LPMO-A**, and remains unchanged for the sample at neutral pH (pH 6.5, '**LPMO-6.5**'). At slightly alkaline conditions (pH 9), a new, additional feature at lower field is detected and sharp resolved ^{14}N superhyperfine begins to appear around g~2 (3,200 – 3,400 G), signifying the formation of a second species with a more axial EPR **g**-tensor ($g_{\parallel} > g_{\perp}$ or $g_1 > g_2 = g_3$). At pH 11.5 ('**LPMO-11.5**'), the EPR response (**Figure S6**) is fully converted to an axial EPR spectrum that is well simulated with a single set of spin Hamiltonian parameters and is associated with the second species **LPMO-B** (**Table S1**). When the pH is further increased, the spectra can be simulated as a mixture of two further new, axial species with resolved ^{14}N superhyperfine splitting, denoted as **LPMO-C** and **LPMO-D**: At pH 12.0 ('**LPMO-12.0**') the spectrum is dominated by species **LPMO-C** (~90 %), which is characterized by a decreased $g_{||}$ of 2.215 (compared to **LPMO-B**: 2.230) and a larger $A_{||}$ of 550 MHz (**LPMO-B**: 538 MHz). The formation of **LPMO-D** at pH 12.5 ('**LPMO-12.5**') is best marked by the growth of the high field feature ('overshoot feature') at 3,430 G belonging to the m_I(Cu) = +3/2 transition of $g_{\parallel}$ (**Figure S6**), a result of the even further increased $A_{\parallel}$(Cu) value of 605 MHz and a further reduction of $g_{\parallel}$ to 2.179. At pH 12.5, the protein has not fully converted to **LPMO-D**, but can be simulated as a mixture of 60 % **LPMO-D** and 40 % **LPMO-C**. The proportions of the individual components **LPMO-A**, **LPMO-B, LPMO-C** and **LPMO-D** in each EPR sample were estimated by multi-component simulation of each spectrum (**Tables S1 & S2**). The CW X-band EPR spectra of all samples are displayed in **Figure S6**.

Despite the strictly conserved histidine brace in all LPMOs, the EPR signatures observed vary in their spectral character: Besides classical axial EPR spectra with $g_{\perp}$ ~ 2.25 and $g_{\parallel}$ ~2.05 for systems with a dx^2-y^2 singly occupied molecular orbital (SOMO) (generally associated with square planar, square pyramidal or octahedral (elongated) coordination geometry), a range of rhombic spectra for LPMOs has been reported.[11–15] The latter can be attributed to systems with significant dz^2 admixing into the ground state, often observed for systems of lower symmetry.[16] In the case of LPMOs the leading analysis has focused on the water coordination as the driving force for symmetry modulation. For the Cu(II) resting state, the number of waters and/or hydroxyl ions coordinated to the Cu(II) site of the histidine brace modulates the local environment of the copper center and therefore significantly influences the EPR spectrum. This effect is illustrated by previous studies of substrate-binding to *Sm*AA10A.[17] To enable the enzyme-substrate interaction, rearrangement of one of the two coordinating water molecules is necessary, leading to a more axial EPR response. Similarly, it was shown for

*Bl*AA10 and *Pl*AA10 that changing the pH has a significant effect on the EPR spectrum: For *Bl*AA10 increasing the pH from 6.5 to 10.5 converted the EPR spectrum from largely rhombic ($\Delta R = 0.70$) to mostly axial ($\Delta R = 0.15$), where $\Delta R = (g_3\text{-}g_2)/(g_2\text{-}g_1)$ yields the degree of rhombic splitting with 1.0 being fully rhombic and 0 being axial.[14] *Pl*AA10 showed an analogous effect going from pH 6.5 ($\Delta R \sim 0.5$) to 8.5 ($\Delta R \sim 0.1$).[15] Lindley et al. hypothesized this behavior could be explained by a coordination change from 3N2O, originating from the histidine brace and two waters, to 2N2O species, with two hydroxide ligands and a decoordinated amine function.[14] However, the EPR simulations of the ^{14}N superhyperfine splitting pattern and computational studies of *Pl*AA10 led to the conclusion that the spectral changes are better explained by a coordination change from the suggested bipyramidal (3N2O) coordination environment, to the coordination of a single hydroxyl ion and an intact histidine brace coordination, with distorted square-planar symmetry (3N1O), **Scheme S1**.[15]

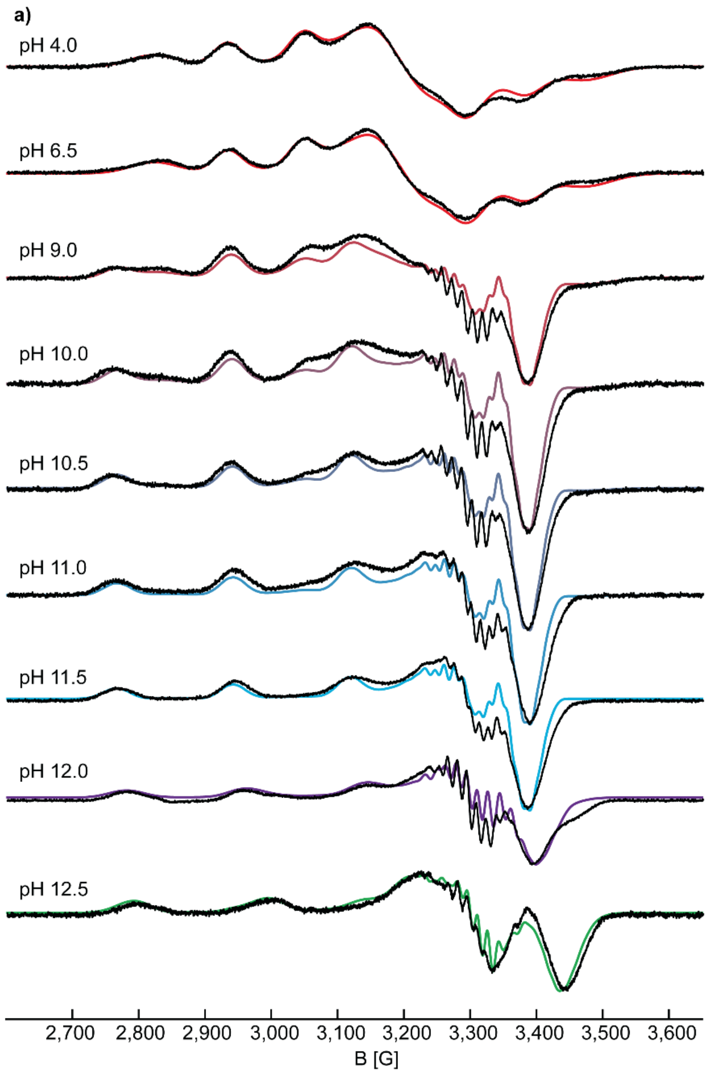

Figure S6. CW X-band (~9.46 GHz) EPR spectra of *Sm*AA10A at various pH values in black with simulations in color.

Table S1. Summary of the EPR parameters for the four species detected by X-band EPR.

	**LPMO-A**	**LPMO-B**	**LPMO-C**	**LPMO-D (case A)** [b)]
**g** = [g_1, g_2, g_3]	[2.258 2.096 2.023]	[2.230 2.051 2.042]	[2.215 2.045 2.039]	[2.179 2.047 2.030]
ΔR[a)]	0.45	0.05	0.04	0.13
**A**(^{63}Cu) = [A_1, A_2, A_3] (MHz)	[350 100 240]	[538 25 99]	[550 60 27]	[605 85 70]
**A**(^{14}N) = [A_1, A_2, A_3] (MHz)	[32.0 44.9 32.2]<br>[32.8 32.5 42.6]<br>[33.9 33.0 43.2]	[35.5 48.0 35.5]<br>[32.0 32.0 42.0]<br>[34.0 34.0 45.0]	[33.0 42.0 36.0]<br>[43.0 45.0 51.0]<br>[46.5 48.5 60.0]	[27.5 37.0 27.0]<br>[40.0 39.0 44.0]<br>[43.0 42.0 52.0]
Linewidth [mT]	2	1.4	1.2	0.9
AStrain [MHz]	[150 120 150]	-	-	-
gStrain [-]	-	-	[0.02 0 0]	[0.01 0 0.04]

a) Rhombicity parameter defined as: $\Delta R = (g_3 - g_2)/(g_2 - g_1)$

b) The simulation of **LPMO-D** in **Figure S6** was obtained with the nitrogen hyperfine interactions of **case A** (see ^{14,15}N ENDOR section). However, both presented cases (**case A** and **case B**) yield nearly identical EPR spectra as shown in **Figure S20**.

Table S2. Relative amounts of the individual species, estimated by X-band EPR simulations.

**Sample**	**Composition**
pH 4.0	100% **LPMO-A**
pH 6.5	100% **LPMO-A**
pH 9.0	50% **LPMO-A**<br>50% **LPMO-B**
pH 10.0	30% **LPMO-A**<br>70% **LPMO-B**
pH 10.5	20% **LPMO-A**<br>80% **LPMO-B**
pH 11.0	10% **LPMO-A**<br>90% **LPMO-B**
pH 11.5	100% **LPMO-B**
pH 12.0	90% **LPMO-C**<br>10% **LPMO-D**
pH 12.5	40% **LPMO-C**<br>60% **LPMO-D**

The spectra of **LPMO-A** (ΔR = 0.45) and **LPMO-B** (ΔR = 0.05) are in fair agreement with the qualitative trends observed for *Pl*AA10 and *Bl*AA10 under neutral and slightly alkaline conditions and show similar degrees of rhombicity. The resolved ^{14}N hyperfine pattern for **LPMO-B** is well reproduced by simulation, that includes three strongly coupled nitrogen nuclei, supporting the integrity of the histidine brace and 3N1O coordination at pH 11.5.

LPMO-C (ΔR = 0.04) and **LPMO-D** (ΔR = 0.13) also exhibit axial EPR spectra, indicating a distorted square-planar symmetry. The gradual reduction of $g_{||}$ and increase of $A_{||}$ going from **LPMO-B** via **LPMO-C** and further to **LPMO-D** (**Table S1**) can be attributed to a stepwise reduction of the overall charge, resulting from deprotonation of the coordinating ligands at increased pH values.[18] For *Bl*AA10 the emergence of a third species at pH 12.5 ($g_{||}$ = 2.180; $A_{||}$ = 614 MHz), for which the deprotonation of the amine function to form the azanido ligand, R-NH^-, was hypothesized,[14] has very similar EPR parameters to what we observe for *Sm*AA10A **LPMO-D** ($g_{||}$ = 2.179; $A_{||}$ = 605 MHz). The spectrum of **LPMO-D** can be reproduced by simulation under consideration of three strongly coupled nitrogen nuclei, which implies an intact histidine brace. However, the EPR spectrum itself and its simulation do not offer sufficient resolution to determine the hyperfine tensors of each coordinating N individually to understand their pH-dependent changes. Thus, further spectroscopic techniques are required to more completely describe the copper coordination sphere at high pH (see ENDOR, ESEEM and HYSCORE sections).

Altogether, four species between pH 6.5 and 12.5 were observed *via* CW X-band EPR, in contrast to the five components assessed with the UV-Vis SVD analysis. A possible reason for this is the small **g**-separation at X-band frequency (~9.5 GHz here), which complicates the disentanglement of several species with similar spin Hamiltonian parameters. Experiments at higher frequency (e.g. Q-band, ~34 GHz) allow for easier differentiation of such species. Indeed, the pulsed Q-band EPR spectra of *Sm*AA10A at pH values between pH 11.5 and pH 12.5 show four, instead of three axial species with similar spin Hamiltonian parameters (see **Figure S7** and **Table S3**), making it a total of five species, in agreement with our findings from UV-Vis SVD analysis. The linewidth observed for the Q-band spectra is relatively large, which is attributed to microwave strain effects, typical for copper centers.[19,20] This broadening precludes the optimal observation of nitrogen hyperfine. Most importantly, the Q-band spectra confirm the general trend of decreasing $g_{||}$ and increasing $A_{||}$ with increasing pH, tentatively attributed to stepwise deprotonation events at the histidine brace.

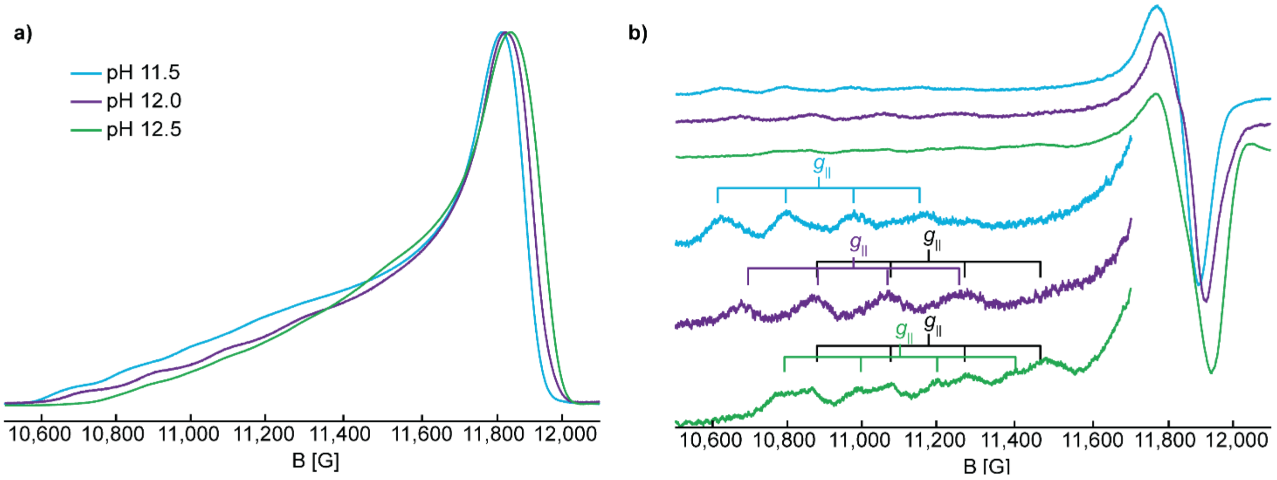

Figure S7. Q-band pulsed EPR spectra of *Sm*AA10A.
(a) As-collected pulsed Q-band EPR spectra of *Sm*AA10A at pH 11.5, 12.0 and 12.5 arbitrarily normalized and **(b)** their derivatives with enlargement of the $g_{\|}$ region with goalposts indicating a total of four species between pH 11.5 and 12.5. $g_{\|}$- and $A_{\|}$-values of the individual components are listed in **Table S3**. Derivatives were obtained by the *fieldmod* function of EasySpin in Matlab with a peak-to-peak modulation of 15 mT. Experimental conditions are reported in the Experimental Section.

Table S3. Spin Hamiltonian parameters for the species observed with Q-band EPR (pH ≥ 11.5). The colors refer to the $A_{\|}$ goalpoasts in **Figure S7b**.

	**Species 1 (blue)**	**Species 2 (purple)**	**Species 3 (black)**	**Species 4 (green)**
$g_{\|}$ [-]	2.230	2.213	2.171	2.185
$A_{\|}$ [MHz]	538	575	610	595

^{1}H Davies and ^{2}H Mims ENDOR Spectroscopy

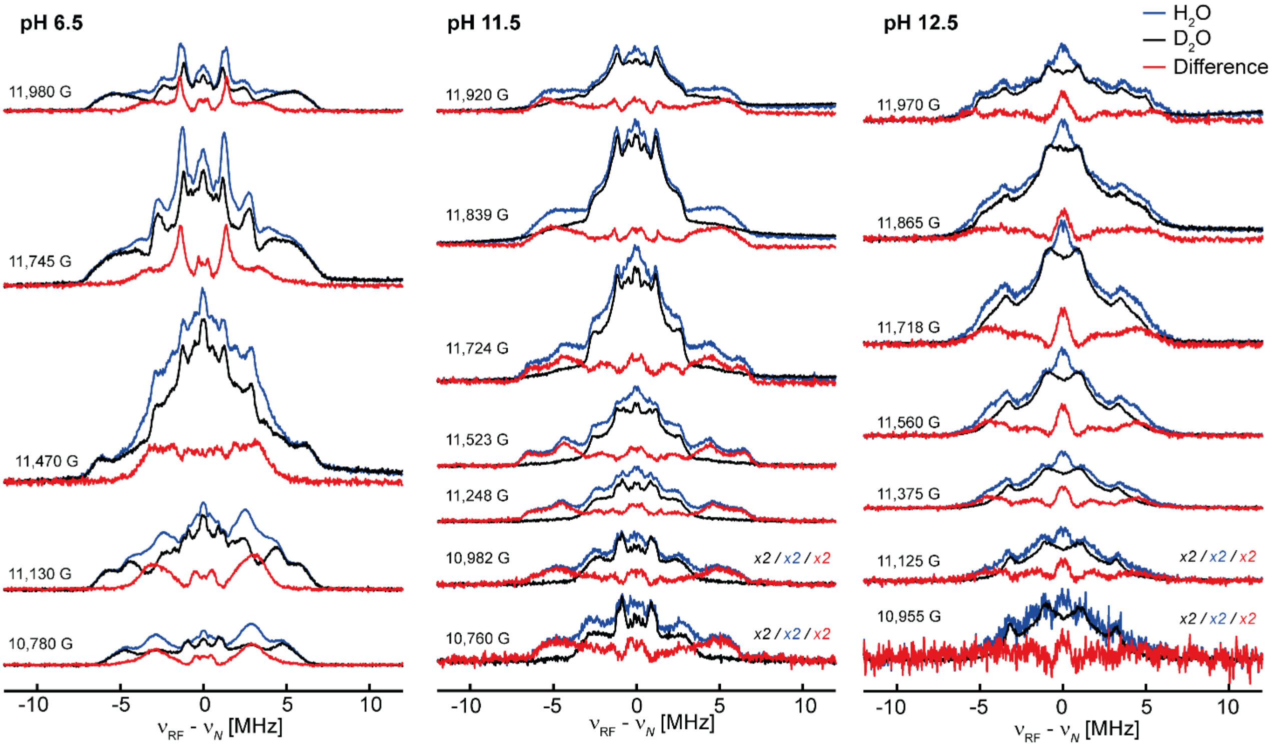

Figure S8. ^{1}H Davies ENDOR spectra.
Q-band ^{1}H Davies ENDOR spectra of *Sm*AA10A at pH 6.5 (left), pH 11.5 (middle) and pH 12.5 (right) in H_2O (blue) and D_2O (black). The D_2O-spectra were subtracted from the H_2O-spectra to depict the exchangeable protons only (red, 'difference spectra'). Experimental conditions are reported in the Experimental section.

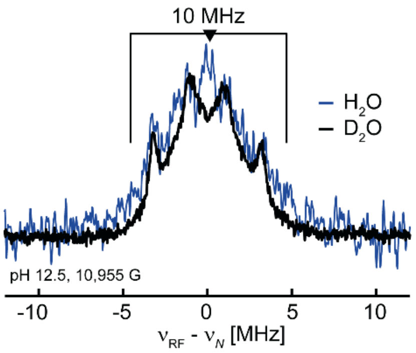

Figure S9. Q-band ^{1}H Davies ENDOR spectra of *Sm*AA10A at pH 12.5.
Q-band ^{1}H Davies ENDOR spectra of *Sm*AA10A at pH 12.5 in H_2O (blue, smoothed over three points) and D_2O (black) approximately at $g_{\|}$ (10.955 G). The goalposts indicate the missing intensities in the D_2O spectrum caused by the exchangeable proton with a coupling of ~ 10 MHz. Experimental conditions are reported in the Experimental section.

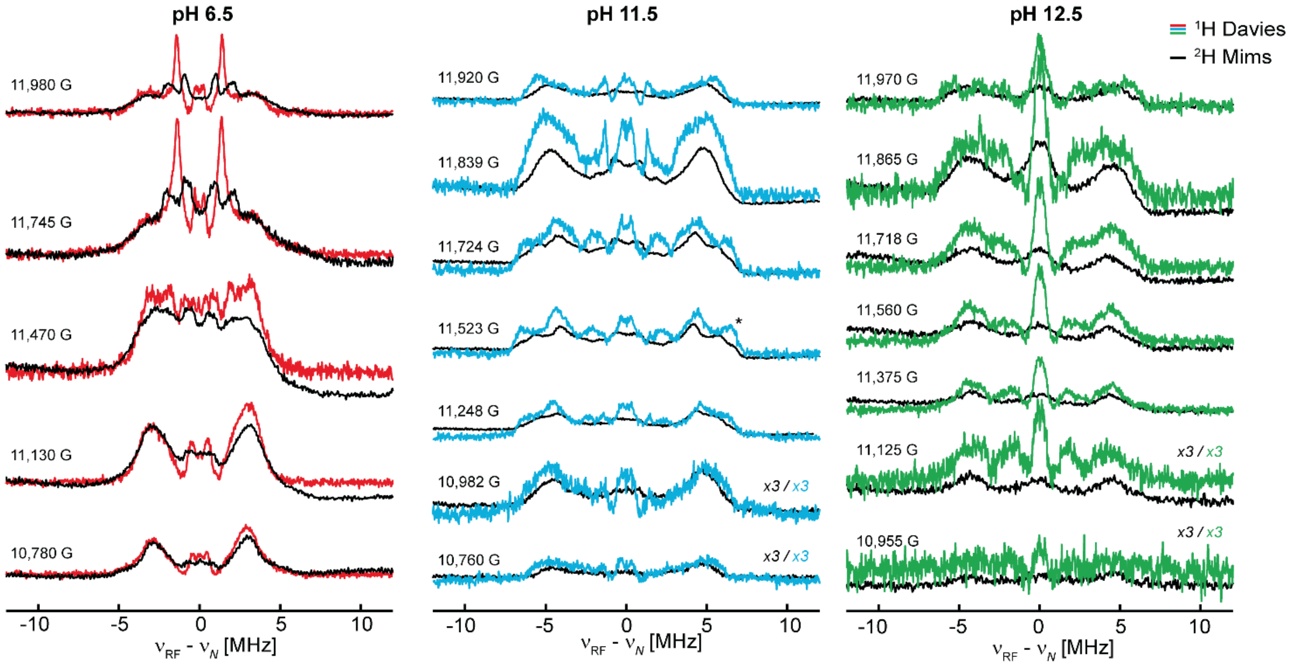

Figure S10. Comparison of Q-band ^{1}H Davies difference and ^{2}H Mims ENDOR spectra.
Q-band ^{1}H Davies ENDOR difference spectra of *Sm*AA10A at pH 6.5 (left), pH 11.5 (middle) and pH 12.5 (right) of the exchangeable protons in color, together with the Q-band ^{2}H Mims ENDOR spectra in black. The x-axis of the ^{2}H Mims spectra is scaled by the gyromagnetic ratio of both nuclei (~6.5) to allow for a direct comparison. Experimental conditions are reported in the Experimental section.

Three-Pulse ESEEM Spectroscopy

Three-pulse ESEEM spectra of *Sm*AA10A at pH 6.5 and 11.5 were collected at multiple field positions and subsequently Fourier transformed (FT, **Figure S11** and **S12**). The spectra were collected at X-band frequency, to match the criterium for the cancellation regime ($2\nu_n \approx a_{iso}$). Inspection of the FT spectra typically allows for precise and accurate estimation of the quadrupole tensor given by e^2qQ/h and η via the sharp and intense features observed at lower frequency (<2 MHz) for imidazoles. Signal intensity related to the hyperfine interaction is observed at higher frequencies centered at approximately $4\times\nu_n$ or in the case of the ideal cancellation regime, $2\times a_{iso}$, near ~ 4 MHz in the FT spectra.

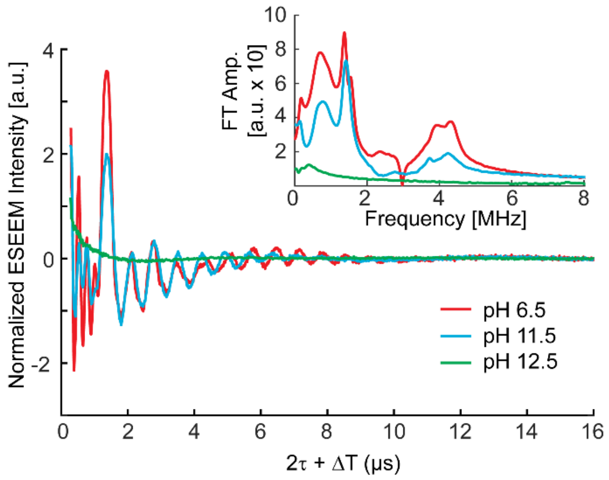

Figure S11. X-band three-pulse ESEEM spectra, normalized by their echo intensity, with their Fourier transformations as inset, recorded for *Sm*AA10A at pH 6.5 (red, 3249 G, 144 ns tau), pH 11.5 (blue, 3340 G, 140 ns tau) and pH 12.5 (green, 3337 G, 140 ns tau), showing the highly similar response of **LPMO-6.5** and **LPMO-11.5**, and the total loss of modulation in **LPMO-12.5**.

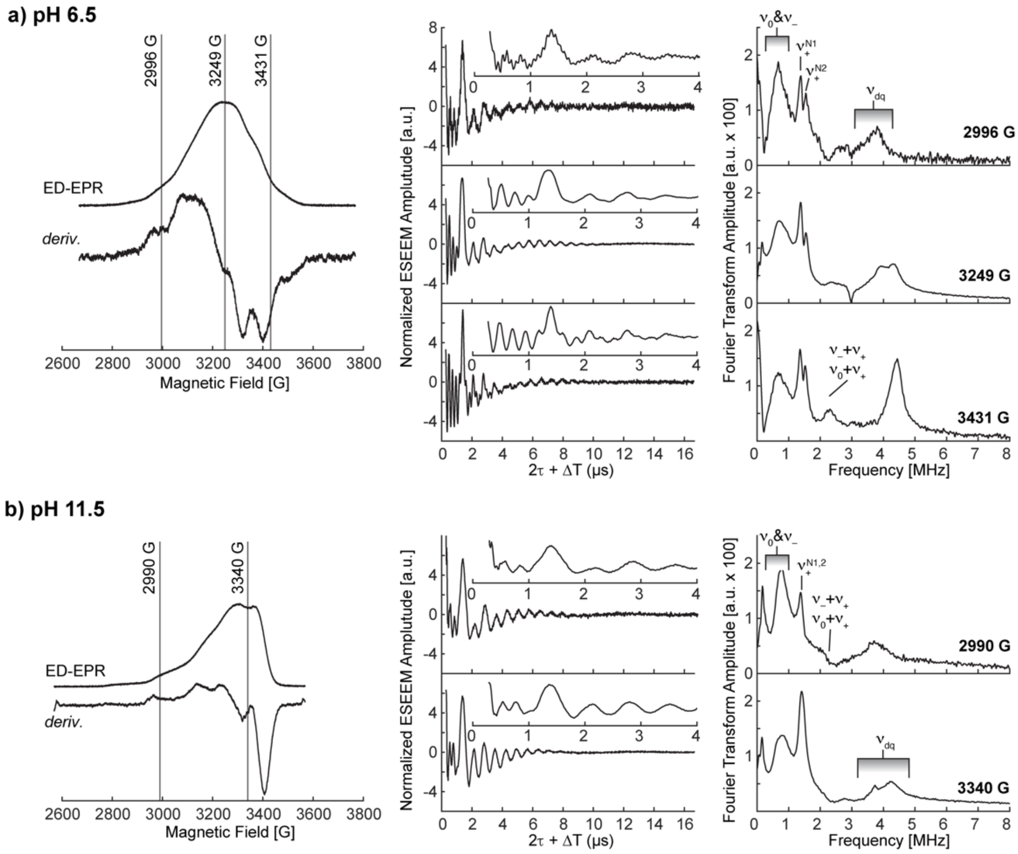

Figure S12. Three-pulse ESEEM spectra of **LPMO-6.5** and **LPMO-11.5**.

Echo-detected EPR (ED-EPR, left) and three-pulse ESEEM spectra of **LPMO-6.5** (**a**) and **LPMO-11.5** (**b**) at several field positions. The as-collected time domain ESEEM spectra are depicted in the middle and the FT spectra on the right. The following τ values were applied: 136 ns (3431 G), 144 ns (3249 G) and 130 ns (2996 G) for **LPMO-6.5**, and 140 ns (3340 G) and 158 ns (2990 G) for **LPMO-11.5**. The τ values were chosen to suppress the ^{1}H response as best as possible. Further experimental conditions are described in the Experimental Section.

For **LPMO-6.5** ESEEM spectra at magnetic fields corresponding to the turning points of its EPR spectrum (g_1, g_2, g_3) were collected. The individual FT spectra generally resemble one another well in the low frequency region (0 to 2 MHz), showing minimal changes in the peak positions and intensities of these quadrupole features, as previously described.[21,22] The higher frequency hyperfine features near ~ 4 MHz, on the other hand, exhibit a clear magnetic field dependence, with the greatest intensity at high-field (g_3) and minimal intensity at low-field (g_1). This double quantum transition ($\Delta m_I = 2$) is labelled as 'ν_{dq}' in **Figure S12**. Both the breadth and the intensity of this feature is influenced by the relative orientation of the quadrupole tensor to the principal axis system.

For **LPMO-11.5**, the three-pulse ESEEM was collected only at two field positions for the axial **g**-tensor ($g_\perp$ and $g_\parallel$) and is also shown in **Figure S12**. Comparing the FT ESEEM spectra of **LPMO-11.5** to **LPMO-6.5**, we see that the low frequency quadrupole feature region resemble one another, suggesting that the quadrupole

values are in the same range for both samples. However, this comparison now highlights the subtle but important splitting of the ν_+ feature in **LPMO-6.5**. This splitting indicates some inequivalences between the two histidine ligands in **LPMO-6.5** that is not readily observed in **LPMO-11.5**. A broader shoulder is observed at ~ 2 MHz in **LPMO-11.5** at 2990 G from the overlapping combination peaks. The double quantum feature centered near 4 MHz exhibits similar breadth to the g_1 (2996 G) ESEEM spectrum of **LPMO-6.5**, but never gains the same remarkable intensity observed at high-field for **LPMO-6.5**. This lack of intensity of the double quantum feature of **LPMO-11.5** at high-field is due to the axial nature of the **g**-tensor and that effective two molecule orientations (x and y) are observed at this field position as opposed to the single molecular orientation of the rhombic **LPMO-6.5** sample.

To resolve the spin Hamiltonian parameters of the remote imidazole nitrogens, fitting of the multi-field ESEEM spectra was completed with a custom routine using the saffron function of EasySpin. The three-pulse ESEEM spectra were fit via a custom Matlab routine utilizing the *saffron* function[23] of EasySpin to generate calculated time-domain spectra. In short, multiple field positions may be simultaneously fit with equal weighting via their normalized time domain spectra (mean set to 0 and single standard deviation equal to 1), to a single set of parameters. A single set of spin Hamiltonian parameters was used to simulate the time-domain spectra of all field positions. The calculated spectrum at each field position was matched to the experimental spectrum through a two-step fitting routine. First, the calculated spectrum was scaled, offset, and fit to exponential decay function (baseline) via the non-linear least squares fitting of a single matching function:

$$Y_{m1} = a * (Y_{calc} + b) * e^{ct} + d.$$

The second matching function applied an exponential dampening to the adjusted calculated data from the first step (now Y_{m1}), to emulate the effects T_2 relaxation, to yield the final matched spectrum, Y_{m2}. The goodness-of-fit was evaluated via:

$$GOF = \sum_{k} \sum_{i}^{n_k} \left(Y_i^{obs} - Y_i^{m2}\right)^2 / L_k$$

where i is each data point for each spectrum, having a total length of L_k, summing over each spectrum k. The spin Hamiltonian parameters were optimized via a genetic algorithm routine, with random start points. To increase both the speed of fitting, and allow for a large initial population (≥ 800), the fitting routine was written utilizing Matlab's Optimization Toolbox with automatic parallel programming support, allowing the fits to be ran on a high-performance computing cluster. Confidence intervals (CI) for the fitted parameters were estimated following previously described methods[24], where the variances of the measurement error are assumed to follow a normal distribution, allowing for the uncertainty of each fitted parameter with respect to the other fitted parameters to be determined.

The nitrogen spin Hamiltonian parameters that can be fit include: the hyperfine tensor (**A** = [A_1, A_2, A_3]), quadrupole coupling constant (e^2qQ/h) and the quadrupole tensor's rhombicity (η). Additionally, the relative

tensor orientations to the molecular frame (or another tensor) may also be fitted through a set of Euler angles α, β, γ applied in the zxz' convention. Three different simulation models were tested and evaluated for their ability to best reproduce the data. The parameters obtained from the fits are reported in **Table S4** and **S5** together with the 95% confidence intervals calculated in a similar matter as previously described.[24] The fit models are briefly described below:

Model A: The ^{14}N ESEEM is modeled with two equivalent ^{14}N nuclei that are allowed to deviate only in their relative orientations. The principle hyperfine values (A_1, A_2, A_3), as well as the nuclear quadrupole parameters e^2qQ/h and η for each nucleus (N1 and N2) is equal to one another. In the principle axis system (PAS), the hyperfine tensor of N1 is colinear with **g**, and the quadrupole tensor of N1 is rotated freely from A(N1) by the Euler angles: α, β, γ. N2's **A-** and **P**-tensors have the same applied Euler rotation as N1, making the two nitrogen nuclei equivalent. However, the orientation of N2 relative to N1 is allowed to also refine through the applied Euler angles of α', β', γ' applied to the molecular frame of N2. The total number of parameters is 11.

Model B: The ^{14}N ESEEM is modeled with two inequivalent ^{14}N nuclei, where the principle hyperfine values (A_1, A_2, A_3), and the quadrupole parameters e^2qQ/h and η for each nucleus (N1 and N2) is allowed to individually refine. Each **A**-tensor is assumed to be collinear to the **g**-tensor, but the relative orientations of each nitrogen **P**-tensor to **g** (and **A**) are optimized by fitting their independent Euler rotation angles α, β, γ. The total number of parameters is 16.

Model C: The ^{14}N ESEEM is modeled with two inequivalent ^{14}N nuclei that are allowed to freely refine independent of each other, including their individual **A** and **P** Euler rotations. This model imposes no restrictions, representing the largest number of parameters (22).

Each model was tested on the multi-field data of **LPMO-6.5** with random starting points and employed a genetic fitting algorithm (minimum 400 generations) as described in the Experimental Section. The final fits of each model are shown in **Figure S13**. The fitting parameters and the root-mean-square deviation (RMSE) for each model's time domain and FT spectra are reported in **Table S4.** Model A is the most constrained model with the least number of parameters. This model makes the assumption that the magnetic and electronic properties of the two remote imidazole nitrogens are the same, encoded in identical **A-** and **P**-tensors (including the relative orientation of these two interactions). This approach has proven successful for other bis-histidine complexes and copper proteins, where excellent reproduction of the ESEEM data was achieved.[24,25]

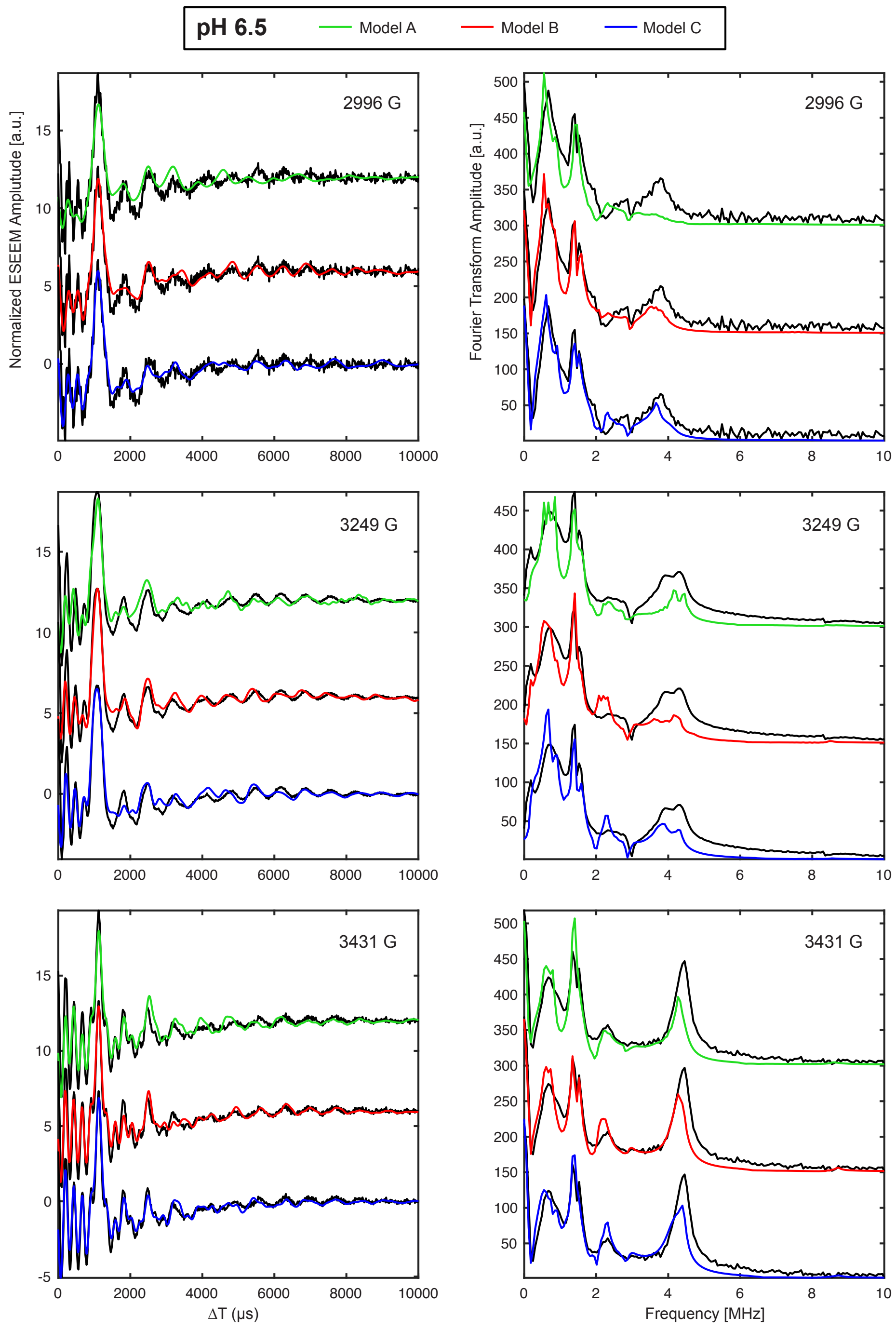

Figure S13. Three-pulse ESEEM fittings for **LPMO-6.5**.

Fittings of the three-pulse ESEEM spectra of **LPMO-6.5** at 2996 G (top), 3249 G (middle) and 3431 G (bottom) in black with simulations in green (Model A), red (Model B) and blue (Model C). The time-domain spectra are depicted on the left and the FT spectra on the right. The following τ values were applied: 130 ns (2996 G), 144 ns (3249 G) and 136 ns (3431 G). Further experimental conditions and the fitting procedure are described in the Experimental Section.

Table S4. Nitrogen spin Hamiltonian parameters from fitted three-pulse ESEEM data of **LPMO-6.5**.

**LPMO-6.5**		Model A			Model B			Model C		
		Fit	CI (95%) –	CI (95%) +	Fit	CI (95%) –	CI (95%) +	Fit	CI (95%) –	CI (95%) +
N1	A_1 (MHz)	1.968	*1.922*	*1.991*	1.959	*1.900*	*1.964*	1.548	*1.541*	*1.553*
	A_2 (MHz)	2.373	*2.322*	*2.392*	2.523	*2.452*	*2.528*	1.727	*1.700*	*1.737*
	A_3 (MHz)	1.141	*1.097*	*1.194*	1.315	*1.272*	*1.335*	2.099	*2.097*	*2.104*
	a_{iso} (MHz)[a)]	1.827			1.932			1.792		
	t (MHz)[b)]	0.273			0.295			0.154		
	e^2qQ/h (MHz)	1.419	*1.412*	*1.426*	1.359	*1.357*	*1.361*	1.393	*1.390*	*1.395*
	η	0.834	*0.820*	*0.844*	0.914	*0.901*	*0.917*	0.937	*0.934*	*0.941*
	α(**A**)°							9.5	*9.0*	*9.7*
	β(**A**)°							75.7	*74.8*	*77.9*
	γ(**A**)°							27.3	*25.6*	*27.9*
	α(**Q**)°	17.9			114.2	*110.8*	*118.9*	86.1	*85.1*	*90.4*
	β(**Q**)°	14.0			53.8	*50.8*	*55.3*	50.3	*47.1*	*50.5*
	γ(**Q**)°	134.7			56.8	*55.2*	*57.2*	61.1	*59.7*	*62.3*
N2	A_1 (MHz)				2.073	*2.048*	*2.079*	1.651	*1.632*	*1.684*
	A_2 (MHz)				1.501	*1.448*	*1.530*	1.269	*1.248*	*1.295*
	A_3 (MHz)				1.307	*1.286*	*1.344*	2.118	*2.097*	*2.140*
	a_{iso} (MHz)[a)]				1.627			1.679		
	t (MHz)[b)]				0.223			0.219		
	e^2qQ/h (MHz)				1.498	*1.495*	*1.503*	1.531	*1.527*	*1.536*
	η				0.965	*0.963*	*0.981*	0.729	*0.720*	*0.732*
	α(**A**)°	148.3[c)]						-11.9	*-12.5*	*-11.2*
	β(**A**)°	38.8[c)]						-63.2	*-65.0*	*-60.0*
	γ(**A**)°	61.9[c)]						-138.3	*-142.5*	*-134.2*
	α(**Q**)°				-135.5	*-139.1*	*-132.4*	50.0	*47.5*	*52.3*
	β(**Q**)°				24.8	*23.5*	*25.3*	-47.3	*-49.5*	*-46.5*
	γ(**Q**)°				-28.4	*-31.0*	*-26.2*	124.2	*120.8*	*127.4*
RMSE (time domain)		0.9387			0.5994			0.5381		
RMSE (FT)		470.6			359.3			326.8		

a) Isotropic hyperfine coupling, a_{iso}, calculated from fitted hyperfine values. $a_{iso} = (A_1 + A_2 + A_3)/3$

b) Anisotropic hyperfine coupling, t, calculated from fitted hyperfine values, assuming axial tensor shape **T**. $t = (A_{max} - a_{iso})/2$

c) N2 of Model A represents an identical ^{14}N nucleus to N1, where the reported Euler angles represent the relative orientations of the two nitrogens.

The fits of Model A (**Figure S13**) overall reproduce the data, yielding a good starting point for the estimation of the nitrogens' spin Hamiltonian parameters. The model partially reproduces the high-frequency features of the time domain spectra, seen at low ΔT, but with variable success in the intensity. This translates to a clear mismatch of the intensity of the double quantum feature across the three magnetic field positions, with the lowest field position having the least intensity in the simulation and largest difference with respect to the experiment. The simulation of Model A also reproduces the general shape of the quadrupole features observed

in the FT spectra, with a broad feature centered around 0.9 MHz and a sharper feature at ~1.5 MHz. The sharp signal at 1.5 MHz is the ν_+ feature of the two equivalent quadrupole interactions. It is quite clear that this model is unable to reproduce the splitting of the ν_+ feature observed in the experimental data, requiring an expanded model to fit the interaction of two nitrogens with inequivalent quadrupole couplings.

The fitting results of Model B represent a significant improvement compared to Model A, statistically shown by the large reduction of the goodness-of-fit value (RMSE, **Table S4**) for both the time domain spectra and the corresponding FT spectra. Model B also faithfully reproduces the ν_+ features of the quadrupole interaction as the two nitrogens refine to different values of N1: e^2qQ/h = 1.359 MHz, η = 0.914 and N2: e^2qQ/h = 1.498 MHz, η = 0.965. Both the fitted e^2qQ/h and η values are in the ranges typically observed for the remote nitrogens of imidazole rings[21,22,25–27] and match the previously established correlations where larger quadrupole interactions are associated with lower rhombicity.[26,28] The corresponding ν_- and ν_0 transitions of the quadrupole interaction are observed at lower frequencies, around 0.6 to 0.8 MHz, as expected for systems near the cancelation regime. However, the individual transitions are not well resolved precluding individual assignments. At the rhombic limit of η = 1, the ν_- and ν_0 transitions would overlap to form a single sharp transition around 0.7 MHz.[29] However, the fitted η values do a good job of reproducing the broadness and shape of this low frequency transition.

In this model, the two hyperfine tensors of the two nitrogen nuclei are allowed to independently refine, and have best-fits of **A**(N1) = [1.959, 2.523, 1.315] MHz and **A**(N2) = [2.073, 1.501, 1.307] MHz, which have isotropic (a_{iso}) and dipolar (t) components of a_{iso}(N1) = 1.932MHz, t(N1) = 0.295 MHz and a_{iso}(N2) = 1.627 MHz, t(N2) = 0.223 MHz. The isotropic component is typical of other copper histidine proteins[26], with one nitrogen exhibiting a slightly larger anisotropy than the other. Furthermore, the differing hyperfine tensors, and their anisotropic components contribute to the broadening observed in the double quantum feature, particularly at lower field.

Model C represents the best fit to the ESEEM data of **LPMO-6.5**, judged by the lowest RMSE value. However, this comes along with the cost of using significantly more parameters. Inspection of the reduced χ^2 values for the time-domain fits (χ^2-A = 0.5961; χ^2-B = 0.4580; χ^2-C = 0.4188), which is statically normalized for the degrees of freedom in the fit, still shows that Model C is a significant improvement over A, but perhaps only a modest improvement over B. Model C does the best job of fitting the ν_{dq} features at low and mid magnetic fields, however Model B perhaps fits this region best at the highest field position. Both Models B and C have fits with similar hyperfine tensors, where their determined a_{iso} and t values are similar (**Table S4**). Model C exhibits a slightly larger separation of the e^2qQ/h values for its two nitrogen centers, however, this still appears within range of Model B and reproduces the data very well.

To test the robustness of the fit, 95% confidence intervals of each parameter were calculated and are listed in **Table S4**. The confidence intervals are relatively small, and fitted parameters such as e^2qQ/h and η do not overlap from one nuclei to the other. Lastly, to understand and better visualize the global minima found with

two distinct quadrupole couplings, the surface plot of a two-dimensional scan the RMSE values as a function of the N1 and N2's e^2qQ/h values is shown in **Figure S14**. A clear and deep global minimum is found with a shoulder, where the deepest part corresponds to the fitted e^2qQ/h values. The shoulder in this valley is simply the 'exchange' of the two nitrogen's e^2qQ/h values, without exchanging their fitted η parameters. Given the closeness of the fitted η values, it is easy to imagine swapping of the two nitrogens in the model to realize a similar fit. However, this RMSE scan does show and support that two unique e^2qQ/h values are observed for **LPMO-6.5**.

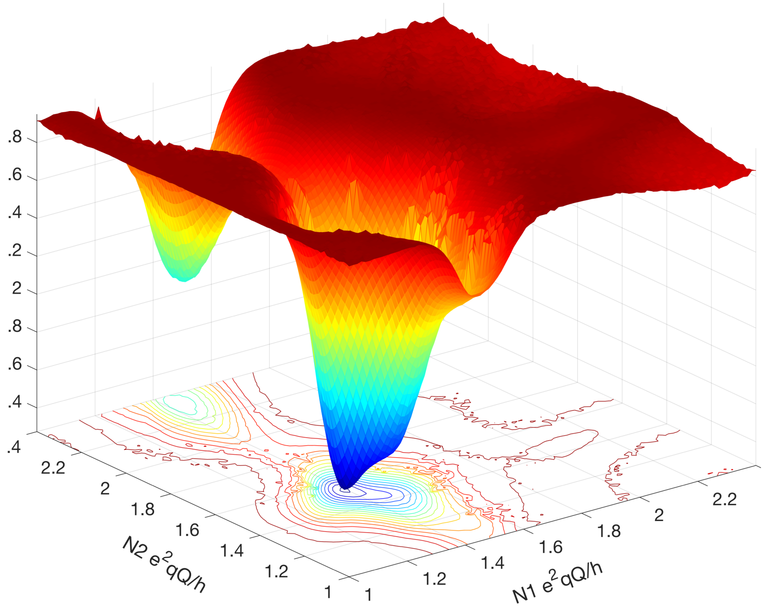

Figure S14. RMSE surface plot as a function of the two-dimensional scan of the e^2qQ/h values of N1 and N2 for **LPMO-6.5** in Model C**.**

Similar RMSE surface plots of e^2qQ/h vs. η for only N1 or N2 are depicted in **Figure S15**. For N2, a clear and single global minimum is observed. However, for N1 a deep local minimum near e^2qQ/h ~ 1.8 MHz and η ~ 0.3 is found. The multiple local minima of this error analysis emphasize the utility of large-scale fitting methods such as genetic algorithms to accurately find a global minimum.

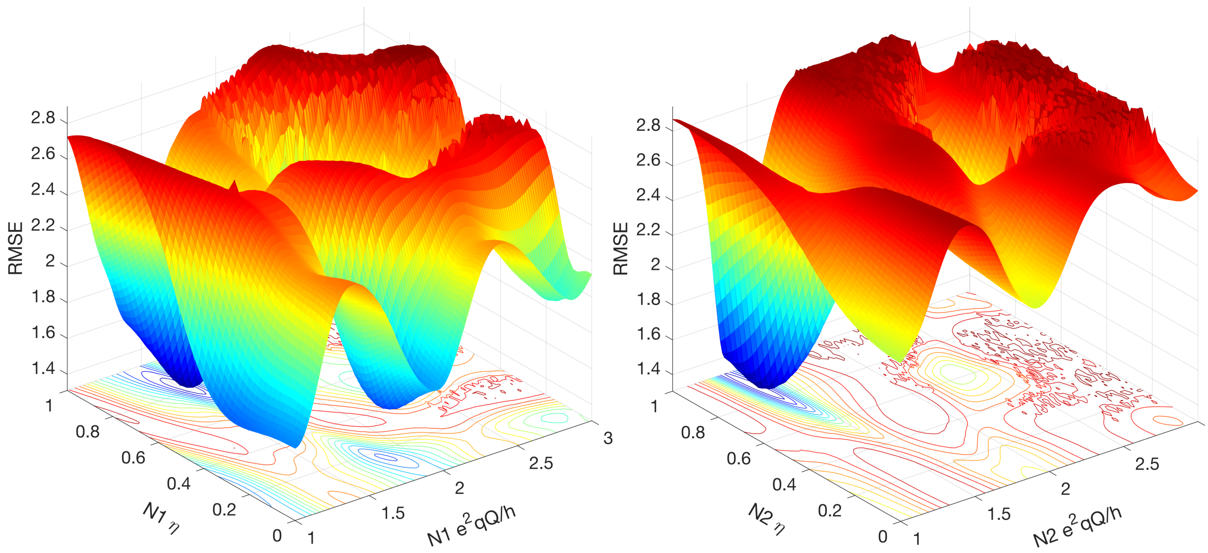

Figure S15. RMSE surface plot as a function of the two-dimensional scan of the e^2qQ/h vs. η values for N1 (left) and N2 (right).

Fitting of a recently obtained HYSCORE spectrum of *Sm*AA10A by Munzone et al. yielded quadrupole coupling values *K* of 1.06 MHz and 1.05 MHz and lower asymmetry with $\eta = 0.64$ and 0.36, respectively.[10] *K* relates to e^2qQ/h via:

$$K = \frac{e^2qQ/h}{4I(2I-1)}$$

$$P = \frac{e^2qQ/h}{4I(2I-1)}\begin{bmatrix} -(1-\eta) & 0 & 0 \\ 0 & -(1+\eta) & 0 \\ 0 & 0 & 2 \end{bmatrix} = \begin{bmatrix} -(1-\eta)K & 0 & 0 \\ 0 & -(1+\eta)K & 0 \\ 0 & 0 & 2K \end{bmatrix}$$

For the ^{14}N ($I = 1$) nucleus this results in a factor of 4, which would make their reported *K* values a corresponding $e^2qQ_{zz}/h > 4$ MHz. This is an atypically large coupling for copper coordinated imidazoles[26,28], and generally most nitrogen quadrupole couplings. More likely, their *K* is differs from e^2qQ/h by a factor of two. Even taking a e^2qQ/h of ~2.1 MHz (2*K*) from Munzone et al., the expected quadrupole transitions for N1 are: $\nu_+ = 1.91$ MHz, $\nu_- = 1.24$ MHz, $\nu_0 = 0.66$ MHz and for N2: $\nu_+ = 1.76$ MHz, $\nu_- = 1.39$ MHz, $\nu_0 = 0.36$ MHz. However, the feature expected at higher frequency (1.91 MHz) is not observed in our data. We must emphasize that the quadrupole interaction is more directly observed and ‘readable’ in three-pulse ESEEM compared to HYSCORE spectroscopy, and our multi-field measurements offer both consistent and reliable measurements of the quadrupole interaction. Interestingly, the values of $e^2qQ/h \sim 2$ MHz and $\eta = 0.34$ is near the local minimum we found in **Figure S15**, but differs from the global minimum.

To fully simulate the previously published single field HYSCORE spectrum[10], a third weakly coupled nitrogen nucleus was included, that appears qualitatively similar to what we have identified in our ^{15}N HYSCORE (**Figure 4c**). This highlights the strength of using three-pulse ESEEM to measure the histidine’s

remote nitrogen nuclei, which dominate the ESEEM spectrum. The third weakly coupled nitrogen does not exhibit appreciable modulation depth as it deviates significantly from the cancellation regime, yielding in a selectivity that helps to simplify the analysis. Beyond the additional difficulties and errors associated with HYSCORE simulations, the reported e^2qQ/h and η values from Munzone et al. are inconsistent for imidazole couplings. The above-mentioned reciprocal relationship between the magnitude and rhombicity of nuclear quadrupole couplings should have a η of near 0.35 for a e^2qQ/h of 2 MHz, agreeing with the parameters of only one of the nitrogen nuclei.

The different ESEEM models are also tested for **LPMO-11.5** (**Figure S16**). All of the models reproduce the quadrupole features at lower frequency with good satisfaction. Once again, Model A fails to reproduce the intensity of the ν_{dq} feature at the low magnetic field position ($g_{\parallel}$). Models B and C both reproduce the combination peaks in the FT spectra well, seen at 2 MHz as a low intensity shoulder of the sharp ν_{+} quadrupole transition. Statistically, Model B is an improved fit over Model A (**Table S5**). However, Model C does not offer significant improvement over Model B. This may be in part due to the additional orientation selection available for the rhombic **LPMO-6.5** sample which therefore offers the higher resolution data. The fitted parameters of Model B and C are quite similar **LPMO-11.5** but for simplicity we will limit our discussion to the fits from Model C for comparisons between samples.

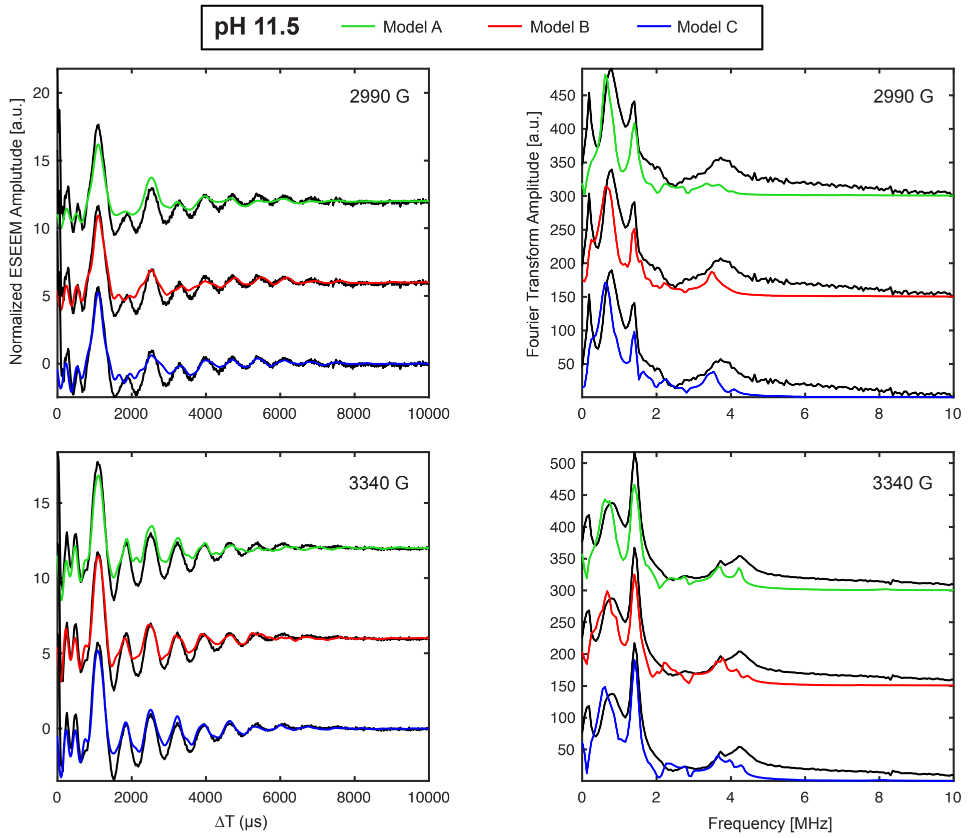

Figure S16. Three-pulse ESEEM fittings for **LPMO-11.5**.

Fittings of the three-pulse ESEEM spectra of **LPMO-11.5** at 2990 G (top) and 3430 G (bottom) in black with simulations in green (Model A), red (Model B) and blue (Model C). The time-domain spectra are depicted on

the left and the FT spectra on the right. The following τ values were applied: 158 ns (2990 G) and 140 ns (3340 G). Further experimental conditions and the fitting procedure are described in the Experimental Section.

The three-pulse ESEEM of **LPMO-11.5** (Model C) reveals that the e^2qQ/h values of the two nitrogens become more similar (1.374 and 1.438 MHz; Δ = 0.064 MHz) than what was observed for **LPMO-6.5**, while the rhombicities now differ more (η = 0.937 and 0.790) (**Table S5**). The RMSE error analysis of these fits shows that global minima are reached and the two-dimensional scan of N1's vs. N2's e^2qQ/h values does not show a pseudo double well like **LPMO-6.5**, but a single well because the quadrupole constants are so close in value (**Figure S17, Table S5**). Furthermore, the fitted hyperfine parameters result in a slightly lower isotropic coupling of the two remote nitrogens, a_{iso} = 1.65 and 1.51 MHz, compared to the larger 1.79 and 1.95 MHz coupling of **LPMO-6.5**. The change of the remote nitrogens' hyperfine is perhaps a bit surprising considering there is no significant change of the hyperfine interaction of the strongly coupled, coordinating nitrogen nuclei (as measured by ^{14,15}N ENDOR see main text, **Table S6**). This suggests that other effects may be at play, such as a change in the H-bonding networks of the imidazole's remote nitrogen nuclei upon the pH change, but this proposal would require further investigations.

Table S5. Nitrogen spin Hamiltonian parameters from fitted three-pulse ESEEM data of **LPMO-11.5**.

**LPMO-11.5**		Model A			Model B			Model C		
		Fit	CI (95%)		Fit	CI (95%)		Fit	CI (95%)	
			–	+		–	+		–	+
N1	A_1 (MHz)	1.970	*1.896*	*2.052*	1.360	*1.300*	*1.423*	1.450	*1.413*	*1.488*
	A_2 (MHz)	1.438	*1.382*	*1.480*	1.667	*1.595*	*1.742*	2.033	*1.924*	*2.153*
	A_3 (MHz)	1.266	*1.209*	*1.321*	1.448	*1.395*	*1.493*	1.454	*1.396*	*1.518*
	a_{iso} (MHz)[a)]	1.558			1.492			1.646		
	t (MHz)[b)]	0.206			0.088			0.194		
	e^2qQ/h (MHz)	1.379	*1.367*	*1.384*	1.438	*1.417*	*1.454*	1.374	*1.364*	*1.381*
	η	0.906	*0.889*	*0.920*	0.764	*0.742*	*0.781*	0.937	*0.919*	*0.960*
	α(**A**)°							105.1	*97.4*	*111.7*
	β(**A**)°							69.2	*52.5*	*81.2*
	γ(**A**)°							148.7	*141.6*	*153.6*
	α(**Q**)°	92.8			79.8	*68.5*	*84.6*	82.1	*75.1*	*92.1*
	β(**Q**)°	79.5			38.5	*28.1*	*42.8*	49.6	*46.1*	*55.4*
	γ(**Q**)°	102.3			68.5	*58.5*	*75.5*	96.3	*88.9*	*102.7*
N2	A_1 (MHz)				1.952	*1.862*	*2.069*	1.749	*1.679*	*1.809*
	A_2 (MHz)				1.500	*1.428*	*1.567*	1.337	*1.244*	*1.398*
	A_3 (MHz)				1.494	*1.455*	*1.536*	1.441	*1.381*	*1.502*
	a_{iso} (MHz)[a)]				1.649			1.509		
	t (MHz)[b)]				0.152			0.120		
	e^2qQ/h (MHz)				1.375	*1.369*	*1.382*	1.438	*1.417*	*1.454*
	η				0.953	*0.935*	*0.968*	0.790	*0.770*	*0.811*
	α(**A**)°	53.7[c)]						-10.2	*-17.5*	*-1.0*
	β(**A**)°	37.5[c)]						-28.6	*-37.4*	*-20.1*
	γ(**A**)°	0.9[c)]						-44.1	*-52.4*	*-33.3*
	α(**Q**)°				-127.4	*-134.0*	*-119.3*	74.6	*67.7*	*83.2*
	β(**Q**)°				62.3	*52.7*	*68.6*	-46.0	*-52.0*	*-38.9*
	γ(**Q**)°				104.0	*95.4*	*112.9*	-78.4	*-90.4*	*-70.1*
	RMSE (time domain)	0.9387			0.5994			0.5381		
	RMSE (FT)	470.6			359.3			326.8		

a) Isotropic hyperfine coupling, a_{iso}, calculated from fitted hyperfine values. $a_{iso} = (A_1 + A_2 + A_3)/3$

b) Anisotropic hyperfine coupling, t, calculated from fitted hyperfine values, assuming axial tensor shape **T**. $t = (A_{max} - a_{iso})/2$

c) N2 of Model A represents an identical ^{14}N nucleus to N1, where the reported Euler angles represent the relative orientations of the two nitrogens.

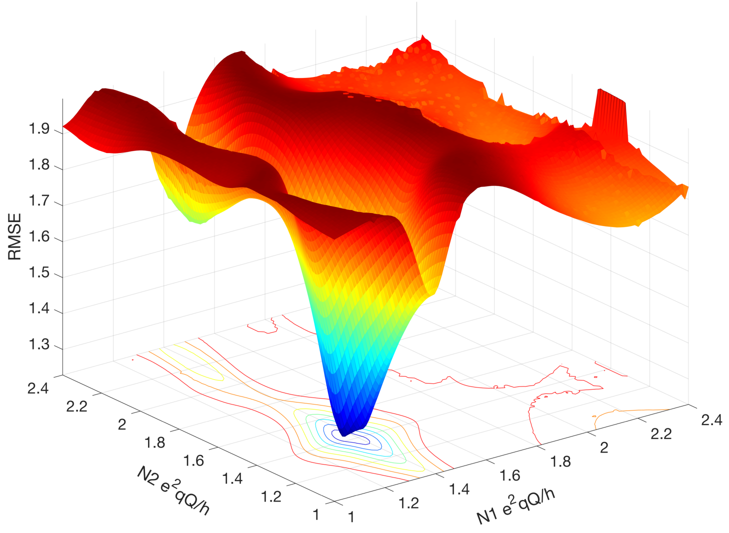

Figure S17. RMSE surface plot as a function of the two-dimensional scan of the e^2qQ/h values of N1 and N2 for **LPMO-11.5**.

^{15}N HYSCORE Spectroscopy

To further understand the change in the electronic structure of the coordinating imidazoles of the histidine brace upon deprotonation, we conducted HYSCORE experiments on globally ^{15}N-enriched samples (denoted as **^{15}N-LPMO-6.5**, **^{15}N-LPMO-11.5** and **^{15}N-LPMO-12.5**), which only have a hyperfine interaction, without the additional quadrupole splitting, simplifying the interpretation of the spectra. The HYSCORE spectra for all three samples are presented in **Figure 4c**, where the (+,+) quadrant contains frequency responses in the weak coupling regime ($|a_{iso}| < 2|\nu_N|$), while strongly coupled nuclei ($|a_{iso}| > 2|\nu_N|$) appear in the (-,+) quadrant. When the hyperfine couplings are near the cancellation regime ($2\nu_n \approx a_{iso}$), as shown in the ^{14}N ESEEM data for **LPMO-6.5** and **LPMO-11.5**, transitions are observed in both quadrants.

Indeed, for **^{15}N-LPMO-6.5** and **^{15}N-LPMO-11.5** transitions in both quadrants can be observed, in the form of large cross coupling peaks at (-0.5,+2.5) and (-2.5,0.5) in the (–,+) quadrant and the corresponding signals in the (+,+) quadrant, indicating hyperfine couplings of $A(^{15}N)$ ~ 2 MHz (equivalent to $A(^{14}N)$ ~1.4 MHz). This shows that $a_{iso}(^{14,15}N)$ is approximately equal to the Larmor frequency for these two samples and further validates the observation of strong three-pulse ESEEM modulations in **LPMO-6.5** and **LPMO-11.5**.

For **^{15}N-LPMO-12.5**, on the other hand, the coupling signals are not present, **Figure 4c**. Furthermore, no other strong couplings in the (-,+) quadrant are observed for this sample, indicating that at pH 12.5 the a_{iso} is smaller than the Larmor frequency. Considering the absence of such strongly coupled signals in the HYSCORE and the lack of ^{14}N modulation in the three pulse ESEEM experiment for **LPMO-12.5**, we propose that the isotropic coupling of the remote nitrogens significantly decreases upon deprotonation.

In all of the samples, a broad signal on the off diagonals of the ^{15}N Larmor frequency is observed in the (+,+) quadrant. Since these weakly coupled interactions are observed in all three samples, they likely have origins other than the remote nitrogen nuclei of the coordinating imidazole rings, such other distant ^{15}N labeled amino acids.[10] Overall, the ESEEM and HYSCORE experiments support the deprotonation of the remote nitrogen nuclei in **LPMO-12.5**.

The HYSCORE signals of the stronger coupled ^{15}N signals in **LPMO-6.5** and **LPMO-11.5**, are well simulated (**Figure S18**) by employing the fitted and scaled ^{14}N hyperfine parameters from the ^{14}N ESEEM fits. Both of these ^{15}N HYSCORE simulations reproduce the cross peaks in the (–,+) quadrant at (± 0.5, ∓ 2.5) and the general shape of the signal. Additionally, the largest coupling cross peaks of the (+,+) quadrant in **Figure 18**, are also well reproduced. The broader, weaker coupling centered near the ^{15}N Larmor frequency in the (+,+) quadrant was not simulated in **LPMO-6.5** and **11.5**. However, simulation with a generally dipolar ^{15}N hyperfine tensor, **A** = [-0.3, -0.3, 0.9] MHz, reproduces the overall breadth of the Larmor centered broad feature in the **LPMO-12.5** sample. The weak coupling and broad shape of the signal is consistent with distant and possibly numerous disordered ^{15}N nuclei contributions. The skyline projections of the HYSCORE spectra show that this feature is over 1.5 MHz wide. We also note that the simulations of **LPMO-6.5** and **LPMO-11.5** do not

reproduce the features at (-1.3, 0.9; 1.3, -0.9) MHz in the (-,+) quadrant. Although these features are not clearly apparent in the contour plot of **LPMO-12.5**, an intensity profile in the skyline projection is observed that resembles their character seen in **LPMO-6.5** and **LPMO-11.5**. From this, we conclude that these features do in fact belong to the weak coupled distant ^{15}N nuclei.

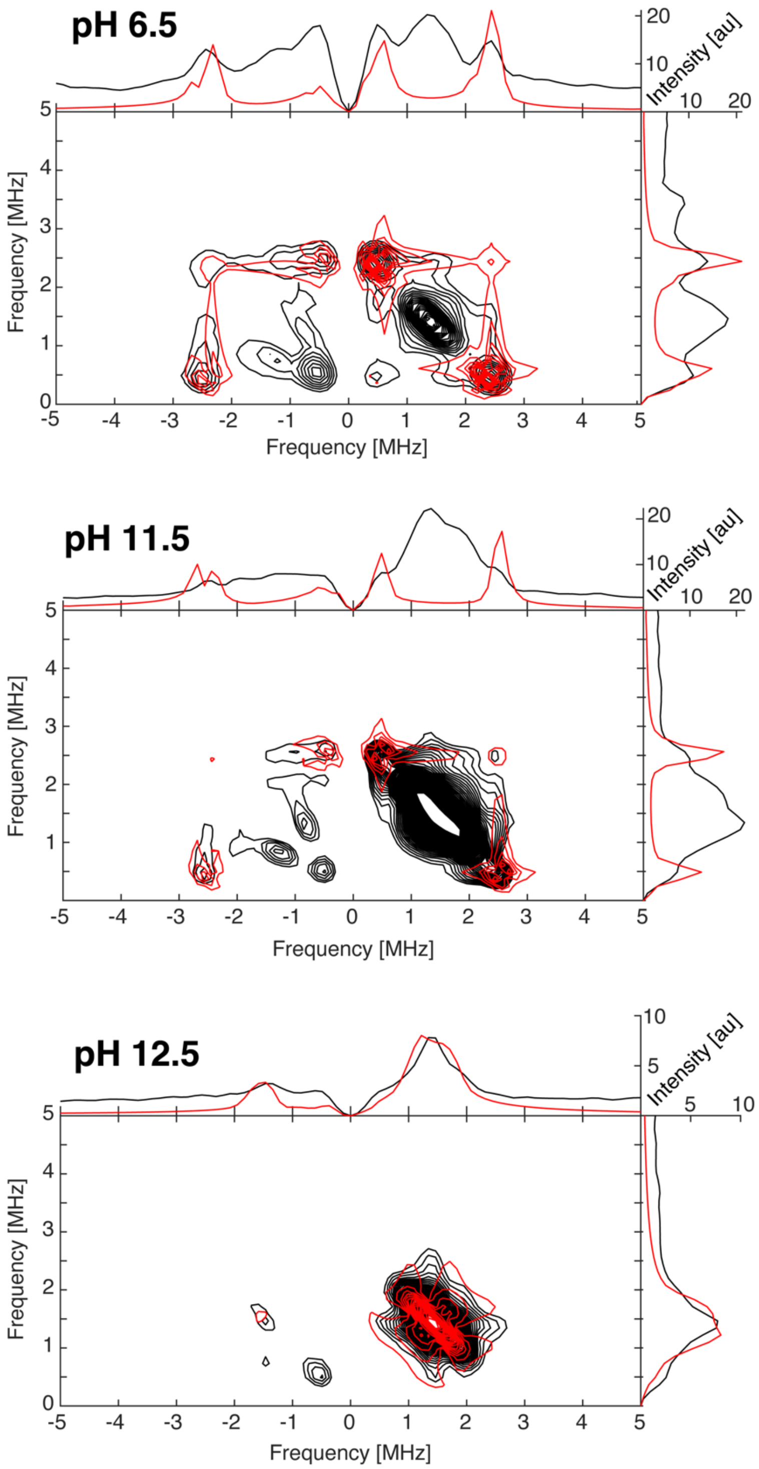

Figure S18. ^{15}N HYSCORE simulations (red) of the LPMO at three pH values (raw data in black). The conditions are the same as **Figure 4c.** Skyline projections are included at the top and right sides of each contour plot. Simulations of pH 6.5 and pH 11.5 contain two ^{15}N nuclei, with hyperfine tensors equal to the Model C ^{14}N ESEEM fits, scaled by the nuclear gyromagnetic ratio ($|g_n{}^{15}N/g_n{}^{14}N| = 1.4028$). The HYSCORE simulation of pH 12.5 contains only one ^{15}N nuclei, **A** = [-0.3, -0.3, 0.9] MHz.

Coordinates of Optimized Geometries (DFT calculated)

Coordinates of $[Cu(imidH)_4]^{2+}$

Cu	0.026169000	-0.644587000	2.706518000
N	0.613144000	-2.563153000	2.692482000
C	1.742131000	-3.022494000	2.175413000
C	-0.073848000	-3.647442000	3.200578000
C	0.664107000	-4.773027000	2.982020000
N	1.802101000	-4.354618000	2.332137000
H	2.509474000	-2.433505000	1.703838000
H	2.558963000	-4.945095000	2.019615000
H	0.488877000	-5.805997000	3.224237000
H	-1.033194000	-3.540450000	3.674891000
N	-1.797613000	-1.193126000	2.062609000
C	-2.062029000	-2.032469000	1.073734000
C	-3.008367000	-0.776737000	2.579189000
C	-4.011798000	-1.384425000	1.884094000
N	-3.391166000	-2.167200000	0.938334000
H	-1.337672000	-2.545199000	0.465119000
H	-3.851162000	-2.749054000	0.253545000
H	-5.082182000	-1.333511000	1.974483000
H	-3.067590000	-0.080906000	3.397224000
N	-0.547172000	1.278892000	2.678516000
C	-1.185557000	1.890129000	1.692713000
C	-0.363539000	2.208024000	3.683312000
C	-0.904550000	3.394472000	3.285150000
N	-1.413367000	3.170594000	2.026877000
H	-1.490750000	1.444995000	0.761667000
H	-1.877592000	3.852682000	1.445123000
H	-0.973205000	4.351952000	3.769829000
H	0.137897000	1.959328000	4.601649000
N	1.835716000	-0.105238000	3.402713000
C	2.595842000	0.875141000	2.940527000
C	2.524029000	-0.700372000	4.441142000
C	3.715977000	-0.057744000	4.599624000
N	3.740603000	0.928157000	3.640706000
H	2.349459000	1.542537000	2.133041000
H	4.491121000	1.585235000	3.485879000
H	4.527331000	-0.207172000	5.289433000
H	2.119137000	-1.535084000	4.985093000

Coordinates of [Cu(imid)$_4$]$^{2-}$

Cu	0.024339000	-0.652243000	2.722222000
N	0.603748000	-2.570068000	2.685451000
C	1.639369000	-3.099109000	2.004547000
C	0.051731000	-3.621833000	3.370602000
C	0.787749000	-4.747717000	3.070054000
N	1.796234000	-4.412649000	2.194367000
H	2.283296000	-2.500399000	1.377191000
H	0.652336000	-5.759234000	3.424124000
H	-0.815147000	-3.499295000	3.999773000
N	-1.816334000	-1.192144000	2.099443000
C	-2.148320000	-2.229977000	1.300753000
C	-3.009129000	-0.572882000	2.387105000
C	-4.008590000	-1.271282000	1.748112000
N	-3.456446000	-2.324416000	1.051297000
H	-1.410087000	-2.916444000	0.916560000
H	-5.072375000	-1.082766000	1.748515000
H	-3.054898000	0.308704000	3.004386000
N	-0.533761000	1.272574000	2.689338000
C	-0.989074000	1.978877000	1.635838000
C	-0.573994000	2.143302000	3.747606000
C	-1.056917000	3.346522000	3.279963000
N	-1.316546000	3.240212000	1.931825000
H	-1.088591000	1.546544000	0.650976000
H	-1.229544000	4.263877000	3.823632000
H	-0.249915000	1.854529000	4.734499000
N	1.835912000	-0.121090000	3.431752000
C	2.518524000	1.017364000	3.181082000
C	2.657095000	-0.850147000	4.257943000
C	3.799639000	-0.111886000	4.470477000
N	3.711625000	1.076032000	3.777227000
H	2.115823000	1.802333000	2.560172000
H	4.663690000	-0.359922000	5.069726000
H	2.376226000	-1.826032000	4.617315000

^{14,15}N ENDOR Spectroscopy and Differentiation between case A and case B

Furthermore, this enrichment decreases the number of features in the EPR spectrum (following the $2nI+1$ splitting pattern for n equivalent nuclei), yielding sharper superhyperfine features.

Both the ^{14}N and ^{15}N ENDOR spectrum of **LPMO-6.5** at low magnetic field (g_1) probes a single crystal-like position (approximately one direction of the molecular frame) of the field-frequency pattern[30,31] and shows one single ENDOR doublet ($\nu_\pm$), indicating that all three coordinating nitrogen nuclei have similar A_1 hyperfine coupling values along g_1 with no/minimal quadrupole splitting for the ^{14}N nuclei (**Figure 5** and **S19**). This is similar to the ^{14}N ENDOR spectra measured along $g_1/g_\parallel$ of other proteins with histidine-coordinated copper centers.[32,33] At higher fields, this doublet evolves into two sets of signals, best observed in the ^{15}N ENDOR field-frequency pattern **(Figure S19**). Along g_3, the additional ^{14}N quadrupole splitting is quite pronounced resulting in a complex splitting pattern (**Figure 5**). The resolution of the ^{14}N quadrupole features for **LPMO-6.5** is a distinct achievement compared to previous copper ^{14}N ENDOR studies that concluded the quadrupole splitting of the histidyl nitrogen was not observable.[32] This achievement for *Sm*AA10A is ascribed to the rhombic nature of the **g**-tensor for **LPMO-6.5** offering clear g-separation and therefore almost single crystal-like behavior along both edges of the EPR spectrum (g_1 and g_3).

The field-frequency pattern of both **LPMO-6.5** (**Figure 5** and **S19**) and **^{15}N-LPMO-6.5** (**Figure S19**) can be simulated under consideration of three strongly coupled nitrogen nuclei, where common hyperfine tensors are considered (scaled by the gyromagnetic ratio $\gamma(^{15}N)/\gamma(^{14}N)\approx1.4$) and subsequent determination of the nuclear quadrupole of these nuclei is possible for **LPMO-6.5**. The three obtained hyperfine tensors are mostly axial with dominant isotropic character and comparable to one another in magnitude and their degree of anisotropy (**Table 3**). Most importantly, the tensors vary regarding the direction of their largest element, where two of them have their largest hyperfine component along g_3, while the maximal hyperfine of the third nitrogen points along g_2. The maximum hyperfine component aligns roughly with the Cu-N bond direction, allowing us to assign an approximate molecular frame that is collinear with the **g**-tensor ($g_1 \parallel z$; $g_2 \parallel$ Cu-N^{amine}; $g_3 \parallel$ Cu-N^{imid}). This frame forms a picture where the two equivalently oriented nitrogen tensors are assigned to the two *trans*-positioned imidazole nitrogen nuclei (**N^{imid}-1** and **N^{imid}-2**), leaving the third tensor with $A_{max} \parallel g_2$ consequently ascribed to the amine nitrogen **N^{amine}**.

The nuclear quadrupole tensors of all three ^{14}N nitrogen nuclei are also well resolved, with the tensor solved collinear to the **g**-tensor (i.e. no Euler angles applied). The quadrupole tensors exhibit a significant amount of rhombicity and are comparable to one another in their magnitude with P_{max} values from 0.9 to 1.1 MHz. Notably, for all three tensors the largest absolute value points along the assigned Cu-N bond, aligning with the A_{max} of each nucleus. P_{min} is oriented along g_1, approximately normal to the imidazole plane, as previously shown for other copper coordinated imidazole rings.[28,34]

The ^{14}N and ^{15}N ENDOR spectra of **LPMO-11.5** (**Figure 5** and **S19**) show a different field-frequency pattern compared to **LPMO-6.5**, in part due to the axial **g**-tensor of **LPMO-11.5** and its different orientation selection pattern (**Figure 5**). ENDOR spectra collected along $g_{\parallel}(g_1)$ still selectively probe a single orientation for the axial **g**-tensor of **LPMO-11.5**, along the z-direction orthogonal to the histidine brace plane. Once again, the ^{14}N ENDOR spectrum along this direction exhibits only one signal doublet implying similar hyperfine interactions for all three nuclei, without significant nuclear quadrupole splitting. At higher magnetic fields, less distinct $^{14}N/^{15}N$ features are observed in the $g_{\perp}$ region and the spectra are generally broader than what is seen for (**^{15}N-)LPMO-6.5**. This can be attributed to the fact, that no single crystal-like orientation is achieved, and both the g_2 and g_3 direction are probed. In conjunction with information on the hyperfine interaction for **^{15}N-LPMO-11.5** (**Figure S19**), and assuming the same relative orientations of the three hyperfine tensors as determined for **LPMO-6.5**, the full field-frequency pattern can be satisfactorily reproduced, using the same nuclear quadrupole interactions and comparable hyperfine tensors to the ones that were determined for **LPMO-6.5** (**Figure 5** and **Table 3**).

The ^{14}N ENDOR response of **LPMO-12.5** collected at the field position associated with a 'single crystal like' orientation along $g_{\parallel}$ is also depicted in **Figure 5**. When overlaid with the ^{14}N ENDOR responses of **LPMO-6.5** and **LPMO-11.5**, dramatic differences for **LPMO-12.5** are observed (**Figure 6a**). The total breadth of the **LPMO-12.5** ENDOR response is much larger compared to the spectra collected for lower pH samples, with intensity observed at frequencies as low as 10 MHz and up to 27 MHz. The ^{14}N and ^{15}N ENDOR response along $g_{\parallel}$ can be modelled as the sum of three equally intense gaussian doublet pairs (split by $2\nu_n$) (**Figure 6b**). Each signal pair is centered at half of the observed hyperfine coupling $A(^{14}N)$. One smaller coupling of ~28.5 MHz (assigned to **N^{amine}**), and two larger but more similar couplings of 40 and 44 MHz are observed (assigned to **N^{imid}-1** and **N^{imid}-2**). No quadrupole splitting is detected. The ^{15}N ENDOR response shifts to higher frequency compared to the ^{14}N ENDOR because of the increased hyperfine coupling values and Larmor frequency as determined by the nuclear gyromagnetic ratio of the two isotopes, eq. 4. The ^{15}N ENDOR spectrum along $g_{\parallel}$ fits reasonably well with three Gaussian doublets (**Figure 6b)**. A slightly narrower linewidth is required for the ^{15}N ENDOR fit, reflecting the lack of quadrupole interaction for the ^{15}N-enriched sample. The ^{15}N ENDOR spectrum also resolves the two ν_+ features from two different atoms, seen at the highest frequency peak around ~ 34 MHz.

At the higher magnetic field positions, the ^{14}N ENDOR response of **LPMO-12.5** spans a significantly larger frequency range with more clearly defined features compared to **LPMO-11.5** (**Figure 5**). The spectrum is associated with one smaller component, with a ^{14}N hyperfine coupling as low as $A(^{14}N)$ ~27 MHz, and with contributions as large as $A(^{14}N)$ ~ 50 MHz. The two-dimensional field-frequency data set (**Figure 5**) can be simulated under consideration of three nitrogen nuclei. The coordinating imidazole nitrogens, **N^{imid}-1** and **N^{imid}-2**, may be modelled with larger, axial ^{14}N hyperfine tensors with their largest components pointing along g_3 (**Table 3**) in agreement with the previously established coordination frame for **LPMO-6.5** and **LPMO-11.5**. The increased span of couplings and width of frequencies observed for signals of **LPMO-12.5** indicates an

increase in anisotropy of the hyperfine tensors of the individual nitrogens and/or increased separation of their character.

To complete the simulation and reproduce the observed ENDOR pattern, the consideration of a third strongly coupled nitrogen nucleus is required, accounting for the lower frequency features appearing between 7 and 14 MHz (^{14}N ENDOR spectra) (**Figure 5**). These features are attributed to hyperfine couplings of ~27 MHz with an additional quadrupole splitting of $3P \sim 3$ MHz along g_3. This is in agreement with the lone ν_- feature observed at ~15 MHz in the ^{15}N ENDOR spectra (**Figure 6b**). Although the amine nitrogen displays distinct features along these positions, the axial nature of the **g**-tensor complicates the determination of the A_2 hyperfine coupling along g_2. Based on its presumed axial nature, the hyperfine tensor of the amine must take either the form of [*min, min, max*] or [*min, max, min*] MHz. Given that the *N*-terminal amine principle (A_{max}) is expected to be oriented along the Cu-N^{amine} interaction, as we have already shown above for **LPMO-6.5** and **LPMO-11.5**, the latter case of [*min, max, min*] is expected.

For the fitting of the ENDOR spectra of **LPMO-12.5** and **^{15}N-LPMO-12.5**, two distinct parameter sets yield reasonable fits of the experimental data (**Figure 5** and **Table 3**). The resulting spin Hamiltonian parameters were used to simulate the corresponding ^{14}N and ^{15}N superhyperfine features of the EPR spectra (**Figure S20**). The first parameter set ('**case A**') has a relatively small A_2 value of 37 MHz, while the second parameter set ('**case B**') has a larger A_2 value of 45 MHz. Both cases result in highly similar and satisfactory ENDOR simulations with the most significant difference observed at intermediate field positions. In the two presented cases, the magnitude of A_2, and thereby the degree of hyperfine anisotropy of the tensor, modulates the breadth of the amine's ENDOR response, leading to only subtle changes of the summed simulation's lineshape (**Figure 5**). As the A_2 and A_3 hyperfine components for each nitrogen component are observed in the superhyperfine of the $g_\perp$ feature of the EPR spectrum, it is important to evaluate the reproduction of the EPR spectra in parallel to the ENDOR pattern. It is observed by EPR simulation that each case simulates the nitrogen superhyperfine pattern well, with no significant differences observed or a clear favorable selection being possible (**Figure S20**).

Notably, whereas the ^{14}N ENDOR spectra of **LPMO-6.5** and **LPMO-11.5** can be reproduced with the same nuclear quadrupole tensors, the simulation of **LPMO-12.5** (in both presented cases) requires the alteration of the applied nqi for all three nuclei, including generally larger nuclear quadrupole constants and mostly higher rhombicities (**Table 3**). The substantial changes of the hyperfine and nuclear quadrupole interactions for **LPMO-12.5** compared **to LPMO-6.5** and **LPMO-11.5** stress the general electronic changes at the histidine brace as the pH is increased above pH 11.5, which is associated with the ligand deprotonations.

It is challenging, if not a fool's errand, to select between the two cases presented based on just the EPR and ENDOR simulations (**Figure 5** and **S20**). This emphasizes some of the challenges in determining individual hyperfine values from the EPR spectra alone, and the multiple local minima that may be achieved in fitting. However, the combined EPR and ENDOR analysis sets clear bounds for the nitrogen hyperfine parameters for

LPMO-12.5 and - despite the lack of a clear and favored case **-** reveals distinguishing trends and characteristics for this enzyme state, compared to the enzyme at lower pH.

In both presented cases the isotropic component of the hyperfine coupling a_{iso} decreases compared to the two samples at lower pH (**Table S6**), indicating a less covalent Cu-N-σ interaction with the dx^2-y^2 SOMO. The dipolar component, *t*, on the other hand, shows a slight decrease in **case A**, while it increases a large amount in **case B**. Generally, this anisotropic component is a reporter of the ligand p orbital spin population (and distribution), when excluding through-space dipolar contributions. The two cases thus represent different possible changes in the magnetic structure at the amine's nitrogen atom. **Case A** represents a general decrease in the p orbital spin population, consistent with an elongated Cu-N interaction and less overall covalent interaction. **Case B** exhibits an increase in p orbital spin population relative to pH 11.5, a possible indication of either increased charge or lone pair formation. The possibility of lone pair formation must be considered carefully.

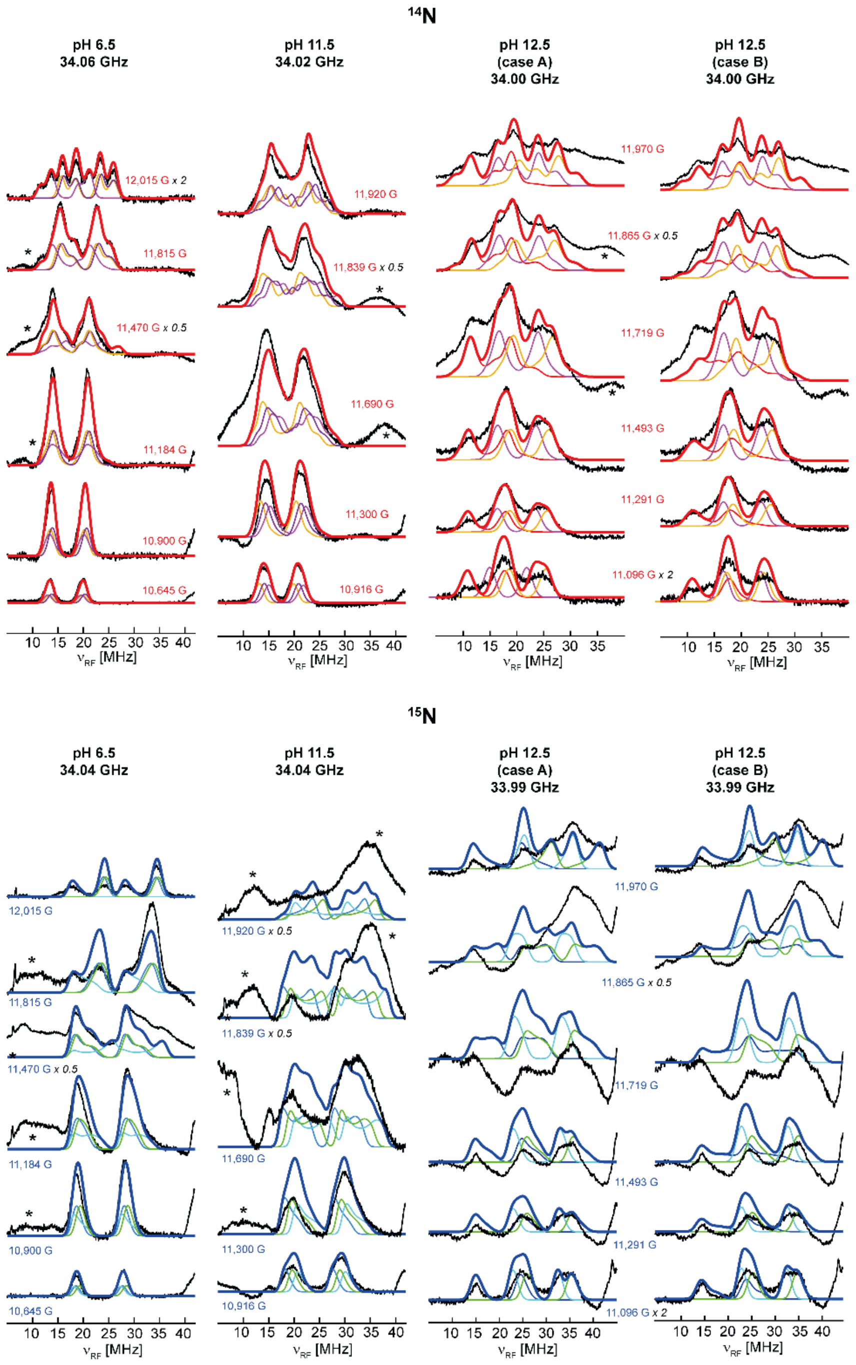

Figure S19. ^{14}N and ^{15}N Davies ENDOR spectra.

Q-band ^{14}N (top) and ^{15}N (bottom) Davies ENDOR spectra of *Sm*AA10A at pH 6.5, 11.5 and 12.5 in black with simulations for the three individual nitrogen nuclei (thinner, colored lines) and their sum (thick, colored line). For pH 12.5 simulations, **case A** and **case B** are presented (see main text for details). Additional intensities (*) in the spectra are tentatively assigned to copper hyperfine and nitrogen resonances and are more pronounced in the ^{15}N-^{63}Cu-enriched samples. The samples at pH 11.5 and 12.5 are mostly affected at intermediate and high field, the one at pH 6.5 only at its intermediate field (~g_2). Notably, the copper hyperfine along the corresponding g-values is comparably small of ~25 – 100 MHz, which shifts the observable copper hyperfine resonances in the region of ~1 – 65 MHz. EPR parameters are listed in **Table S1** and **S6**. Experimental conditions are reported in the Experimental Section. Gaussian broadenings of 1.5 mT (**^{14}N-LPMO-6.5**, **^{14,15}N-LPMO-11.5**), 1.7 mT (**^{15}N-LPMO-6.5**) and 2.2 mT (**^{14,15}N-LPMO-12.5**) were applied.

Table S6. Hyperfine and nuclear quadrupole parameters of the coordinated nitrogen nuclei.
Derived spin densities obtained through simulation of ^{14}N and ^{15}N ENDOR spectra (**Figure S19**) with three nitrogen nuclei, **N^{amine}**, **N^{imid}-1** and **N^{imid}-2**. All parameters are reported for the ^{14}N isotope.

	**A** = [A_1, A_2, A_3] [MHz]	a_{iso} a) [MHz]	t a) [MHz]	$\rho(s)$ b) [%]	$\rho(p)$ b) [%]	ρ_{total} b) [%]	**P** = [P_1, P_2, P_3] [MHz]	e^2Qq/h [MHz] c)	η
**LPMO-6.5**									
**N^{amine}**	32.0 44.9 32.2	36.4	4.3	2.0	7.6	25.3	0.3 -1.1 0.8	-2.2	0.45
**N^{imid}-1**	32.8 32.5 42.6	36.0	3.3	2.0	5.9		0.3 0.7 -1.0	-2.0	0.4
**N^{imid}-2**	33.9 33.0 43.2	36.7	3.3	2.0	5.8		0.2 0.7 -0.9	-1.8	0.56
**LPMO-11.5**									
**N^{amine}**	35.5 48.0 35.5	39.7	4.2	2.2	7.4	26.1	0.3 -1.1 0.8	-2.2	0.45
**N^{imid}-1**	32.0 32.0 42.0	35.3	3.3	2.0	6.0		0.3 0.7 -1.0	-2.0	0.4
**N^{imid}-2**	34.0 34.0 45.0	37.7	3.7	2.0	6.5		0.2 0.7 -0.9	-1.8	0.56
**LPMO-12.5 (case A)**									
**N^{amine}**	27.5 37.0 27.0	30.5	3.25	1.7	5.8	21.7	0.4 -1.7 1.3	-3.4	0.52
**N^{imid}-1**	39.0 39.0 44.5	40.8	1.8	2.3	3.3		0.3 1.1 -1.4	-2.8	0.57
**N^{imid}-2**	43.0 43.0 53.0	46.3	3.3	2.6	6.0		0.3 0.9 -1.2	-2.4	0.5
**LPMO-12.5 (case B)**									
**N^{amine}**	27.0 45.0 28.0	33.3	5.8	1.8	10.4	25.4	0.3 -1.5 1.2	-3.0	0.6
**N^{imid}-1**	40.0 39.0 44.0	41.0	1.5	2.2	3.0		0.3 1.0 -1.3	-2.6	0.53
**N^{imid}-2**	43.0 42.0 52.0	45.6	3.1	2.5	5.5		0.2 1.3 -1.5	-3.0	0.73

a) The isotropic (a_{iso}) and the anisotropic (t) hyperfine contributions are calculated as follows: $a_{iso} = (A_1+A_2+A_3)/3$ and $\mathbf{T} = \mathbf{A} - a_{iso}$ with $\mathbf{T} = [-t, -t, 2t]$.

b) The s and p orbital spin populations are estimated from a_{iso} and t, by dividing through the isotropic (a_0) and anisotropic (b_0) coupling constants, respectively. The values for a_0 (1,811 MHz) and b_0 (56 MHz, = 138.8 MHz*2/5) were taken from Ref[35]. The total spin population ρ_{total} of the histidine brace is calculated by summing up the s and p orbital spin populations for all three nitrogen nuclei.

c) e^2qQ/h is estimated from equation 3, using only P_{max}.

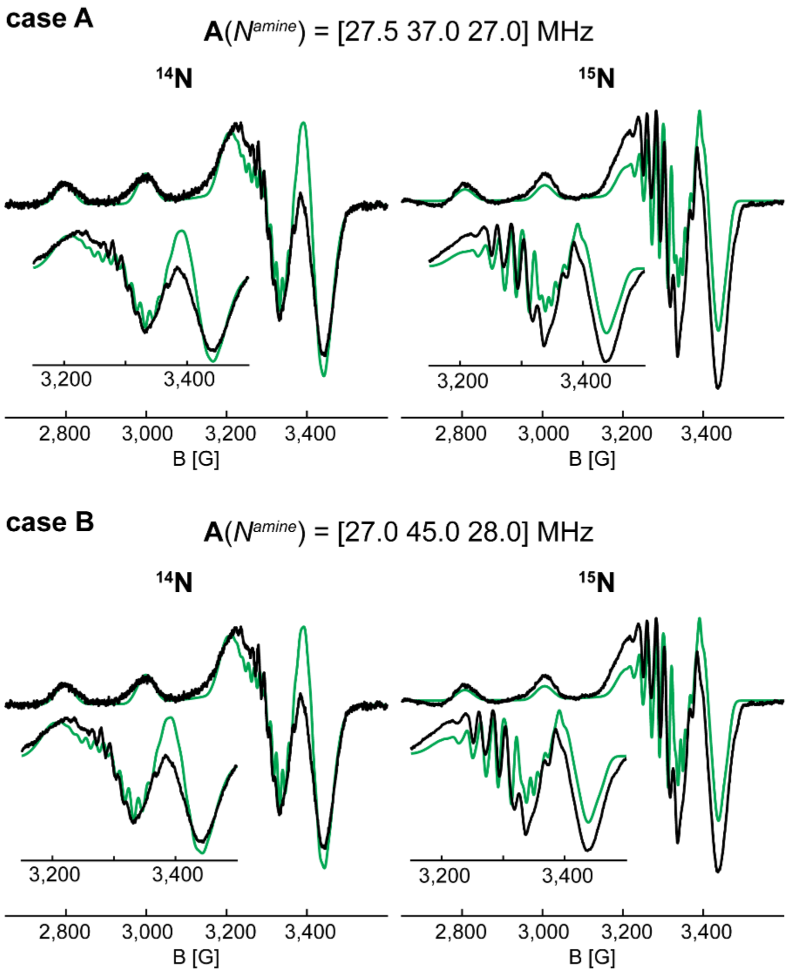

Figure S20. X-band EPR simulations of LPMO-12.5 **case A** and **case B**.

CW X-band EPR spectra of *Sm*AA10A (left) and ^{15}N-*Sm*AA10A (right) at pH 12.5 in black with simulations in green. Simulations of both discussed cases are presented, with **case A** (**A**(N^{amine}) = [27.5 37.0 27.0] MHz) in the top figure and **case B** (**A**(N^{amine}) = [27.0 45.0 28.0] MHz) in the bottom figure. Experimental conditions and additional simulation parameters are reported in the Experimental Section and **Tables S1** (**g**-tensors and copper hyperfine) and **S6** (nitrogen hyperfine).

References

(1) Shrager, R. I. Chemical Transitions Measured by Spectra and Resolved Using Singular Value Decomposition. *Chemom. Intell. Lab. Syst.* **1986**, *1*, 59–70. DOI: 10.1016/0169-7439(86)80026-0.

(2) Knott, G. D. Analysis of Absorption Spectra-Titration Data. *https://www.civilized.com/pdffiles/svd.pdf.* pp 1–10. Accessed June 28, 2024.

(3) Stepnov, A. A.; Forsberg, Z.; Sørlie, M.; Nguyen, G. S.; Wentzel, A.; Røhr, Å. K.; Eijsink, V. G. H. Unraveling the Roles of the Reductant and Free Copper Ions in LPMO Kinetics. *Biotechnol. Biofuels* **2021**, *14* (1), 1–14. DOI: 10.1186/s13068-021-01879-0.

(4) Østby, H.; Tuveng, T. R.; Stepnov, A. A.; Vaaje-Kolstad, G.; Forsberg, Z.; Eijsink, V. G. H. Impact of Copper Saturation on Lytic Polysaccharide Monooxygenase Performance. *ACS Sustainable Chem. Eng.* **2023**, *11*, 15566–15576. DOI: 10.1021/acssuschemeng.3c03714.

(5) Stepnov, A. A.; Eijsink, V. G. H.; Forsberg, Z. Enhanced in Situ H_2O_2 Production Explains Synergy between an LPMO with a Cellulose-Binding Domain and a Single-Domain LPMO. *Sci. Rep.* **2022**, *12* (6129), 1–11. DOI: 10.1038/s41598-022-10096-0.

(6) Müller, G.; Chylenski, P.; Bissaro, B.; Eijsink, V. G. H.; Horn, S. J. The Impact of Hydrogen Peroxide Supply on LPMO Activity and Overall Saccharification Efficiency of a Commercial Cellulase Cocktail. *Biotechnol. Biofuels* **2018**, *11* (209), 1–17. DOI: 10.1186/s13068-018-1199-4.

(7) Golten, O.; Ayuso-Fernández, I.; Hall, K. R.; Stepnov, A. A.; Sørlie, M.; Røhr, Å. K.; Eijsink, V. G. H. Reductants Fuel Lytic Polysaccharide Monooxygenase Activity in a pH-Dependent Manner. *FEBS Lett.* **2023**, *597*, 1363–1374. DOI: 10.1002/1873-3468.14629.

(8) Bissaro, B.; Streit, B.; Isaksen, I.; Eijsink, V. G. H.; Beckham, G. T.; DuBois, J. L.; Røhr, Å. K. Molecular Mechanism of the Chitinolytic Peroxygenase Reaction. *Proc. Natl. Acad. Sci. U. S. A.* **2020**, *117* (3), 1504–1513. DOI: 10.1073/pnas.1904889117.

(9) Jensen, M. S.; Klinkenberg, G.; Bissaro, B.; Chylenski, P.; Vaaje-Kolstad, G.; Kvitvang, H. F.; Nærdal, G. K.; Sletta, H.; Forsberg, Z.; Eijsink, V. G. H. Engineering Chitinolytic Activity into a Cellulose-Active Lytic Polysaccharide Monooxygenase Provides Insights into Substrate Specificity. *J. Biol. Chem.* **2019**, *294* (50), 19349–19364. DOI: 10.1074/jbc.ra119.010056.

(10) Munzone, A.; Pujol, M.; Tamhankar, A.; Joseph, C.; Mazurenko, I.; Réglier, M.; Jannuzzi, S. A. V.; Royant, A.; Sicoli, G.; DeBeer, S.; Orio, M.; Simaan, A. J.; Decroos, C. Integrated Experimental and Theoretical Investigation of Copper Active Site Properties of a Lytic Polysaccharide Monooxygenase from *Serratia Marcescens*. *Inorg. Chem.* **2024**, *63* (24), 11063–11078. DOI: 10.1021/acs.inorgchem.4C00602.

(11) Chaplin, A. K.; Wilson, M. T.; Hough, M. A.; Svistunenko, D. A.; Hemsworth, G. R.; Walton, P. H.; Vijgenboom, E.; Worrall, J. A. R. Heterogeneity in the Histidine-Brace Copper Coordination Sphere in Auxiliary Activity Family 10 (AA10) Lytic Polysaccharide Monooxygenases. *J. Biol. Chem.* **2016**, *291* (24), 12838–12850. DOI: 10.1074/jbc.m116.722447.

(12) Munzone, A.; El Kerdi, B.; Fanuel, M.; Rogniaux, H.; Ropartz, D.; Réglier, M.; Royant, A.; Simaan, A. J.; Decroos, C. Characterization of a Bacterial Copper-Dependent Lytic Polysaccharide Monooxygenase with an Unusual Second Coordination Sphere. *FEBS J.* **2020**, *287* (15), 3298–3314. DOI: 10.1111/febs.15203.

(13) Serra, I.; Piccinini, D.; Paradisi, A.; Ciano, L.; Bellei, M.; Bortolotti, C. A.; Battistuzzi, G.; Sola, M.; Walton, P. H.; Di Rocco, G. Activity and Substrate Specificity of Lytic Polysaccharide

Monooxygenases: An ATR FTIR-Based Sensitive Assay Tested on a Novel Species from *Pseudomonas Putida*. *Protein Sci.* **2022**, *31* (3), 591–601. DOI: 10.1002/pro.4255.

(14) Lindley, P. J.; Parkin, A.; Davies, G. J.; Walton, P. H. Mapping the Protonation States of the Histidine Brace in an AA10 Lytic Polysaccharide Monooxygenase Using CW-EPR Spectroscopy and DFT Calculations. *Faraday Discuss.* **2022**, *234*, 336–348. DOI: 10.1039/d1fd00068c.

(15) Rogelio, J. G.; Drosou, M.; Bertaina, S.; Decroos, C.; Simaan, A. J.; Pantazis, D. A.; Orio, M. Decoding the Ambiguous Electron Paramagnetic Resonance Signals in the Lytic Polysaccharide Monooxygenase from *Photorhabdus Luminescens*. *Inorg. Chem.* **2022**, *61* (20), 8022–8035. DOI: 10.1021/acs.inorgchem.2c00766.

(16) Garribba, E.; Micera, G. The Determination of the Geometry of Cu(II) Complexes. An EPR Spectroscopy Experiment. *J. Chem. Educ.* **2006**, *83* (8), 1229–1232. DOI: 10.1021/ed083p1229.

(17) Bissaro, B.; Isaksen, I.; Vaaje-Kolstad, G.; Eijsink, V. G. H.; Røhr, Å. K. How a Lytic Polysaccharide Monooxygenase Binds Crystalline Chitin. *Biochemistry* **2018**, *57* (12), 1893–1906. DOI: 10.1021/acs.biochem.8b00138.

(18) Peisach, J.; Blumberg, W. E. Structural Implications Derived from the Analysis of Electron Paramagnetic Resonance Spectra of Natural and Artificial Copper Proteins. *Arch. Biochem. Biophys.* **1974**, *165* (2), 691–708. DOI: 10.1016/0003-9861(74)90298-7.

(19) Froncisz, W.; Hyde, J. S. Broadening by Strains of Lines in the G-Parallel Region of Cu^{2+} EPR Spectra. *J. Chem. Phys.* **1980**, *73* (7), 3123–3131. DOI: 10.1063/1.440548.

(20) Hyde, J. S.; Froncisz, W. The Role of Microwave Frequency in EPR Spectroscopy of Copper Complexes. *Ann. Rev. Biophys. Bioeng.* **1982**, *11*, 391–417. DOI: 10.1146/annurev.bb.11.060182.002135.

(21) Shane, J. J.; van der Heijden, P. A. A. W.; Reijerse, E. J.; de Boer, E. An ESEEM Investigation of Single Crystals and Powders of Copper-Doped L-Histidine Hydrochloride Monohydrate. *Appl. Magn. Reson.* **1994**, *6* (3), 427–454. DOI: 10.1007/bf03162635.

(22) McCracken, J.; Pember, S.; Benkovic, S. J.; Villafranca, J. J.; Miller, R. J.; Peisach, J. Electron Spin-Echo Studies of the Copper Binding Site in Phenylalanine Hydroxylase from *Chromobacterium Violaceum*. *J. Am. Chem. Soc.* **1988**, *110* (4), 1069–1074. DOI: 10.1021/ja00212a012.

(23) Stoll, S.; Britt, R. D. General and Efficient Simulation of Pulse EPR Spectra. *Phys. Chem. Chem. Phys.* **2009**, *11* (31), 6614–6625. DOI: 10.1039/B907277B.

(24) Sun, L.; Hernandez-Guzman, J.; Warncke, K. OPTESIM, a Versatile Toolbox for Numerical Simulation of Electron Spin Echo Envelope Modulation (ESEEM) That Features Hybrid Optimization and Statistical Assessment of Parameters. *J. Magn. Reson.* **2009**, *200* (1), 21–28. DOI: 10.1016/j.jmr.2009.05.012.

(25) Gunderson, W. A.; Hernández-Guzmán, J.; Karr, J. W.; Sun, L.; Szalai, V. A.; Warncke, K. Local Structure and Global Patterning of Cu^{2+} Binding in Fibrillar Amyloid-β [Aβ(1-40)] Protein. *J. Am. Chem. Soc.* **2012**, *134* (44), 18330–18337. DOI: 10.1021/ja306946q.

(26) Jiang, F.; Peisach, J.; McCracken, J. Nuclear Quadrupole Interactions in Copper(II)-Diethylenetriamine-Substituted Imidazole Complexes and in Copper(II) Proteins. *J. Am. Chem. Soc.* **1990**, *112* (25), 9035–9044. DOI: 10.1021/ja00181a002.

(27) McCracken, J.; Peisach, J.; Cote, C. E.; McGuirl, M. A.; Dooley, D. M. Pulsed EPR Studies of the Semiquinone State of Copper-Containing Amine Oxidases. *J. Am. Chem. Soc.* **1992**, *114* (10), 3715–3720. DOI: 10.1021/ja00036a021.

(28) Ashby, C. I. H.; Cheng, C. P.; Brown, T. L. ^{14}N Nuclear Quadrupole Resonance Spectra of Coordinated Imidazole. *J. Am. Chem. Soc.* **1978**, *100* (19), 6057–6063. DOI: 10.1021/ja00487a014.

(29) Reijerse, E. J.; Keijzers, C. P. Model Calculations of Frequency-Domain ESEEM Spectra of Disordered Systems. *J. Magn. Reson.* **1987**, *71* (1), 83–96. DOI: 10.1016/0022-2364(87)90129-6.

(30) Hoffman, B. M.; Venters, R. A.; Martinsen, J. General Theory of Polycrystalline ENDOR Patterns. Effects of Finite EPR and ENDOR Component Linewidths. *J. Magn. Reson.* **1985**, *62* (3), 537–542. DOI: 10.1016/0022-2364(85)90225-2.

(31) Hoffman, B.; Martinsen, J.; Venters, R. A. General Theory of Polycrystalline ENDOR Patterns. g and Hyperfine Tensors of Arbitrary Symmetry and Relative Orientation. *J. Magn. Reson.* **1984**, *59*, 11–123. DOI: 10.1016/0022-2364(84)90287-7.

(32) Werst, M. M.; Davoust, C. E.; Hoffman, B. M. Ligand Spin Densities in Blue Copper Proteins by Q-Band ^{1}H and ^{14}N ENDOR Spectroscopy. *J. Am. Chem. Soc.* **1991**, *113* (5), 1533–1538. DOI: 10.1021/ja00005a011.

(33) Roberts, J. E.; Cline, J. F.; Lum, V.; Freeman, H.; Gray, H. B.; Peisach, J.; Reinhammar, B.; Hoffman, B. Comperative ENDOR Study of Six Blue Copper Proteins. *J. Am. Chem. Soc.* **1984**, *106*, 5324–5330. DOI: 10.1021/ja00330a048.

(34) Veselov, A.; Olesen, K.; Sienkiewicz, A.; Shapleigh, J. P.; Scholes, C. P. Electronic Structural Information from Q-Band ENDOR on the Type 1 and Type 2 Copper Liganding Environment in Wild-Type and Mutant Forms of Copper-Containing Nitrite Reductase. *Biochemistry* **1998**, *37*, 6095–6105. DOI: 10.1021/bi971604r.

(35) Morton, J. R.; Preston, K. F. Atomic Parameters for Paramagnetic Resonance Data. *J. Magn. Reson.* **1978**, *30* (3), 577–582. DOI: 10.1016/0022-2364(78)90284-6.

.
